# Chitosan‐induced modulation of secondary metabolism and stress tolerance in *Salvia rosmarinus* under combined drought and heat stress

**DOI:** 10.1002/jsfa.70652

**Published:** 2026-04-09

**Authors:** Inês Mansinhos, Sandra Gonçalves, Raquel Rodríguez‐Solana, Efrén Pérez‐Santín, María I Fernández‐Marín, Emma Cantos‐Villar, Anabela Romano

**Affiliations:** ^1^ MED – Mediterranean Institute for Agriculture, Environment and Development, and CHANGE – Global Change and Sustainability Institute, Faculdade de Ciências e Tecnologia Universidade do Algarve, Campus de Gambelas Faro Portugal; ^2^ Department of Agroindustry and Food Quality, Andalusian Institute of Agricultural and Fisheries Research and Training (IFAPA) Rancho de La Merced Center Cádiz Spain; ^3^ Escuela Superior de Ingeniería y Tecnología (ESIT) Universidad Internacional de La Rioja – UNIR Logroño Spain

**Keywords:** *Rosmarinus officinalis*, HPLC, phenolics, GC‐MS, terpenoids, biostimulation

## Abstract

**BACKGROUND:**

Abiotic stresses, particularly drought and elevated temperatures, negatively affect plant physiological and metabolic processes. This study investigated the effects of drought, heat, and combined stress on *Salvia rosmarinus* (rosemary), and evaluated foliar‐applied chitosan as a biostimulant. After 3 weeks, photosynthetic pigments, osmoprotectants (proline, soluble sugars), oxidative stress indicators (hydrogen peroxide and lipid peroxidation), phenolic compounds, essential oils, and antioxidant activity were evaluated. A natural deep eutectic solvent (NADES) was used to enhance phenolic extraction sustainably.

**RESULTS:**

Combined stress intensified oxidative damage and reduced photosynthetic pigments, while elevated osmoprotectants and chlorophyll *a*/*b* ratio reflected adaptive responses. Chitosan improved pigment retention, boosted sugar levels, and mitigated oxidative damage. Stress‐exposed, chitosan‐treated plants showed increased rosmarinic acid and key monoterpenes (camphor, α‐pinene, 1,8‐cineole), indicating enhanced secondary metabolism. Antioxidant assays confirmed superior scavenging and reducing activities in chitosan‐treated plants under stress.

**CONCLUSION:**

Chitosan modulated physiological and metabolic responses in *Salvia rosmarinus*, improving resilience and phytochemical profile under abiotic stress. This is the first report demonstrating foliar‐applied chitosan's effectiveness in *Salvia rosmarinus* facing drought and heat. © 2026 The Author(s). *Journal of the Science of Food and Agriculture* published by John Wiley & Sons Ltd on behalf of Society of Chemical Industry.

## INTRODUCTION

Recurrent drought episodes and elevated temperatures, intensified by ongoing climate change, constitute major abiotic stressors that significantly impair plant growth and productivity. In response, plants initiate adaptive mechanisms such as osmoprotectant accumulation and the activation of antioxidant systems, including the biosynthesis of secondary metabolites like phenolics and terpenes.^1^, ^2^ *Salvia rosmarinus* Spenn. (formerly *Rosmarinus officinalis* Linn.), a Mediterranean evergreen shrub of the Lamiaceae family, is widely appreciated for its aromatic and medicinal properties. Rich in essential oils (EOs) and phenolic compounds, rosemary is commonly used in the food, pharmaceutical, and cosmetics industries due to its broad range of biological activities.^3^, ^4^ The European Union recognized rosemary extracts as safe natural antioxidants in 2008.^5^ However, one challenge is the extraction of phenolic compounds, often hindered by undesirable sensory properties. To overcome these limitations, alternative extraction strategies have been developed to obtain odorless and colorless antioxidant fractions, particularly enriched in phenolic diterpenes like carnosol and carnosic acid, and phenolic acids such as rosmarinic acid (RA).^6^ The solubility and recovery of these compounds are highly dependent on solvent polarity and molecular structure. In this regard, natural deep eutectic solvents (NADES) offer a promising alternative to conventional solvents. Comprising a hydrogen bond donor (HBD) and a hydrogen bond acceptor (HBA), NADES form a eutectic system with melting points lower than those of their individual components.^7^ These biodegradable, renewable, and non‐volatile solvents are compatible with pharmaceutical and food applications and capable of selectively extracting a broad spectrum of phenolics.^8^ Rosemary EOs are particularly noted for their strong antioxidant and antimicrobial activities, making them attractive agents for food preservation.^9^ However, stress conditions can alter rosemary's secondary metabolite composition. Studies reveal contradictory responses; for example, α‐pinene may increase under moderate drought^3^ or decrease under severe water deficit.^10^ Similarly, heat and drought can enhance the production of compounds like coumarins, alkaloids, and flavonoids.^11^

Given that environmental stress adversely affects productivity and bioactive compound composition, it is crucial to explore strategies that not only preserve vegetative growth but also ensure the quantity and quality of high‐value metabolites. One promising approach is the exogenous application of chitosan, a natural cationic polysaccharide derived from chitin.^12^ Recently approved as a basic substance for plant protection under Commission Implementing Regulation (EU) 2022/456, chitosan has garnered attention for its non‐toxicity, biodegradability, solubility, and diverse bioactivities.^13^ Chitosan has demonstrated positive effects on plant performance under drought stress in a range of species, including *Lavandula stoechas*,^12^ *Solanum lycopersicum*,^13^ *Calendula officinalis*,^14^ *Lallemantia royleana*,^15^ and *Salvia abrotanoides*.^16^ Yet few studies have examined its performance under combined abiotic stresses, especially in medicinal plants. This represents a significant knowledge gap, especially considering that plants in natural ecosystems are frequently exposed to multiple, concurrent abiotic stresses. Furthermore, aromatic and medicinal species remain underrepresented in combined‐stress studies, which have traditionally prioritized staple crops. Although chitosan has been officially recognized as a plant biostimulant, further research is needed to elucidate its mechanisms of action, optimize its use across diverse plant species, and assess its efficacy under multiple stress conditions. Although ample evidence supports the role of chitosan in enhancing plant tolerance to abiotic stress, especially drought, its impact on rosemary, particularly under heat and drought stress (individual and combined), has yet to be investigated. In this context, this study aims to evaluate the biochemical response (photosynthetic pigments, oxidative stress markers, and osmoprotectants) and secondary metabolic responses (phenolic and essential oil profiles) of *Salvia rosmarinus* subjected to drought, heat, and combined stress conditions, with or without foliar application of chitosan.

## EXPERIMENTAL

### Plant material and abiotic stress treatments

*Salvia rosmarinus* Spenn. plants (~30–35 cm in height), grown in 14 cm diameter plastic pots (~1.92 L), were obtained from a commercial nursery in April 2024. Rosemary plants were obtained through vegetative propagation (rooted cuttings), with four plants used per treatment. All plants were maintained under controlled growth chamber conditions (Aralab, Lisbon, Portugal) equipped with four cool white lamps (Osram L 18 W/840), under a photoperiod of 16/8 h (light/dark), for the duration of the experiment (3 weeks). Control plants were maintained at 25/18 °C and 50–60% air humidity, and were watered every 2 days. For drought treatments, the temperature was maintained at 25/18 °C, and irrigation was reduced to once every 4 days. For heat treatments, the temperature was elevated to 32/24 °C, with irrigation applied every 2 days. A combined stress treatment was applied, where the plants were simultaneously subjected to drought conditions (irrigation every 4 days) and heat stress (temperature raised to 32/24 °C). Chitosan (low molecular weight, 50 000–190 000 Da based on viscosity; viscosity 20–300 cP at 1 wt% in 1% acetic acid at 25 °C; degree of deacetylation 75–85%, Sigma‐Aldrich, Steinheim, Germany) solution was prepared by dissolving 0.05 g chitosan in 50 mL of 1% acetic acid, and distilled water was subsequently added to achieve a final concentration of 6 mg L^−1^. Plants of all treatments were sprayed (~5 mL per plant) once weekly with chitosan solution or distilled water using a hand sprayer.

Samples were collected at the end of the 3‐week period. A portion of the plant material was immediately frozen in liquid nitrogen and stored at −80 °C for subsequent analysis, while the remaining material was oven dried at 40 °C.

### Photosynthetic pigments

Photosynthetic pigments and carotenoids were assessed according to Lichtenthaler's^17^ protocol, by macerating plant material (25 mg) in 4 mL acetone. After centrifugation, the absorbance was read at 470, 644.8, and 661.6 nm using a T70+ ultraviolet (UV)–visible spectrophotometer (PG Instruments Ltd, Wibtoft, UK). The results were expressed as milligrams per gram of fresh weight.

### Oxidative stress indicators

The contents of hydrogen peroxide (H_2_O_2_) and malondialdehyde (MDA) were determined following the methodology described by Mansinhos *et al*.^18^ Approximately 100 mg of fresh plant tissue was homogenized in 1 mL of 0.1% (w/v) trichloroacetic acid (TCA), followed by centrifugation. The resulting supernatant was used for both assays. For the H_2_O_2_ assay, 0.2 mL of supernatant was mixed with 0.2 mL of 10 mmol L^−1^ potassium phosphate buffer, and the absorbance was recorded (390 nm) after 30 min using a microplate reader (Synergy HTX MultiMode Microplate Reader, BioTek Instruments, Inc., Winooski, VT, USA). The results were expressed as micromoles of H_2_O_2_ equivalents per gram of fresh weight (μmol_H2O2_ g_FW_^−1^).

Lipid peroxidation was evaluated by determining the amount of MDA available to react with 2‐thiobarbituric acid (TBA). For that, 0.5 mL of the supernatant was mixed with 0.5 mL of either positive (TCA (5%, w/v) + TBA (20%, w/v)) or negative control solutions (TCA (20%, w/v)). Samples were incubated at 95 °C for 30 min and then cooled on ice to stop the reaction. After 5 min, the absorbance was measured at different wavelengths (600, 532, and 440 nm), and the results were expressed as nanomoles of MDA equivalents per gram of fresh weight (nmol_MDA_ g_FW_^−1^).

### Osmoprotectants

Soluble sugar content was determined using the phenol–sulfuric acid method, as described by Abid *et al*.^19^ Plant material (25 mg) was macerated in 80% (v/v) ethanol (2 mL), and the samples were kept in a water bath (80 °C) for 30 min. Subsequently, 100 μL of the supernatant was diluted with an equal volume of distilled water. 200 μL phenol (9%, w/v) and 1 mL sulfuric acid were added to the reaction, and after 30 min incubation the absorbance was read (490 nm) using the microplate reader. The results were expressed as milligrams of glucose equivalents per gram of fresh weight (mg_GLU_ g_FW_^−1^).

Proline was determined following the method described by Martins *et al*.^20^ 25 g of plant material was subjected to triple extraction (at 80 °C for 30 min) with 0.5 mL of 80% (v/v) ethanol. The extract was then incubated for 1 h at 100 °C with 1% (w/v) ninhydrin reagent previously prepared in 60% (v/v) acetic acid. Subsequently, 1 mL toluene was added. The absorbance of the organic phase was measured (520 nm), and the results were expressed as micromoles of proline equivalents per gram of fresh weight (μmol_PRO_ g_FW_^−1^).

### Phenolic compounds: extraction, profile, and antioxidant activity

#### Green extraction using NADES‐UAE combination

The plant material was oven dried at 40 °C and ground to a particle size of <2 mm. The phenolic compounds were extracted using NADES, composed of choline chloride–lactic acid (1:2) with 30% (w/w) distilled water, given the advantages of these eco‐friendly solvents for the extraction of phenolics from Lamiaceae plants.^7^ This green solvent was selected based on its proven effectiveness in extracting phenolics from Lamiaceae species, as reported in previous studies.^7^, ^18^, ^21^ A plant‐to‐solvent ratio of 2.5:100 (w/v) was used, and the extraction was carried out via UAE for 30 min at 50 °C. The resulting extracts were filtered and stored at −20 °C until further analysis.

#### Phenolic profile by high‐performance liquid chromatography coupled with photodiode array detection of green extracts

The main families of polyphenols present in *Salvia rosmarinus* green extracts were analyzed using high‐performance liquid chromatography coupled with photodiode array detection (HPLC‐PAD). The identified flavonoids included luteolin‐7‐*O*‐glucuronide, confirmed with a reference standard, as well as other tentative compounds that exhibited UV absorption maxima around 360 nm and spectral profiles similar to those of apigenin and luteolin glycosides. In addition, hydroxycinnamic acids such as caffeic acid and RA were detected at 320 nm, along with tentative cinnamic acid derivatives. The flavanone hesperidin and the phenolic diterpenoids carnosol and carnosic acid were identified at 280 nm based on their characteristic absorption spectra. A 20 μL aliquot of each extract, previously diluted up to four times and filtered through a 0.20 μm membrane, was injected into a Waters HPLC system equipped with a 1525 pump and PDA 996 detector (Waters Corp., Milford, MA, USA). The column used for the chromatographic separation was a 250 mm × 4.6 mm Kinetex C18 (Phenomenex, Torrance, CA, USA). The mobile phase consisted of Milli‐Q water containing 5% (v/v) formic acid (solution A) and HPLC‐grade methanol (solution B). The flow rate was set to 1 mL min^−1^. The gradient program started with 20% solution B, increasing to 35% at 30 min, 40% B at 45 min, 50% B at 50 min, 60% at 55 min, and reaching 95% of B at 60 min, maintained for an additional 3 min. Compounds were identified or tentatively identified based on retention time and UV absorption spectra, in comparison with those of standards. The concentrations of the analytes were determined using external standard calibration. Supporting Information, Table S1, sums up the retention time (RT), detection wavelength (nm), equation of calibration curve, *R* ^2^, limits of detection (LOD) and quantification (LOQ), and range (mg L^−1^) of the compounds. LOD and LOQ ranged from 0.002 to 0.22 mg L^−1^ and from 0.005 to 0.74 mg L^−1^, respectively.

#### Antioxidant capacity of green extracts

The antioxidant capacity of the green extracts was evaluated following the methodology described by Mansinhos *et al*.^7^ Four different methods were used: ferric reducing antioxidant power (FRAP), based on a single‐electron transfer‐based assay; oxygen radical absorbance capacity (ORAC), an atom hydrogen transfer‐based assay, and 2,2‐azino‐bis(3‐ethylbenzothiazoline‐6‐sulfonic acid (ABTS) and 2,2‐diphenyl‐1‐ picrylhydrazyl (DPPH), which involve both mechanisms. The results were expressed as milligrams of ascorbic acid (FRAP) or Trolox (ABTS, DPPH, ORAC) equivalents per gram of dry weight (mg_AAE_ g_DW_^−1^ or mg_TE_ g_DW_^−1^).

### Isolation and chemical composition of essential oil

Dried plant material of *Salvia rosmarinus* (~50 g) from each treatment was subjected to hydrodistillation for 3 h using distilled water in a Clevenger‐type apparatus. The obtained EO was dried over anhydrous sodium, transferred to a vial, and stored in a sealed tube at 4 °C until chemical analysis.

The EO profile was assessed according to Soliman *et al*.'s^22^ procedure. Samples were diluted in hexane at a ratio of 1:19 (v/v). Analysis was performed using a 7890B GC system equipped with a 5977A mass selective detector (Agilent Technologies, Santa Clara, CA, USA) and an HP‐5MS capillary column (30 m × 0.25 mm i.d., 0.25 μm film thickness). Helium was employed as the carrier gas at a constant flow rate of 1.0 mL min^−1^, with a split injection mode set at 1:20. A 1 μL aliquot of each sample was injected. The oven temperature was programmed as follows: initial temperature of 40 °C held for 1 min, ramped at 4 °C min^−1^ to 150 °C and held for 6 min, then increased at 4 °C min^−1^ to 210 °C and held for 1 min. The injector and detector temperatures were maintained at 280 and 230 °C, respectively. Mass spectra were acquired using electron ionization (EI) at 70 eV, over a mass range of *m*/*z* 50–550, with a solvent delay of 4 min. Compound identification was based on the comparison of mass spectral fragmentation patterns with the spectral data obtained from Mass Spectral Library Version 2.0 g (NIST‐MS, 2012, Agilent Technologies), along with consideration of the calculated retention indices.

### Statistical analysis

The present data are expressed as mean ± standard error (SE) and evaluated using one‐way analysis of variance (ANOVA) along with Duncan's new multiple range test (*P* < 0.05; *n* = 3). Multivariate data analysis, including principal component analysis (PCA) and heatmap, was performed using OriginPro software, version 2024 (OriginLab Corp., Northampton, MA, USA).

## RESULTS AND DISCUSSION

### Biochemical responses

**Figure 1:**
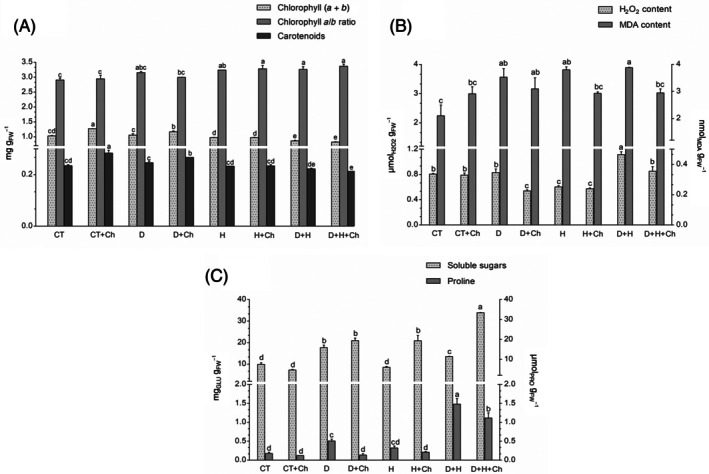
Effect of drought and heat stresses, their combination, and the application of chitosan on the contents of (A) total chlorophyll, chlorophyll *a*/*b* ratio and carotenoids, (B) oxidative stress markers, and (C) osmoprotectant in *Salvia rosmarinus*. The results were analyzed using one‐way analysis of variance (ANOVA) followed by Duncan's new multiple range test. Different letters indicate significant differences (*P* < 0.05) among different treatments. CT, control; Ch, chitosan; D, drought; H, heat.

Abiotic stresses such as drought and heat are known to disrupt chlorophyll biosynthesis, leading to a reduction in photosynthetic efficiency.^23^ In the present study, total chlorophyll and carotenoid contents in rosemary were not significantly affected (*P* ≤ 0.05) by individual stress treatments (drought or heat). However, a significant 16% reduction in total chlorophyll was observed under combined drought–heat stress (Fig. 1(A)). As a stress‐resilient Mediterranean species, *Salvia rosmarinus* possesses morphological adaptations, such as small, leathery leaves with thick cuticles and glandular trichomes, that reduce water loss and enhance stress tolerance.^24^, ^25^ The chlorophyll *a*/*b* ratio is a well‐established indicator of adjustments in the photosynthetic apparatus in response to varying light and stress conditions, as it reflects the relative size of the PSII light‐harvesting antenna and the proportion of accessory pigments. An increase in this ratio usually indicates a reduction in chlorophyll *b*, suggesting antenna contraction to minimize photodamage when energy utilization is limited.^26^ Compared to the control, the chlorophyll *a*/*b* ratio increased under all stress treatments, but the change was significant only under heat treatments (both individual and combined), which is consistent with a relative decrease in chlorophyll *b* (Fig. 1(A); Supporting Information, Fig. S1). This response suggests that rosemary adjusts its photosynthetic machinery in response to stress by reducing the size of the PSII antenna, particularly under combined drought–heat stress, where the highest ratio (3.26) was observed. This strategy helps mitigate excess light absorption and potential oxidative damage. Similar patterns have been reported in *Arachis hypogaea* L. under salt and osmotic stress.^26^

Foliar application of chitosan notably enhanced pigment retention under CT (+24% chlorophyll and +21% carotenoids) and drought conditions (+11% chlorophyll and +8% carotenoids), although no significant improvement was observed under heat stress. These findings align with previous reports showing that chitosan alleviates drought‐induced pigment degradation in several species.^12^, ^14^, ^15^, ^16^, ^23^ Chitosan also influenced the chlorophyll *a*/*b* ratio. In the drought + chitosan treatment, the ratio decreased by 5.1% compared to drought alone. In heat + chitosan and heat + drought + chitosan treatments, the ratios increased by 1.3% and 3.3%, respectively, compared to the respective stresses. The highest ratio (3.37) was observed under combined stress with chitosan (Supporting Information, Fig. S2). These results suggest that the effects of chitosan are stress dependent, attenuating antenna contraction under drought but slightly enhancing it under heat, and even more so under combined stress.

Chlorophyll degradation under stress is commonly linked to reactive oxygen species (ROS) overproduction and damage to cellular membranes (e.g., thylakoid membranes).^18^ Consistently, the highest levels of H_2_O_2_ and MDA were detected under combined drought–heat stress (Fig. 1(B)), indicating synergistic stress effects. MDA accumulation increased by 59%, 70%, and 73% under drought, heat, and combined conditions, respectively. These results contrast with previous findings that reported MDA reduction under combined stress in rosemary,^24^ suggesting variable antioxidant dynamics. Interestingly, high MDA levels were observed even under conditions where H _2_ O _2_ levels remained low and pigment degradation was not apparent. This may reflect a transient oxidative burst, countered by activation of antioxidant defenses.^27^ Carotenoids, as non‐enzymatic antioxidants, mitigate ROS damage by quenching singlet oxygen and dissipating excess energy.^28^ Chitosan treatment significantly reduced both H_2_O_2_ and MDA levels under heat and drought combined stress, restoring oxidative parameters to control levels (Fig. 1(B)), in agreement with previous findings in *Catharanthus roseus* ^23^ and thyme.^29^ In response to abiotic stress, plants commonly accumulate osmoprotectants such as proline and soluble sugars to maintain cellular homeostasis. In the present study, proline content increased significantly under drought and combined drought–heat stress, with the latter inducing a dramatic 744% rise relative to the control (Fig. 1(C)). This pronounced accumulation highlights the role of proline as a key osmolyte in rosemary, facilitating osmotic adjustment and maintaining cell turgor under water deficit conditions. These findings align with previous reports on *Salvia rosmarinus* subjected to drought stress,^30^, ^31^ supporting the hypothesis that proline accumulation constitutes an adaptive mechanism enhancing abiotic stress tolerance. Comparable trends have been documented in various other species exposed to drought, including *Mentha piperita* L., *Lallemantia iberica* (M. Bieb.) Fisch. & C.A. Mey., *Lavandula dentata* L., and *Solanum lycopersicum* L.^12^, ^13^, ^15^, ^32^ In *Salvia abrotanoides*, Dowom *et al*.^16^ observed that chitosan‐induced proline accumulation under drought stress was dose dependent: higher concentrations (60 and 90 ppm) significantly elevated proline levels, whereas a lower dose (30 ppm) had no notable effect. Similarly, in *Salvia rosmarinus*, chitosan application under non‐stress conditions did not significantly affect proline content, mirroring trends seen in *Oryza sativa* L.,^33^ *Catharanthus roseus*,^23^ *Salvia abrotanoides*,^16^ and *Lallemantia iberica*.^15^

Soluble sugars play a crucial role in plant metabolism, contributing to processes such as photosynthesis, osmotic adjustment, and the maintenance of cellular homeostasis. Under abiotic stress, sugars are not only metabolic intermediates but also act as signaling molecules that regulate stress perception and response pathways, including the modulation of ROS and osmotic balance.^34^ In this study, both drought treatments significantly increased soluble sugar levels in rosemary, with the most pronounced accumulation observed under individual drought stress (+78% compared to the control). These findings are consistent with reports in other Lamiaceae species subjected to drought,^12^, ^16^, ^29^, ^30^, ^35^ including previous work in rosemary itself.^30^ In contrast, heat stress alone did not significantly affect soluble sugar content compared to control plants. Transcriptome evidence indicates that genes involved in carbohydrate metabolism are typically upregulated by chitosan under drought stress conditions.^13^ Interestingly, in rosemary, this transcriptional activation was more evident under heat stress than drought. Chitosan application markedly enhanced sugar accumulation under both individual and combined stress conditions, particularly under combined drought–heat stress, where sugar content increased by 150% relative to untreated combined stress and 242% compared to the control. No significant changes in sugar levels were observed under chitosan treatment in non‐stress conditions, consistent with results reported in *Lavandula dentata* ^12^ and *Thymus daenensis* Celak,^29^ but in contrast to findings in *Salvia abrotanoides*.^16^ These findings highlight the species‐specific nature of chitosan responses and emphasize the importance of tailoring biostimulant strategies for the sustainable cultivation of medicinal and aromatic plants under climate‐related stress conditions.

### Secondary metabolism modulation

#### Phenolic compounds

Phenolic compounds play a crucial role in plant defense against abiotic stress, primarily through their ability to scavenge ROS and mitigate oxidative damage. A growing body of evidence has demonstrated that abiotic stressors, including drought, extreme temperatures, nutrient limitation, salinity, UV‐B radiation, and heavy metals exposure, can modulate the biosynthesis and accumulation of phenolic compounds, thereby influencing their biological activity and ecological functions.^2^, ^36^

In this study, the effects of drought, heat, and their combination on the phenolic profile of rosemary were examined, along with the influence of foliar‐applied chitosan under these stress conditions. Representative HPLC chromatograms for each treatment are provided in Supporting Information, Fig. S3. A total of 18 phenolic compounds were detected and quantified, of which seven were unequivocally identified. These included two phenolic acids (caffeic acid and RA), two phenolic diterpenes (carnosol and carnosic acid), and three flavonoids (hesperidin, luteolin‐7‐glucuronide, and luteolin‐3′‐glucuronide). The remaining 11 compounds, although not fully characterized, were tentatively identified as flavonoids, mainly derivatives of luteolin and apigenin, and two phenolic acids likely belonging to the cinnamic acid class (Supporting Information, Table S2).

**Figure 2:**
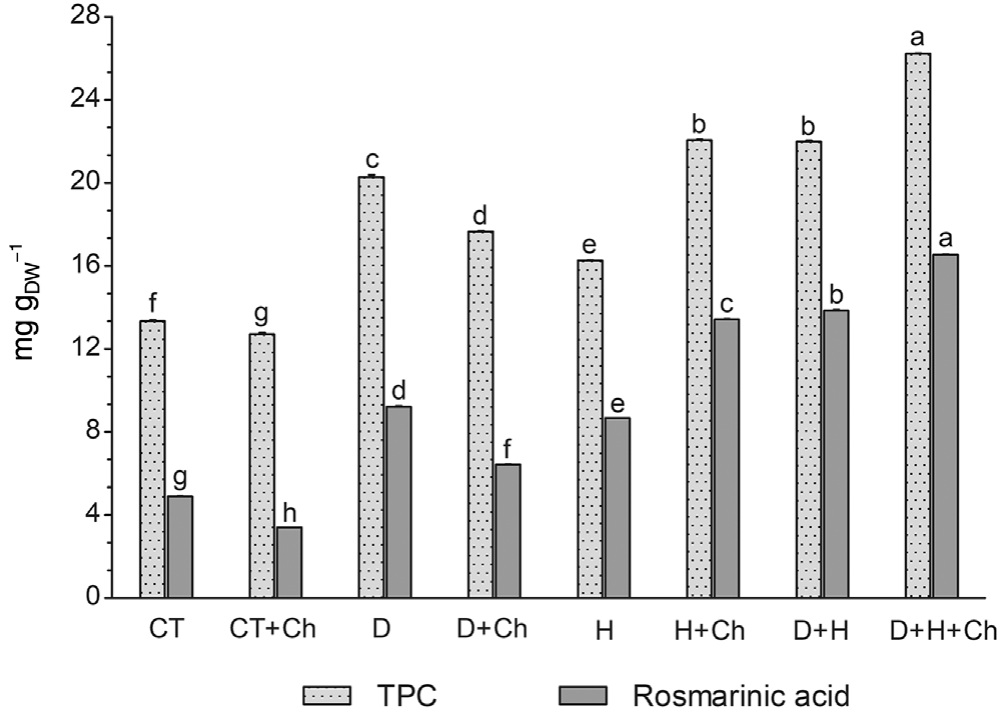
Effect of drought and heat stresses, their combination, and the application of chitosan on total phenolic and rosmarinic acid contents of *Salvia rosmarinus* extracts. The results were analyzed using one‐way analysis of variance (ANOVA) followed by Duncan's new multiple range test. Different letters indicate significant differences (*P* < 0.05) among different treatments. CT, control; Ch, chitosan; D, drought; H, heat.

The total phenolic content (TPC) in rosemary ranged from 12.71 to 26.24 mg g_DW_^−1^ (Fig. 2 and Supporting Information, Table S2). Statistical analysis showed that all abiotic stress treatments, whether applied individually or in combination, significantly increased TPC compared to control plants. Among the chitosan‐untreated groups, the highest TPC was observed under combined drought–heat stress (22.00 ± 0.04 mg g_DW_^−1^), followed by drought (20.29 ± 0.11 mg g_DW_^−1^) and heat stress (16.27 ± 0.02 mg g_DW_^−1^). These findings are consistent with previous studies in other *Salvia* species, including *Salvia officinalis* L.,^37^ *Salvia sinaloensis* Fern.,^38^ *Salvia abrotanoides*,^16^ and *Salvia nemorosa* L.,^35^ which reported that water deficit enhances TPC. Chitosan foliar application significantly increased TPC, but only under heat‐related conditions: both heat alone and combined drought–heat stress. The highest TPC overall was recorded under combined stress with chitosan treatment (26.24 ± 0.01 mg g_DW_^−1^), corresponding to a 97% increase relative to the control. Under non‐stress conditions, chitosan slightly reduced TPC by 5%. These results contrast with findings in *Salvia abrotanoides*,^16^ *Salvia officinalis*,^37^ and *Lavandula dentata*,^12^ where chitosan application increased TPC regardless of water availability.

In this study, phenolic compounds were extracted from *Salvia rosmarinus* using a NADES composed of choline chloride–lactic acid in a 1:2 molar ratio. RA was identified as the predominant phenolic compound in the extracts, with concentrations ranging from 3.41 ± 0.01 to 16.56 ± 0.02 mg g_DW_^−1^ (Fig. 2; Supporting Information, Table S2). Under control conditions, the RA content was approximately 42% higher than that reported by Jacotet‐Navarro *et al*.,^39^ who employed a conventional organic solvent for extraction. This finding underscores the superior extraction efficiency of the NADES system for recovering this bioactive compound. Vieira *et al*.^40^ reported lower RA yields (1.00 ± 0.12 mg g^−1^) using alternative NADES formulations such as lactic acid–glucose (5:1)/menthol–lauric acid (2:1), corresponding to less than 80% of the RA content obtained under control conditions in our study (4.90 ± 0.01 mg g^−1^). Similar extraction efficiencies were observed by Jurić *et al*.,^41^ who quantified RA in rosemary extracts ranging from 10.27 ± 0.73 to 15.69 ± 0.73 mg g^−1^, using choline chloride‐based NADES (1:1 molar ratio) with various HBD, including fructose, citric acid, 2‐propanediol, and urea. Complementary insights were provided by Chisha *et al*.,^8^ who employed molecular simulation tools, including the conductor‐like screening model for segment activity (COSMO‐SAC) coefficient and molecular dynamics, to compare the efficacy of various solvents in RA extraction. Their study identified choline chloride–lactic acid (1:2) as the most effective solvent, outperforming not only ethanol but also other NADES such as choline chloride–citric acid (2:1) and choline chloride–glycerol (1:2).

In the present study, all abiotic stress treatments significantly enhanced the accumulation of RA, with increases ranging from 77% to 183% compared to control plants. The highest RA concentration (13.86 ± 0.02 mg g DW ^−1^) was observed under combined drought–heat stress (Fig. 2; Supporting Information, Table S2). These findings are consistent with previous reports demonstrating drought‐induced stimulation of RA biosynthesis in *Thymus vulgaris* L.^42^ and *Thymus daenensis*.^43^ However, contrasting responses have been documented in other Lamiaceae species, such as *Melissa officinalis* L.^44^ and *Thymus lotocephalus* G. López & R. Morales,^21^ highlighting species‐specific variability. The impact of heat stress on RA biosynthesis also appears to be species dependent. While increased RA levels have been reported in *Lavandula viridis* L'Hér and *Thymus lotocephalus*,^18^ reductions were observed in *Mentha spicata* L.^45^ In the current study, foliar application of chitosan under non‐stress conditions led to a 30% reduction in RA content (Fig. 2; Table S2). This result contrasts with several reports showing that higher chitosan concentrations promote RA accumulation in other species, including *Melissa officinalis* (50–100 mg L^−1^),^46^ *Dracocephalum kotschyi* Boiss (100–400 mg L^−1^)^47^ *Salvia yangii* B.T. Drew (100–200 mg L^−1^),^48^ and *Salvia abrotanoides* (100–200 mg L^−1^).^48^ Under abiotic stress conditions, chitosan had differential effects on RA accumulation. In heat‐stressed rosemary, chitosan treatment led to a 55% increase in RA content, and under combined stress (heat + drought) a 20% increase was observed relative to the corresponding untreated plants. Conversely, under drought stress alone, chitosan application reduced RA levels by 30%, mirroring findings reported in *Salvia abrotanoides* and *Salvia yangii*.^48^ Notably, the highest RA concentration (16.56 ± 0.02 mg g_DW_^−1^) was observed in plants subjected to combined drought–heat stress with chitosan treatment, corresponding to a 238% increase relative to the control (Fig. 2; Table S2). These results underscore the complex interaction between stress type and chitosan application, and the importance of species‐specific responses in modulating phenolic metabolism.

**Figure 3:**
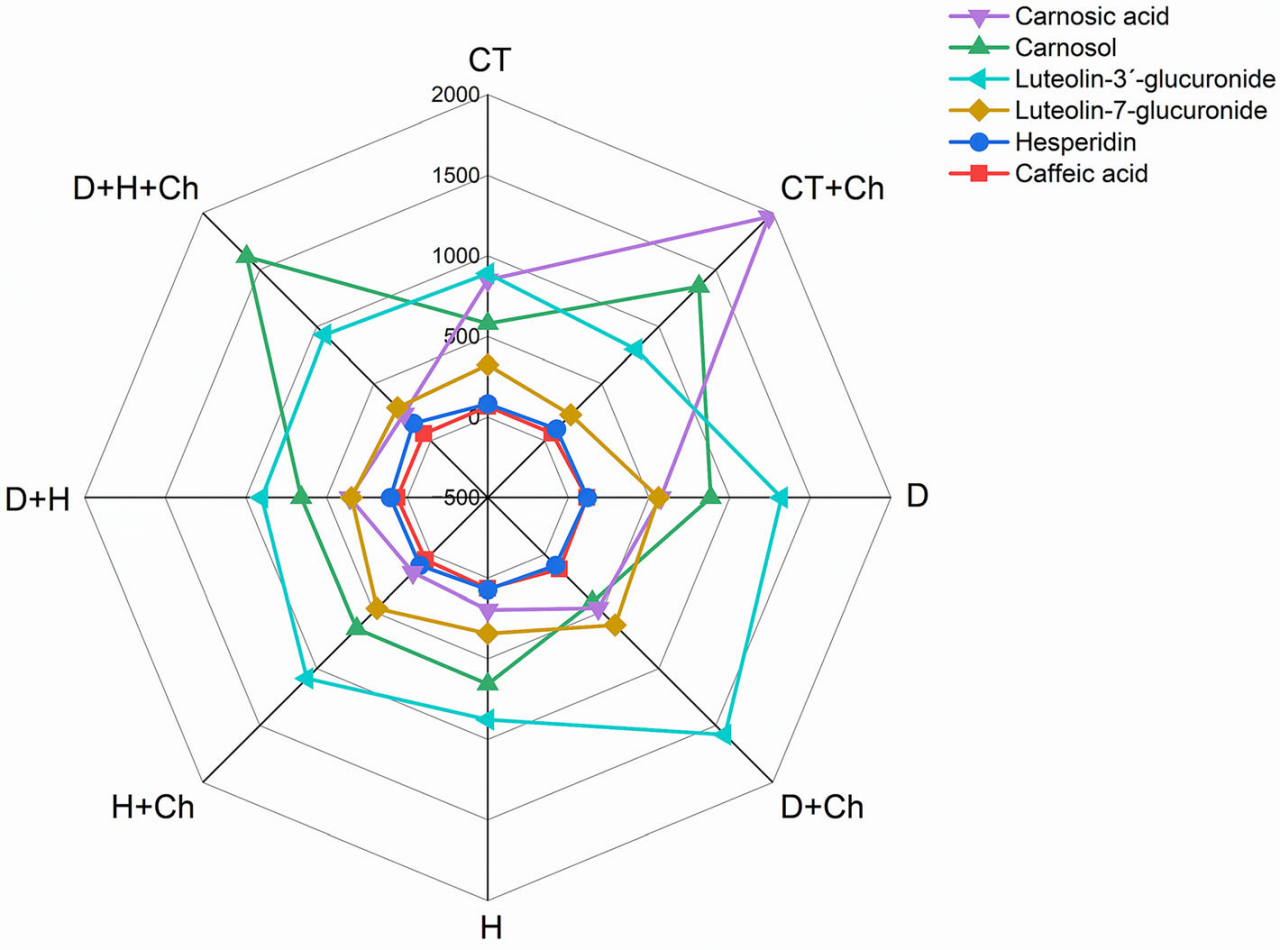
Radar charts (μg g_DW_^−1^) illustrating the effects of drought and heat stresses, their interaction, and the application of chitosan on the levels of other phenolic compounds identified by HPLC‐PAD in *Salvia rosmarinus*. CT, control; Ch, chitosan; D, drought; H, heat.

Carnosol and carnosic acid are two major bioactive compounds in rosemary. In the present study, foliar application of chitosan significantly promoted the carnosol accumulation, increasing its content by 134% under control conditions and by 179% under combined drought–heat stress. The highest concentration of carnosol (1.61 ± 0.00 mg g^−1^) was recorded under combined stress (Fig. 3; Supporting Information, Table S2). Interestingly, both individual drought and heat stress treatments independently stimulated carnosol biosynthesis. However, under these conditions, chitosan did not further enhance its accumulation, suggesting a potential threshold or saturation effect in the regulation of this metabolite under stress. In contrast, carnosic acid content consistently declined under all abiotic stress conditions, irrespective of chitosan treatment, with reductions ranging from 33% to 82%. However, under non‐stress conditions, chitosan elicitation markedly increased carnosic acid accumulation by 131%. The observed decline in carnosic acid, alongside a concomitant rise in carnosol levels under stress, supports the hypothesis that carnosic acid is oxidized to carnosol as part of the plant's antioxidant defense mechanism. Similar findings were reported by Loussouarn *et al*.,^49^ who observed increased carnosol levels following combined heat and light stress in rosemary. Recent transcriptomic analyses by Lai *et al*.^50^ further corroborate these findings, revealing that drought and heat stresses upregulate genes involved in the biosynthetic pathways of carnosic acid and RA. In particular, genes encoding ferruginol synthase and ferruginol monooxygenase – key enzymes in the abietane‐type diterpenes biosynthetic pathway – were significantly upregulated under drought stress, implicating these metabolites in drought tolerance. Conversely, cytochrome P450 enzymes, especially CYP98A family members, showed heightened expression in response to heat stress. Additionally, RA synthase expression was elevated under both stress conditions, suggesting that enhanced RA biosynthesis is part of a general adaptive response to abiotic stress.

Luteolin‐3′‐glucuronide was identified as one of the predominant flavonoids in rosemary extracts, exhibiting its highest accumulation under drought stress conditions. Notably, drought combined with chitosan application resulted in a 78% increase in luteolin‐3′‐glucuronide content compared to the control (Fig. 3; Supporting Information, Table S2). This finding aligns with recent work by Truzzi *et al*.,^51^ who also detected a luteolin derivative in rosemary extracts obtained using NADES. Additionally, Rafya *et al*.,^52^ employing ultra‐high‐performance liquid chromatography–diode array detection–electrospray ionization–mass spectrometry, identified luteolin‐3′‐glucuronide exclusively in the aqueous residue of rosemary powder extracts, highlighting the compound's strong polarity and affinity for water‐rich phases. The high polarity of luteolin‐3′‐glucuronide is attributed to the presence of the glucuronic acid moiety, which introduces additional hydroxyl and carboxyl groups, thereby enhancing water solubility. This structural feature promotes its preferential partitioning into aqueous phases during extraction processes,^53^ emphasizing the critical role of solvent selection and extraction strategy in the efficient recovery of specific phenolic subclasses. In addition to luteolin‐3′‐glucuronide, luteolin‐7‐glucuronide levels also increased under drought stress in the present study, with further enhancement observed following chitosan application (Fig. 3; Table S2). Similar trends have been reported for luteolin‐7‐*O*‐β‐glucuronide accumulation in other plant species exposed to drought stress,^54^ suggesting a conserved stress‐induced flavonoid biosynthetic response.

#### Antioxidant activity of phenolic extracts

The antioxidant capacity of rosemary extracts increased significantly (*P* < 0.05) under abiotic stress. The most pronounced enhancement was observed under combined drought–heat stress, with increases of 39–102% relative to control conditions. However, an exception was noted in the DPPH scavenging activity, where heat stress alone induced the highest increase (+66% compared to the control) (Table 1). These findings are consistent with previous studies reporting increased antioxidant activity in rosemary^30^ and thyme^30^, ^42^ extracts under drought conditions. However, drought led to a reduction in antioxidant activity in *Salvia officinalis*.^37^ In *Salvia sinaloensis*, moderate drought enhanced antioxidant capacity, whereas severe stress resulted in an 80% decline^38^ Similarly, *Salvia dolomitica* Codd subjected to severe drought exhibited a notable reduction in antioxidant activity from days 4 to 11, followed by partial recovery by day 25.^55^ In contrast, heat stress has been reported to diminish total antioxidant capacity in spearmint.^45^

**Table 1:** Effect of drought and heat stresses, their combination, and the application of chitosan on the antioxidant activity of green extracts from *Salvia rosmarinus*, evaluated by the 2,2‐diphenyl‐1‐picrylhydrazyl (DPPH), 2,2′‐azino‐bis(3‐ethylbenzothiazoline‐6‐sulfonic acid) (ABTS), ferric reducing antioxidant power (FRAP), and oxygen radical absorbance capacity (ORAC) assays

	Treatment							
	CT	CT + Ch	D	D + Ch	H	H + Ch	D + H	D + H + Ch
DPPH	52.95 ± 1.43d	50.14 ± 0.22d	70.76 ± 2.57b	63.72 ± 3.03c	87.86 ± 2.71a	72.92 ± 1.05b	68.37 ± 1.24bc	72.18 ± 1.75b
ABTS	22.44 ± 1.30e	28.21 ± 0.92d	39.79 ± 1.24b	36.59 ± 0.81c	30.47 ± 1.19d	42.27 ± 0.23ab	45.26 ± 0.34a	44.97 ± 0.31a
FRAP	98.65 ± 2.23e	81.79 ± 1.52f	126.53 ± 1.29b	119.38 ± 0.70c	108.55 ± 1.00d	124.18 ± 1.76bc	131.09 ± 2.58ab	137.24 ± 3.69a
ORAC	150.04 ± 0.71e	123.75 ± 4.39f	181.56 ± 2.13c	172.42 ± 3.39cd	159.98 ± 2.47de	175.77 ± 1.64cd	238.58 ± 5.82a	201.16 ± 7.09b

The results are expressed as milligrams of ascorbic acid (FRAP) or Trolox (ABTS, DPPH, ORAC) equivalents per gram of dry weight (mg_AAE_ g_DW_^−1^ or mg_TE_ g_DW_^−1^) and were analyzed using one‐way analysis of variance (ANOVA) followed by Duncan's new multiple range test. Different letters indicate significant differences (*P* < 0.05) among different treatments.

Abbreviations: CT, control; Ch, chitosan; D, drought; H, heat.

Under non‐stress conditions, the application of chitosan did not significantly enhance antioxidant activity, except for a modest increase in ABTS radical scavenging. Similar findings were reported for lavender,^12^ basil,^56^ and lemon balm.^56^ Under stress conditions, however, chitosan was effective only under heat stress, significantly improving antioxidant activity in ABTS, DPPH, and FRAP assays. Consistent with these findings, chitosan had no impact on antioxidant activity in lavender under drought conditions,^12^ whereas it enhanced the antioxidant properties of two basil species subjected to drought.^57^ The differential efficacy of chitosan likely reflects the specificity of the stress signal and the physiological status of the plant. As an abiotic elicitor, chitosan can trigger antioxidant responses, but its effectiveness depends on the magnitude of ROS accumulation and the plant's baseline defense capacity. Under non‐stress conditions, ROS levels are low and the endogenous antioxidant pathways are only marginally active, so the chitosan signal may be insufficient to elicit an antioxidant response. Under combined stress, ROS accumulation becomes excessive (as shown in Fig. 1(B)), overwhelming the plant's defense mechanisms. In such scenarios, metabolic resources are redirected towards essential protective processes such as maintaining membrane stability and osmotic balance (evidenced by the increase in osmoprotectants in Fig. 1(C)), thereby limiting the plant's responsiveness of chitosan. Conversely, under heat stress alone, ROS levels rise moderately, remaining within a range that can activate antioxidant defenses. In this context, chitosan may act synergistically with heat‐induced signaling pathways, enhancing antioxidant capacity without exceeding the plant's metabolic limits.^58^

**Figure 4:**
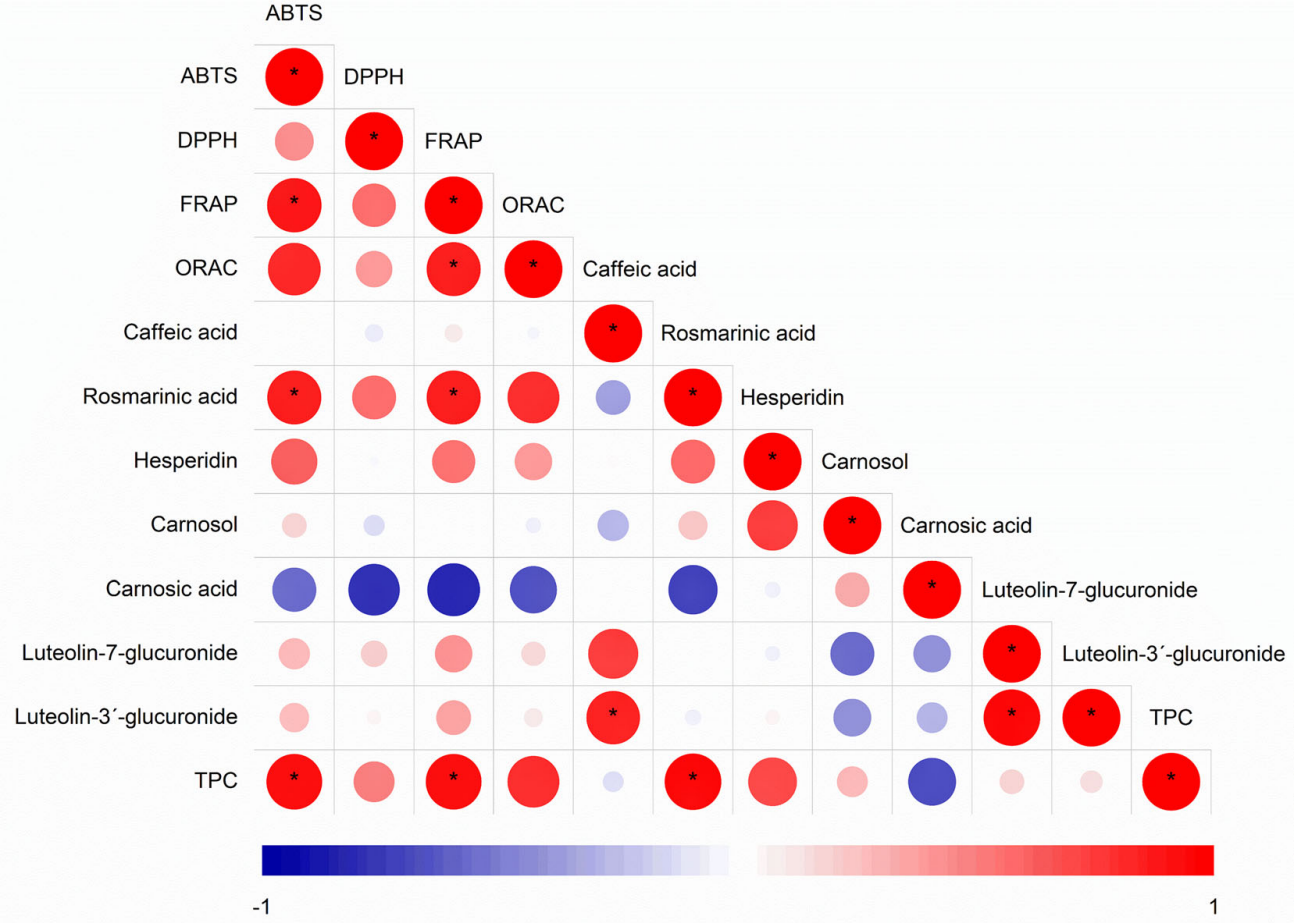
Heatmap corresponding to Pearson's correlation coefficients (circles) between total phenolic content (TPC), individual phenolic compounds identified by HPLC, and antioxidant activity (DPPH, ABTS, FRAP, ORAC) from *Salvia rosmarinus* exposed to drought and heat stresses, their interaction, and chitosan. Asterisks indicate statistically significant correlations (*P* ≤ 0.01).

A strong positive correlation was observed between the TPC and antioxidant activity, particularly FRAP and ABTS assays. This highlights the important role that phenolic compounds play in the antioxidant defense system of rosemary (Fig. 4). These results confirm that these compounds are important contributors to ROS neutralization under abiotic stress. Of the seven phenolic compounds identified, RA exhibited the highest antioxidant activity, followed by hesperidin, luteolin‐7‐glucuronide, and luteolin‐3′‐glucuronide, in descending order.

#### Essential oil profile

**Figure 5:**
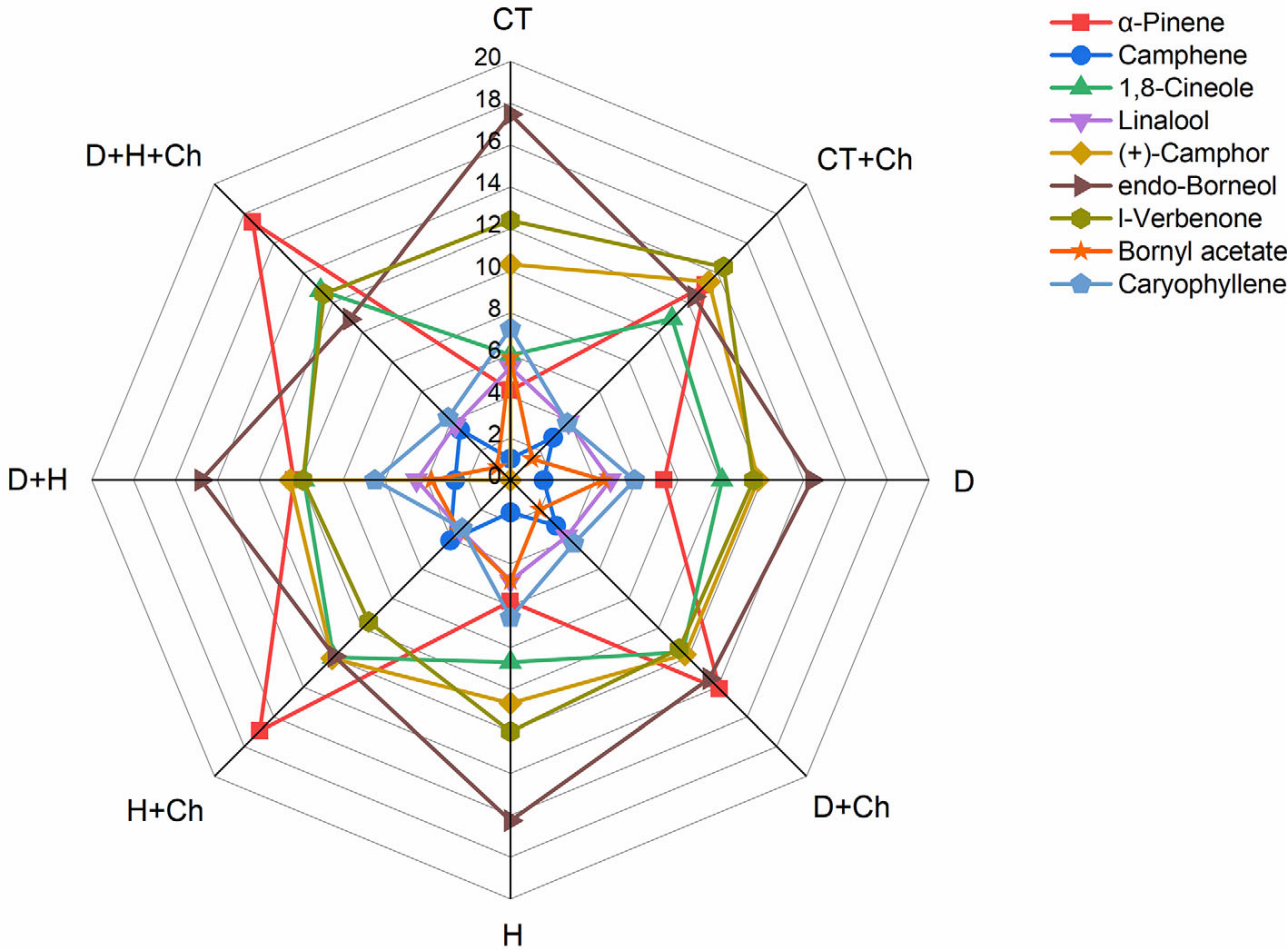
Radar charts (%) illustrating the effects of drought and heat stresses, their interaction, and the application of chitosan on the relative chemical composition (%) of the most abundant compounds in essential oils from *Salvia rosmarinus* determined by GC‐MS. CT, control; Ch, chitosan; D, drought; H, heat.

Rosemary EOs are widely recognized for their therapeutic, aromatic, and preservative properties. This study analyzed the chemical composition of *Salvia rosmarinus* EO obtained from control plants and those subjected to chitosan, drought, heat, or a combination of these factors (Fig. 5; Supporting Information, Table S3). Representative GC‐MS chromatograms for each treatment are provided in Supporting Information, Fig. S4. A total of 61 volatile compounds were identified. Significant quantitative differences in EO constituents were notably observed across treatments. Compared to the control plants, the abundance of 32%, 23%, and 21% of the identified compounds increased under drought, heat, and combined stress, respectively. Under non‐stress conditions, chitosan treatment promoted the biosynthesis of 28% of the identified compounds of EO. Under stress conditions, the stimulatory effect of chitosan was maintained, enhancing the production of 23% (drought), 26% (heat), and 38% (combined stress) of the compounds (Fig. 5; Table S3).

The EO profiles were dominated by monoterpenes, accounting for 79.75–91.71% of the total volatile fraction. Oxygenated monoterpenes were the most abundant subclass, accounting for 59.03–69.32% of the total composition. The main oxygenated monoterpenes identified were 1,8‐cineole (5.98–12.83%), linalool (3.38–5.38%), (+)‐camphor (10.31–14.59%), *endo*‐borneol (10.89–17.47%), l‐verbenone (9.57–14.41%), and bornyl acetate (0.89–5.84%) as the primary components of all rosemary EOs (Fig. 5; Supporting Information, Table S3), a profile consistent with previous reports.^59^, ^60^, ^61^, ^62^, ^63^, ^64^ Abiotic stress, both individually and in combination, resulted in a decrease in the levels of *endo*‐borneol (7–18%), bornyl acetate (17–35%), and linalool (11–17%) compared to the control group. Interestingly, chitosan treatment exacerbated the reduction of these compounds under both control and stress conditions. By contrast, drought stress specifically promoted the biosynthesis of (+)‐camphor, with the highest value observed under combined stress and chitosan application (14.59%). Although l‐verbenone was not responsive to individual or combined stress, it peaked in chitosan‐treated control plants (14.41%) (Fig. 5; Table S3). A similar increase in camphor was reported in *Lavandula viridis* under drought and heat stress.^65^ However, in *Salvia officinalis* ^37^ and *Lavandula dentata*,^12^ drought reduced borneol and camphor levels, respectively. All stress treatments, particularly drought, significantly stimulated the biosynthesis of 1,8‐cineole. The highest accumulation occurred in chitosan‐treated plants under combined stress (+30% than combined stress and +115% compared to the control). These findings align with previous studies indicating that moderate water deficit can enhance 1,8‐cineole production in various aromatic species.^37^, ^66^, ^67^, ^68^, ^69^, ^70^, ^71^, ^72^ However, other studies have reported a decline in 1,8‐cineole content in *Lavandula viridis*,^65^ *Salvia rosmarinus*,^10^ *Salvia reuterana*,^72^ and *Lavandula angustifolia* ^69^ under drought conditions. In *Lavandula viridis*, moderate heat stress did not affect 1,8‐cineole accumulation, whereas severe heat stress significantly increased it.^65^ Interestingly, in contrast to our results, Giglou *et al*.^32^ found that chitosan did not increase 1,8‐cineole levels in peppermint under drought conditions.

Monoterpene hydrocarbons, including α‐pinene (4.29–17.40%) and camphene (1.03–4.07%), are also key constituents of rosemary EOs, contributing to stress adaptation. All abiotic stress treatments, especially combined stress, increased α‐pinene production (+35–141%). Chitosan treatment produced the most significant effect, with increases of +207% (control) and +229–305% (stress treatments). A similar pattern was observed for camphene (Fig. 5; Supporting Information, Table S3).

These results corroborate findings by Abbaszadeh *et al*.,^3^ although other studies have reported opposite trends.^10^, ^68^ Laftouhi *et al*.^11^ also observed increased camphene production in rosemary under combined drought–heat stress. According to Nogués *et al*.,^68^ emissions of non‐oxygenated monoterpenes correlate positively with photosynthesis, while oxygenated monoterpene emissions show a negative correlation, suggesting distinct regulatory mechanisms. Non‐oxygenated compounds appear to rely on *de novo* synthesis via photosynthetic carbon, whereas oxygenated volatiles are more strongly influenced by volatilization from storage pools and temperature dynamics.

The sesquiterpene fraction, which made up 7.65–18.78% of the total EOs, mainly consisted of hydrocarbon‐containing compounds (6.41–15.07%) (Fig. 5; Supporting Information, Table S3). Caryophyllene (3.26–7.24%) was the dominant sesquiterpene, which is consistent with the findings of previous studies.^60^, ^64^ However, its content declined by 9–55% across all stress treatments and with chitosan application (Fig. 5; Table S3). Conversely, Al‐Ghamdi^73^ reported increased caryophyllene production in marjoram subjected to drought and treated with chitosan (50–500 ppm). Oxygenated sesquiterpenes were present in minor amounts (0.948–3.71%), with caryophyllene oxide being the main compound. The highest levels of caryophyllene oxide were observed in the control group (1.66 ± 0.14%) while application of chitosan led to marked reductions under all conditions. Similar decreases in caryophyllene oxide have been reported in drought‐stressed rosemary.^59^

While most of the EO constituents identified here have been reported previously, some rare components, such as 3,9‐epoxy‐*p*‐mentha‐1,8(10)‐diene and (−)‐myrtenol, were only detected under specific stress conditions or in response to elicitation. Despite their low abundance, their presence suggests that particular environmental stimuli or elicitor treatments can activate otherwise latent metabolic pathways, thereby contributing to the chemical diversification of rosemary EOs under stress.

### PCA

**Figure 6:**
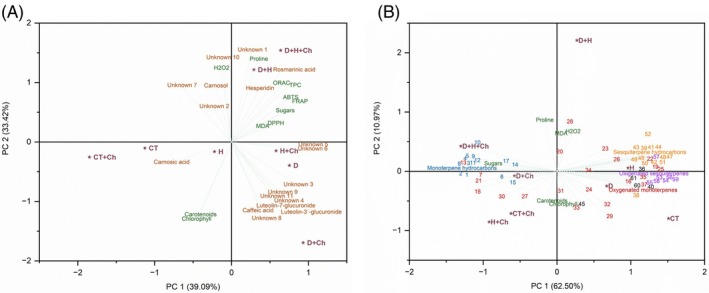
Principal component analysis (PCA) of *Salvia rosmarinus* exposed to drought, heat, chitosan, and their combination. (A) PCA based on stress‐related physiological and biochemical parameters, including photosynthetic pigments, osmoprotectants, oxidative stress markers, phenolic compounds (Supporting Information, Table S2), and associated antioxidant activity of green plant extracts. (B) PCA based on the same stress‐related parameters, combined with volatile compounds (Supporting Information, Table S3) identified in essential oils obtained by hydrodistillation. CT, control; Ch, chitosan; D, drought; H, heat.

Two separate PCAs were conducted to evaluate the impact of chitosan elicitation under drought and heat conditions, as well as the combined application of these two factors, on the accumulation of photosynthetic pigments, osmoprotectants, oxidative stress markers, and secondary metabolites in rosemary (Fig. 6). As phenolics and volatiles compounds are derived from different biosynthetic pathways and are regulated differently under stress conditions, two PCAs were performed: one for phenolics (Fig. 6(A)) and one for volatiles (Fig. 6(B)). This approach enhances interpretive clarity, enabling more precise identification of correlations between metabolite groups, biochemical parameters, and treatment conditions. Furthermore, this dual PCA reduces statistical noise and enables the identification of biologically meaningful patterns that could be obscured in a higher‐dimensional combined analysis.

The first PCA (Fig. 6(A)), based on phenolic compounds, explained 72.51% of the total variance through its first two principal components (PC1 and PC2). The biplot revealed clear discrimination among the treatment groups. Samples subjected to combined stress (with or without chitosan) clustered in the first quadrant, indicating a strong synergistic effect of dual stress on phenolic metabolism. This cluster was associated with increased levels of RA, hesperidin, and compounds 1 and 10, as well as higher total phenolic content (TPC) and antioxidant capacity (ORAC, ABTS, FRAP). This reflects an intensified secondary metabolism and enhanced antioxidant response. Chitosan was not the primary driver of this pattern, suggesting that dual stress conditions had a dominant influence on phenolic accumulation. By contrast, the control samples, clustered in the third quadrant, indicated a basal metabolic profile representative of unstressed plants. These samples were associated with lower levels of stress‐related metabolites but higher levels of carnosic acid. This pattern indicates that diterpenes such as carnosic acid accumulate preferentially under optimal conditions and potentially serve a constitutive protective role. Samples exposed to individual stresses were included in the fourth quadrant, with chitosan treatment resulting in distinct separation. These samples were associated with elevated levels of luteolin‐3′‐glucuronide, luteolin‐7‐glucuronide, caffeic acid, and several unidentified phenolic compounds (3, 5, 6, 8, 9, and 11), indicating that individual stressors activate specific branches of the phenylpropanoid pathway selectively, with chitosan modulating this response.

The second PCA (Fig. 6(B)), which focused on volatile compounds, accounted for 73.47% of the total variance (62.50% by PC1 and 10.97% by PC2). This analysis also revealed a clear separation dependent on treatment with PC1, effectively distinguishing between chitosan‐treated and untreated samples: the former clustered on the negative side of PC1, while the latter occupied the positive side. This separation suggests a consistent modulatory effect of chitosan on the volatile profile, likely via the activation of terpenoid biosynthesis and stress mitigation mechanisms. Within this PCA, a clear distinction was also observed between combined and individual stress treatments. The D + H + Ch samples located in the second quadrant were characterized by elevated levels of monoterpene hydrocarbons and specific oxygenated monoterpenes (compounds 13, 18, 7, and 21). Conversely, samples from individual stress treatments with chitosan (D + Ch and H + Ch) and chitosan alone (CT + Ch) clustered in the third quadrant. Samples not treated with chitosan, particularly those exposed to individual stress or serving as untreated controls, accumulated higher levels of sesquiterpene hydrocarbons, oxygenated sesquiterpenes, and a distinct set of monoterpenes. This suggests that, in the absence of elicitation, different branches of the volatile biosynthetic pathway are preferentially activated. Additionally, the greatest variability was exhibited by untreated samples exposed to combined stress. In the PCA score plot, the combined stress treatment (D + H) was clearly separated from the individual stresses (D and H) and CT, and from the chitosan‐treated samples, indicating a distinct metabolic response. This separation was primarily driven by elevated levels of compound 28 (3,9‐epoxy‐*p*‐mentha‐1,8(10)‐diene), coupled with increased accumulation of proline, MDA, and H_2_O_2_, which are well‐established markers of oxidative stress.

In both PCAs, oxidative stress markers (H_2_O_2_ and MDA) were strongly associated with D + H treatment, confirming an increased oxidative burden under combined abiotic stress. As expected, given the stress‐induced degradation of photosynthetic pigments, chlorophyll and carotenoid levels showed a negative correlation with these markers. Interestingly, drought stress combined with chitosan treatment was associated with higher levels of both chlorophyll and carotenoids, suggesting enhanced photoprotective and potentially physiological adjustments primed by chitosan. The position of the CT + Ch sample also supports this hypothesis, indicating that chitosan may stimulate pigment biosynthesis and confer protective or growth‐promoting effects even in the absence of external stress. The osmoprotectants proline and soluble sugars were closely associated with the D + H + Ch treatment, highlighting their contribution to osmotic adjustment under severe stress. These results also suggest that chitosan enhances the accumulation of osmoprotectants under stressful conditions, thereby reinforcing its role as a biostimulant that can prime multiple layers of plant defense and adaptive physiology.

## CONCLUSIONS

This study shows that foliar‐applied chitosan effectively mitigates drought and heat stresses in *Salvia rosmarinus*, particularly when both stresses occur simultaneously. It promotes osmoprotectant accumulation and enhances antioxidant activity of phenolic compounds. Multivariate analyses revealed distinct metabolite profiles under different stress conditions, with chitosan‐treated plants under combined stress showing elevated RA, 1,8‐cineole, camphor, and monoterpene hydrocarbons. In contrast, sesquiterpene and most of oxygenated terpene biosynthesis were less responsive. These results suggest that chitosan selectively boosts key bioactive compounds, improving stress resilience and phytochemical quality. As a sustainable strategy, chitosan holds promise for enhancing *Salvia rosmarinus* performance under abiotic stress, though further omics‐based studies are needed to clarify its mechanisms and optimize its application in medicinal plant cultivation.

## CONFLICT OF INTEREST

The authors declare that they have no known competing financial interests or personal relationships that could have appeared to influence the work reported in this article.

## Supporting information


**Figure S1.** Chlorophyll *a* and *b* of *Salvia rosmarinus* samples under drought and heat stresses, their combination, and the application of chitosan. The results were analyzed using one‐way analysis of variance (ANOVA) followed by Duncan's new multiple range test. Different letters mean significant differences (*P* < 0.05) among different treatments. CT, control; Ch, chitosan; D, drought; H, heat.
**Figure S2.** Effect of chitosan on chlorophyll *a*/*b* ratio of *Salvia rosmarinus* samples under drought and heat stresses, and their combination. Percentages indicate relative change compared to baseline. CT, control; Ch, chitosan; D, drought; H, heat.
**Figure S3.** HPLC chromatograms of *Salvia rosmarinus* samples subjected to drought and heat stresses, their combination, and chitosan application. Compounds are identified according to Table S2.
**Figure S4.** GC‐MS chromatograms of *Salvia rosmarinus* samples subjected to drought and heat stresses, their combination, and chitosan application. Major compounds are identified according to Table S3: 2 – α‐pinene (RT 6.67); 3 – camphene (RT 7.05); 13 – 1,8‐cineole (RT 9.71); 19 – linalool (RT 12.25); 21 – (+)‐camphor (RT 13.55); 25 – *endo*‐borneol (RT 14.49); 31 – l‐verbenone (RT 15.90); 35 – bornyl acetate (RT 18.44); 41–caryophyllene (RT 22.68).
**Table S1.** Summary of HPLC‐PAD criterion for quantification of phenolic compounds in *Salvia rosmarinus* extracts.
**Table S2.** Effect of drought and heat stresses, their combination, and the application of chitosan on the concentration of individual phenolic compounds (μg g_DW_^−1^ or mg g_DW_^−1^*; mean ± SE) of green extracts from *Salvia rosmarinus*, determined by HPLC‐PAD.
**Table S3.** Effect of drought and heat stresses, their combination, and the application of chitosan on the relative chemical composition (%) of essential oils from *Salvia rosmarinus* determined by GC‐MS.

## Data Availability

The data that support the findings of this study are available from the corresponding author upon reasonable request.
