# Supplementary material for: Chitosan‐induced modulation of secondary metabolism and stress tolerance in Salvia rosmarinus under combined drought and heat stress
Source: J Sci Food Agric. 2026 Apr 9;106(10):5989–6001. doi: 10.1002/jsfa.70652 (PMC13258217; doi:10.1002/jsfa.70652)
Supplement: Supplementary file 1 — Figure S1. Chlorophyll a and b of Salvia rosmarinus samples under drought and heat stresses, their combination, and the application of chitosan. The results were analyzed using one‐way analysis of variance (ANOVA) followed by Duncan's new multiple range test. Different letters mean significant differences (P < 0.05) among different treatments. CT, control; Ch, chitosan; D, drought; H, heat. Figure S2. Effect of chitosan on chlorophyll a/b ratio of Salvia rosmarinus samples under drought and heat stresses, and their combination. Percentages indicate relative change compared to baseline. CT, control; Ch, chitosan; D, drought; H, heat. Figure S3. HPLC chromatograms of Salvia rosmarinus samples subjected to drought and heat stresses, their combination, and chitosan application. Compounds are identified according to Table S2. Figure S4. GC‐MS chromatograms of Salvia rosmarinus samples subjected to drought and heat stresses, their combination, and chitosan application. Major compounds are identified according to Table S3: 2 – α‐pinene (RT 6.67); 3 – camphene (RT 7.05); 13 – 1,8‐cineole (RT 9.71); 19 – linalool (RT 12.25); 21 – (+)‐camphor (RT 13.55); 25 – endo‐borneol (RT 14.49); 31 – l‐verbenone (RT 15.90); 35 – bornyl acetate (RT 18.44); 41–caryophyllene (RT 22.68). Table S1. Summary of HPLC‐PAD criterion for quantification of phenolic compounds in Salvia rosmarinus extracts. Table S2. Effect of drought and heat stresses, their combination, and the application of chitosan on the concentration of individual phenolic compounds (μg gDW −1 or mg gDW −1*; mean ± SE) of green extracts from Salvia rosmarinus, determined by HPLC‐PAD. Table S3. Effect of drought and heat stresses, their combination, and the application of chitosan on the relative chemical composition (%) of essential oils from Salvia rosmarinus determined by GC‐MS. [file JSFA-106-5989-s001.docx]

**Chitosan-Induced Modulation of Secondary Metabolism and Stress Tolerance in *Salvia rosmarinus* under Combined Drought and Heat Stress**

Inês Mansinhos^a^, Sandra Gonçalves^a*^, Raquel Rodríguez-Solana^a,b^, Efrén Pérez-Santín^c^, María I. Fernández-Marín^b^, Emma Cantos-Villar^b^, Anabela Romano^a^

*^a^ MED – Mediterranean Institute for Agriculture, Environment and Development & CHANGE – Global Change and Sustainability Institute, Faculdade de Ciências e Tecnologia, Universidade do Algarve, Campus de Gambelas, 8005-139 Faro, Portugal*

*^b^ Department of Agroindustry and Food Quality, Andalusian Institute of Agricultural and Fisheries Research and Training (IFAPA), Rancho de La Merced Center, Carretera Cañada de La Loba (CA-3102) Km 3.1., SN, 11471 Jerez de la Frontera, Cádiz, Spain.*

*^c^ Escuela Superior de Ingeniería y Tecnología (ESIT), Universidad Internacional de La Rioja-UNIR, Avenida de la Paz, 137, 26006, Logroño, Spain*

** Correspondence: smgoncalves@ualg.pt*

**Supplementary information**

**Figure S1.** Chlorophyll a and b of *Salvia rosmarinus* samples under drought and heat stress, their combination, and the application of chitosan. The results were analyzed using one-way analysis of variance (ANOVA) followed by Duncan’s new multiple range test. Different letters mean significant differences (*p* < 0.05) among different treatments. CT, control; Ch, chitosan; D, drought; H, heat.


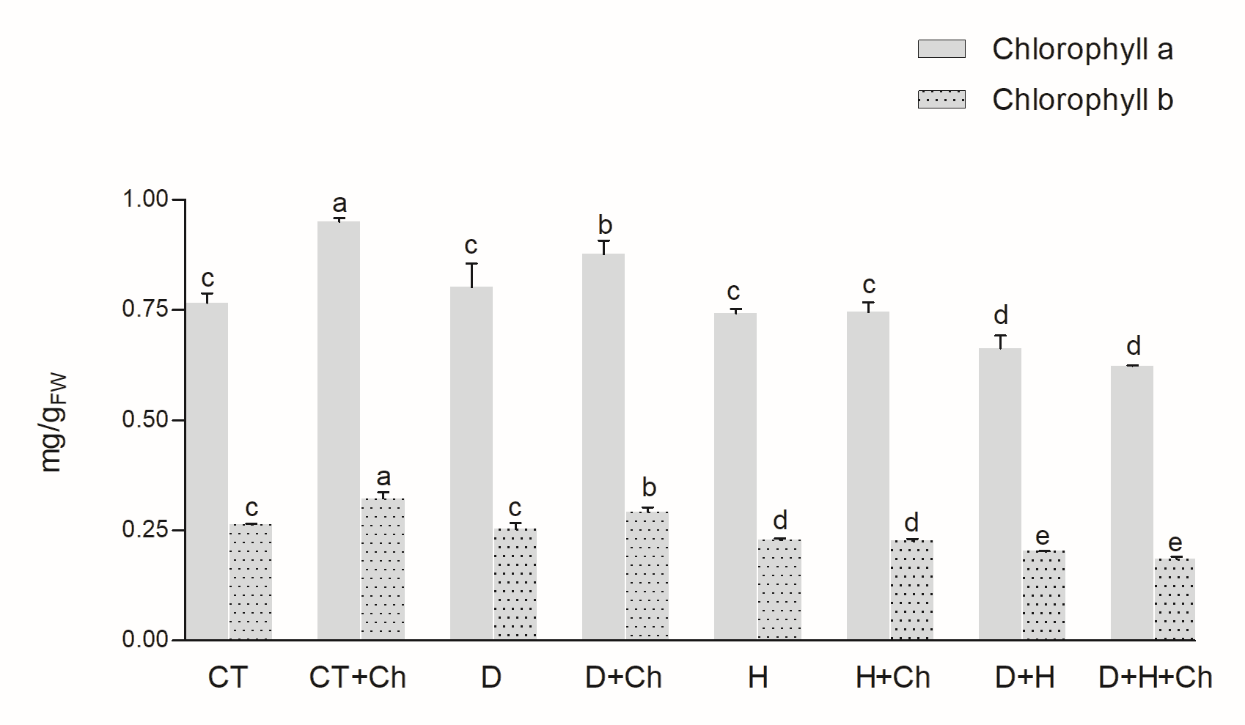


**Figure S2.** Effect of chitosan on chlorophyll a/b ratio of *Salvia rosmarinus* samples under drought and heat stress, their combination. Percentages indicate relative change compared to baseline. CT, control; Ch, chitosan; D, drought; H, heat.


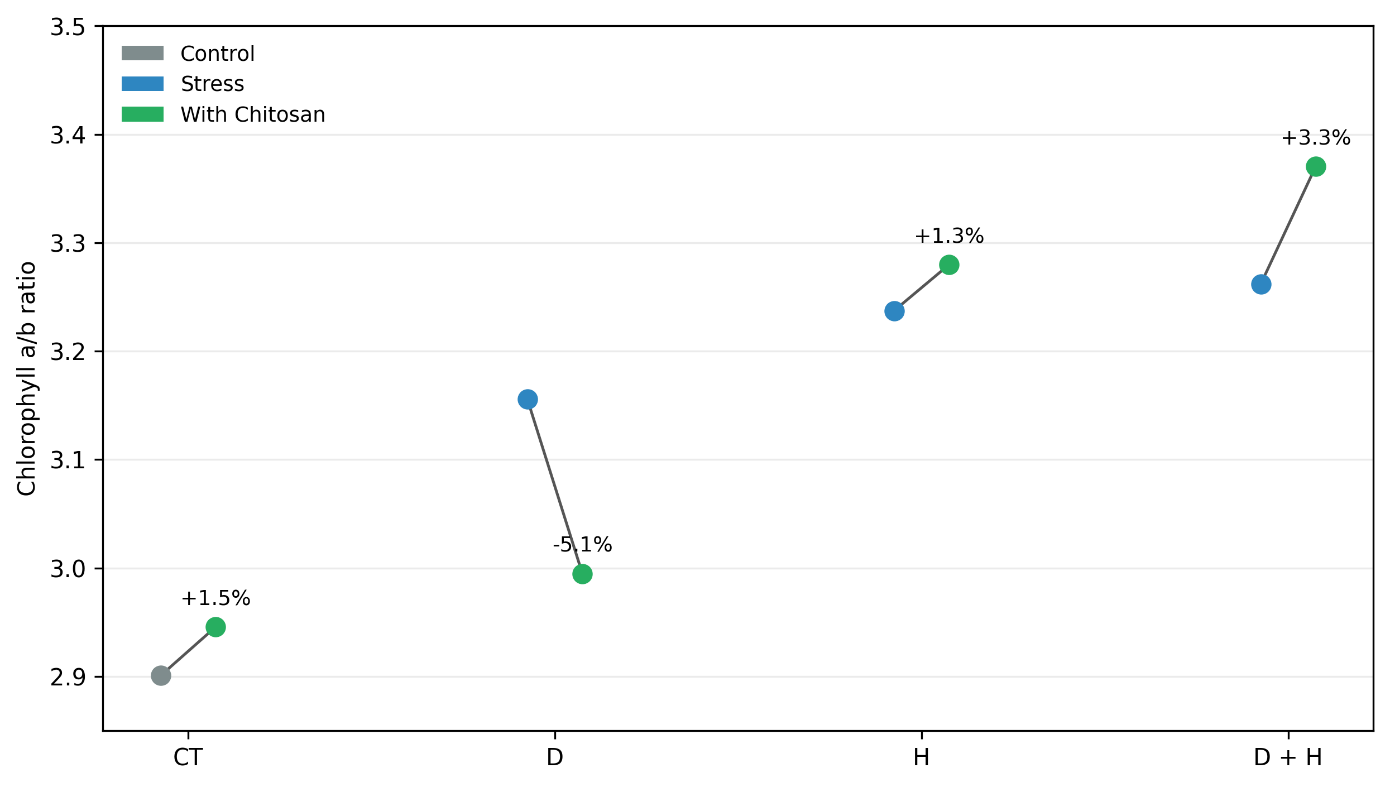


**Table S1.** Summary of HPLC-PAD criterion for quantification of phenolic compounds in *Salvia rosmarinus* extracts.

| Compound | RT (min) | Detection wavelength (nm) | Equation of calibration curve | R^2^ | LOD (mg/L) | LOQ (mg/L) | Range (mg/L) |
| --- | --- | --- | --- | --- | --- | --- | --- |
| Caffeic acid | 7.23 | 320 | y=114146x + 16940 | 0.9984 | 0.002 | 0.007 | 1–10 |
| Rosmarinic acid | 26.82 | 320 | y=57604x ‒ 115736 | 0.9993 | 0.004 | 0.013 | 10–50 |
| Hesperidin | 24.28 | 280 | y=39583x ‒ 393.2 | 0.9992 | 0.004 | 0.014 | 1–10 |
| Cinnamic acid | 32.1 | 280 | y = 172080x ‒ 16565 | 0.9989 | 0.002 | 0.005 | 1–10 |
| Carnosol | 61.63 | 280 | y= 6434.1 x + 1532.2 | 0.9992 | 0.06 | 0.18 | 1–10 |
| Carnosic acid | 63.23 | 280 | y = 4345.5x ‒ 1652.2 | 0.9994 | 0.22 | 0.74 | 1–10 |
| Luteolin-7-glucoside | 20.66 | 360 | y = 53137x ‒ 4650.6 | 0.9996 | 0.004 | 0.013 | 2.5–40 |
| Apigenin-7-glucoside | 27.2 | 360 | y = 44665x ‒ 3979.8 | 0.9994 | 0.02 | 0.05 | 1–10 |

LOD – limit of detection; LOQ – limit of quantification.

| Table S2. Effect of drought and heat stress, their combination, and the application of chitosan on the concentration of individual phenolic compounds (µg/g_DW_ mg/g_DW_*; mean ± SE) of green extracts from *Salvia rosmarinus*, determined by HPLC-PAD. | | | | | | | | | | | |
| --- | --- | --- | --- | --- | --- | --- | --- | --- | --- | --- | --- |
| Compounds | **Class** | **RT** | **Treatments** | | | | | | | | |
|  |  |  | **CT** | **CT + Ch** | **D** | **D + Ch** | **H** | | **H + Ch** | **D + H** | **D + H + Ch** |
| Caffeic acid | Phenolic acid | 7.23 | 67.14 ± 1.14 c | 65.51 ± 0.74 c | 114.86 ± 0.59 b | 128.59 ± 1.07 a | 64.86 ± 0.68 c | | 45.27 ± 1.09 e | 61.43 ± 1.37 d | 60.62 ± 0.59 d |
| Rosmarinic acid | Phenolic acid | 26.82 | 4902.88 ± 11.24 g | 3410.76 ± 8.97 h | 9216.90 ± 31.32 d | 6436.42 ± 6.96 f | 8673.62 ± 6.05 e | | 13430.41 ± 8.90 c | 13856.44 ± 21.12 b | 16557.78 ± 18.64 a |
| Hesperidin | Flavonoid | 24.28 | 81.95 ± 0.85 d | 102.65 ± 3.38 c | 117.59 ± 3.58 b | 96.57 ± 5.54 c | 71.90 ± 1.90 e | | 95.59 ± 1.47 c | 103.78 ± 1.42 c | 152.58 ± 3.58 a |
| Unknown 1 | Possible cinnamic acid derivative | 37.94 | 241.53 ± 2.06 d | 143.20 ± 0.48 g | 212.51 ± 1.05 f | 132.41 ± 1.94 h | 246.96 ± 1.02 c | | 234.07 ± 2.02 e | 327.87 ± 1.69 b | 350.78 ± 1.33 a |
| Unknown 2 | Possible cinnamic acid derivative | 41.98 | 368.01 ± 7.99 a | 223.86 ± 3.13 e | 304.58 ± 3.77 b | 175.46 ± 2.96 f | 175.48 ± 1.75 f | | 151.69 ± 1.05 g | 246.35 ± 1.65 d | 290.96 ± 1.26 c |
| Carnosol | Phenolic diterpene | 61.63 | 578.72 ± 6.20 e | 1351.83 ± 13.28 b | 882.13 ± 5.12 c | 418.08 ± 15.54 f | 656.02 ± 9.05 d | | 648.79 ± 7.95 d | 656.94 ± 10.86 d | 1613.51 ± 4.49 a |
| Carnosic acid | Phenolic diterpene | 63.23 | 850.16 ± 25.00 b | 1965.04 ± 27.46 a | 572.86 ± 12.47 c | 468.84 ± 7.35 d | 195.43 ± 6.61 fg | | 157.47 ± 0.61 g | 355.67 ± 5.36 e | 234.72 ± 8.25 f |
| Unknown 3 | Possible flavonoid | 16.08 | 274.23 ± 4.91 e | 178.41 ± 4.12 f | 429.51 ± 4.82 b | 511.19 ± 10.12 a | 355.34 ± 10.80 c | | 438.02 ± 6.49 b | 327.21 ± 5.95 d | 337.39 ± 12.80 cd |
| Luteolin-7-glucuronide | Flavonoid | 21.06 | 324.20 ± 3.28 e | 228.45 ± 5.00 g | 559.06 ± 8.60 b | 616.40 ± 6.12 a | 342.37 ± 2.54 d | | 471.83 ± 4.22 c | 345.11 ± 3.79 d | 290.13 ± 1.16 f |
| Unknown 4 | Possible apigenin-hexoside derivative | 21.6 | 131.10 ± 1.83 cd | 111.41 ± 8.90 d | 205.99 ± 15.31 a | 212.78 ± 14.47 a | 145.16 ± 2.66 bc | | 166.41 ± 9.71 b | 142.26 ± 7.87 bc | 137.01 ±1.65 bcd |
| Unknown 5 | Possible luteolin-hexoside derivative | 24.71 | 1631.74 ± 0.60 f | 1467.45 ± 5.29 g | 2171.48 ± 7.52 b | 2189.99 ± 4.03 a | 1803.35 ± 4.18 e | | 2033.53 ± 6.66 c | 1859.71 ± 3.51 d | 2169.67 ± 5.10 b |
| Unknown 6 | Possible apigenin-hexoside derivative | 31.13 | 323.63 ± 3.98 f | 312.87 ± 6.21 f | 481.85 ± 10.51 a | 464.84 ± 7.01 b | 344.86 ± 2.63 e | | 410.06 ± 2.21 d | 400.49 ± 2.38 d | 445.88 ± 3.62 c |
| Luteolin-3´-glucuronide | Flavonoid | 36.44 | 890.24 ± 6.68 ef | 800.68 ± 5.79 g | 1321.99 ± 10.40 b | 1580.57 ± 4.94 a | 877.10 ± 4.00 f | | 1084.98 ± 6.99 c | 901.62 ± 8.33 e | 927.85 ± 11.68 d |
| Unknown 7 | Possible apigenin-hexoside derivative | 40.06 | 343.60 ± 1.74 b | 372.08 ± 7.13 a | 299.41 ± 4.16 d | 237.54 ± 6.57 f | 272.74 ± 2.21 e | | 248.65 ± 2.58 f | 326.44 ± 5.64 c | 341.37 ± 7.16 bc |
| Unknown 8 | Possible luteolin-hexoside derivative | 44.12 | 800.32 ± 14.56 c | 621.48 ± 8.15 e | 1147.35 ±13.07 b | 1548.47 ± 27.89 a | | 564.41 ± 3.86 f | 710.14 ± 12.29 d | 558.85 ± 5.08 f | 658.81 ± 8.50 e |
| Unknown 9 | Possible apigenin-hexoside derivative | 46.3 | 522.51 ± 2.30 e | 448.74 ± 11.63 f | 590.87 ± 4.40 d | 785.32 ± 11.32 a | 630.03 ± 6.49 c | | 750.98 ± 12.49 b | 534.10 ± 3.55 e | 539.24 ± 14.15 e |
| Unknown 10 | Possible apigenin-hexoside derivative | 49.3 | 397.44 ± 7.94 b | 407.71 ± 8.84 b | 404.74 ± 6.28 b | 332.57 ± 7.06 d | 347.47 ± 11.24 cd | | 368.27 ± 11.28 c | 425.58 ± 11.16 b | 453.30 ± 8.62 a |
| Unknown 11 | Possible apigenin-hexoside derivative | 49.9 | 620.23 ± 5.28 d | 497.66 ± 1.65 f | 1252.60 ± 5.87 b | 1316.90 ± 1.22 a | 499.66 ± 3.89 f | | 612.18 ± 3.37 d | 565.51 ± 0.62 e | 680.09 ± 11.99 c |
| Total phenolic content* |  |  | **13.35 ± 0.04 f** | **12.71 ± 0.07 g** | **20.29 ± 0.11 c** | **17.65 ± 0.04 d** | **16.27 ± 0.02 e** | | **22.06 ± 0.04 b** | **22.00 ± 0.04b** | **26.24 ± 0.01 a** |

CT, control; Ch, chitosan; D, drought; H, heat. The results were analyzed using one-way analysis of variance (ANOVA) followed by Duncan’s new multiple range test. Different letters mean significant differences (*p* < 0.05) among different treatments.

|  | Table S3. Effect of drought and heat stress, their combination, and the application of chitosan on the relative chemical composition (%) of essential oils from *Salvia rosmarinus* determined by GC-MS. | | | | | | | | | | | |
| --- | --- | --- | --- | --- | --- | --- | --- | --- | --- | --- | --- | --- |
|  | **Compounds** | **Class** | **RI^*^** | **RI literature** | **Composition (%)** | | | | | | | |
|  |  |  |  |  | **CT** | **CT + Ch** | **D** | **D + Ch** | **H** | **H + Ch** | **D + H** | **D + H + Ch** |
| 1 | Cyclofenchene | MH | 917 | 927 | 0.03 ± 0.00 f | 0.16 ± 0.00 c | 0.06 ± 0.00 e | 0.17 ± 0.00 c | 0.06 ± 0.01 e | 0.25 ± 0.00 a | 0.13 ± 0.00 d | 0.21 ± 0.01 b |
| 2 | **α-Pinene** | **MH** | **929** | **929** | **4.29 ± 0.23 f** | **13.17 ± 0.20 b** | **7.35 ± 0.18 d** | **14.12 ± 0.07 b** | **5.80 ± 0.31 e** | **16.93 ± 0.09 a** | **10.35 ± 0.19 c** | **17.40 ± 0.86 a** |
| 3 | **Camphene** | **MH** | **942** | **942** | **1.03 ± 0.08 f** | **2.88 ± 0.04 c** | **1.59 ± 0.05 e** | **3.08 ± 0.01 c** | **1.54 ± 0.06 e** | **4.07 ± 0.01 a** | **2.64 ± 0.05 d** | **3.41 ± 0.15 b** |
| 4 | α-Sabinene | MH | 948 | 947 | 0.33 ± 0.02 g | 1.37 ± 0.02 b | 0.53 ± 0.02 f | 1.31 ± 0.01 bc | 0.65 ± 0.04 e | 1.22 ± 0.00 c | 1.10 ± 0.02 d | 1.88 ± 0.09 a |
| 5 | 3,7,7-Trimethyl-1,3,5-cycloheptatriene | MH | 965 | 970 | 0.06 ± 0.00 e | 0.15 ± 0.00 b | 0.09 ± 0.00 d | 0.16 ± 0.00 b | 0.10 ± 0.00 d | 0.13 ± 0.00 c | 0.13 ± 0.00 c | 0.18 ± 0.01 a |
| 6 | β-Sabinene | MH | 970 | 970 | 0.37 ± 0.02 e | 0.58 ± 0.01 c | 0.61 ± 0.02 c | 0.71 ± 0.01 b | 0.50 ± 0.02 d | 1.36 ± 0.01 a | 0.70 ± 0.01 b | 0.63 ± 0.03 c |
| 7 | 1-Octen-3-ol | OM | 978 | 978 | 0.05 ± 0.01 e | 0.13 ± 0.01 b | 0.07 ± 0.01 d | 0.13 ± 0.00 b | 0.08 ± 0.01 cd | 0.09 ± 0.00 c | 0.06 ± 0.00 d | 0.23 ± 0.01 a |
| 8 | β-Pinene | MH | 989 | 989 | 0.49 ± 0.02 e | 0.86 ± 0.01 c | 0.68 ± 0.01 d | 0.94 ± 0.00 bc | 0.65 ± 0.03 d | 1.00 ± 0.00 b | 0.74 ± 0.01 d | 1.26 ± 0.10 a |
| 9 | α-Phellandrene | MH | 1001 | 1001 | 0.21 ± 0.01 d | 0.36 ± 0.00 b | 0.30 ± 0.01 c | 0.40 ± 0.00 b | 0.30 ± 0.01 c | 0.39 ± 0.00 b | 0.35 ± 0.00 b | 0.59 ± 0.04 a |
| 10 | 3-Carene | MH | 1006 | 1006 | 1.14 ± 0.05 f | 1.94 ± 0.02 cd | 1.61 ± 0.03 e | 2.11 ± 0.00 ab | 1.58 ± 0.06 e | 1.81 ± 0.00 d | 1.99 ± 0.02 bc | 2.18 ± 0.10 a |
| 11 | (+)-4-Carene | MH | 1013 | 1014 | 0.14 ± 0.01 f | 0.30 ± 0.00 c | 0.21 ± 0.00 e | 0.36 ± 0.00 b | 0.19 ± 0.01 e | 0.31 ± 0.00 c | 0.25 ± 0.00 d | 0.49 ± 0.03 a |
| 12 | o-Cymene | MH | 1020 | 1020 | 0.69 ± 0.02 d | 1.40 ± 0.01 a | 0.96 ± 0.02 c | 1.40 ± 0.00 a | 0.94 ± 0.04 c | 1.09 ± 0.01 b | 1.14 ± 0.01 b | 1.51 ± 0.09 a |
| 13 | **1,8-Cineole** | **OM** | **1026** | **1026** | **5.98 ± 0.19 e** | **10.91 ± 0.09 bc** | **10.11 ± 0.44 c** | **11.63 ± 0.02 b** | **8.70 ± 0.49 d** | **11.97 ± 0.04 ab** | **9.85 ± 0.08 c** | **12.83 ± 0.76 a** |
| 14 | *trans*-β-Ocimene | MH | 1038 | 1038 | 0.05 ± 0.00 c | 0.02 ± 0.00 e | 0.07 ± 0.00 b | 0.03 ± 0.00 de | 0.03 ± 0.00 de | 0.04 ± 0.00 cd | 0.04 ± 0.00 de | 0.24 ± 0.01 a |
| 15 | γ-Terpinene | MH | 1055 | 1055 | 0.49 ± 0.01 bc | 0.45 ± 0.00 c | 0.53 ± 0.02 b | 0.54 ± 0.00 b | 0.53 ± 0.01 b | 0.62 ± 0.00 a | 0.47 ± 0.00 c | 0.60 ± 0.03 a |
| 16 | *cis*-Sabinene hydrate | OM | 1063 | 1063 | 0.14 ± 0.01 a | 0.02 ± 0.00 f | 0.14 ± 0.00 a | 0.04 ± 0.00 e | 0.09 ± 0.00 c | 0.11 ± 0.00 b | 0.09 ± 0.00 d | 0.02 ± 0.00 f |
| 17 | Terpinolene | MH | 1084 | 1084 | 1.08 ± 0.03 cd | 1.06 ± 0.00 d | 1.21 ± 0.03 b | 1.15 ± 0.02 bcd | 1.14 ± 0.02 bcd | 1.20 ± 0.00 bc | 1.13 ± 0.02 bcd | 1.39 ± 0.08 a |
| 18 | 1-Terpinenol | OM | 1094 | 1096 | 0.19 ± 0.01 bc | 0.40 ± 0.16 ab | 0.18 ± 0.00 bc | 0.37 ± 0.16 abc | 0.14 ± 0.00 bc | 0.36 ± 0.02 abc | 0.12 ± 0.00 c | 0.52± 0.03 a |
| 19 | **Linalool** | **OM** | **1101** | **1101** | **5.38 ± 0.05 a** | **3.93 ± 0.18 c** | **4.78 ± 0.16 b** | **3.80 ± 0.17 cd** | **4.81 ± 0.07 b** | **3.38 ± 0.03 d** | **4.49 ± 0.04 b** | **3.72 ± 0.22 cd** |
| 20 | Chrysanthenone | OM | 1119 | 1119 | 1.29 ± 0.02 b | 1.25 ± 0.00 bc | 1.29 ± 0.04 b | 1.04 ± 0.00 d | 1.26 ± 0.02 bc | 1.17 ± 0.01 c | 1.32 ± 0.01 b | 1.49 ± 0.09 a |
| 21 | **(+)-Camphor** | **OM** | **1139** | **1141** | **10.31 ± 0.14 d** | **13.41 ± 0.07 b** | **11.81 ± 0.13 c** | **11.79 ± 0.25 c** | **10.67 ± 0.12 d** | **12.04 ± 0.05 c** | **10.52 ± 0.05 d** | **14.59 ± 0.92 a** |
| 22 | cis-Verbenol | OM | 1142 | 1142 | 1.41 ± 0.13 a | n.d | 1.10 ± 0.06 bc | n.d. | 1.34 ± 0.11 ab | n.d. | 0.99 ± 0.02 c | n.d. |
| 23 | Isocamphopinone | OM | 1154 | 1154 | 0.42 ± 0.02 bc | 0.44 ± 0.01 ab | 0.48 ± 0.03 a | 0.43 ± 0.01 bc | 0.43 ± 0.00 bc | 0.24 ± 0.01 d | 0.46 ± 0.01 ab | 0.38 ± 0.03 c |
| 24 | Pinocarvone | OM | 1156 | 1156 | 0.24 ± 0.01 ab | 0.24 ± 0.01 ab | 0.27 ± 0.03 a | 0.22 ± 0.01 ab | 0.22 ± 0.01 ab | 0.21 ± 0.01 b | 0.21 ± 0.01 b | 0.24 ± 0.02 ab |
| 25 | **endo-Borneol** | **OM** | **1166** | **1166** | **17.47 ± 0.18 a** | **12.40 ± 0.11 e** | **14.40 ± 0.38 c** | **13.42 ± 0.03 d** | **16.26 ± 0.22 b** | **11.91 ± 0.03 e** | **14.78 ± 0.10 c** | **10.89 ± 0.64 f** |
| 26 | *trans*-3-Pinanone | OM | 1169 | 1169 | 2.11 ± 0.03 ab | 1.86 ± 0.03 c | 2.13 ± 0.10 ab | 1.83 ± 0.06 c | 1.78 ± 0.03 c | 1.90 ± 0.09 bc | 2.26 ± 0.06 a | 1.53 ± 0.15 d |
| 27 | Terpinen-4-ol | OM | 1174 | 1174 | 0.94 ± 0.05 ab | 0.88 ± 0.01 ab | 0.85 ± 0.05 ab | 0.87 ± 0.04 ab | 0.82 ± 0.01 ab | 0.89 ± 0.05 ab | 0.79 ± 0.03 b | 0.99 ± 0.08 a |
| 28 | 3,9-Epoxy-p-mentha-1,8(10)-diene | OM | 1187 | 1199 | n.d. | 0.25 ± 0.01 b | 0.27 ± 0.03 b | 0.24 ± 0.00 bc | 0.31 ± 0.01 b | 0.17 ± 0.02 c | 1.62 ± 0.03 a | n.d. |
| 29 | α-Terpineol | OM | 1190 | 1190 | 2.61 ± 0.05 a | 1.73 ± 0.02 cd | 2.24 ± 0.10 b | 1.63 ± 0.01 d | 1.85 ± 0.02 c | 1.60 ± 0.07 d | n d. | 0.27 ± 0.02 e |
| 30 | (-)-Myrtenol | OM | 1198 | 1212.8 | n.d. | 0.09 ± 0.02 b | n.d. | 0.11 ± 0.03 ab | n.d. | 0.18 ± 0.01 a | n.d. | n.d. |
| 31 | **l-Verbenone** | **OM** | **1206** | **1204** | **12.40 ± 0.11 bc** | **14.41 ± 0.05 a** | **11.61 ± 0.35 de** | **11.40 ± 0.11 e** | **12.02 ± 0.04 cd** | **9.57 ± 0.03 f** | **9.91 ± 0.12 f** | **12.59 ± 0.43 b** |
| 32 | *trans*-Shisool | OM | 1234 | 1225 | 0.83 ± 0.03 a | 0.62 ± 0.05 ab | 0.62 ± 0.07 ab | 0.52 ± 0.12 b | 0.74 ± 0.11 ab | 0.64 ± 0.05 ab | 0.49 ± 0.13 b | 0.65 ± 0.06 ab |
| 33 | Myrtanol | OM | 1239 | 1254 | 1.34 ± 0.23 a | 1.30 ± 0.04 a | 1.22 ± 0.07 a | 1.00 ± 0.06 a | 1.30 ± 0.20 a | 1.25 ± 0.05 a | 1.02 ± 0.23 a | 1.28 ± 0.06 a |
| 34 | *trans*-p-Menth-2-en-7-ol | OM | 1246 | 1248 | 0.10 ± 0.03 ab | 0.13 ± 0.02 ab | 0.25 ± 0.11 a | 0.02 ± 0.00 b | 0.04 ± 0.01 b | 0.05 ± 0.01 b | 0.11 ± 0.09 ab | 0.06 ± 0.02 b |
| 35 | **Bornyl acetate** | **OM** | **1283** | **1283** | **5.84 ± 0.25 a** | **1.44 ± 0.02 e** | **4.44 ± 0.38 b** | **1.98 ± 0.01 d** | **4.82 ± 0.04 b** | **3.46 ± 0.03 c** | **3.77 ± 0.06 c** | **0.89 ± 0.04 f** |
| 36 | Eugenol | Others | 1354 | 1354 | 0.26 ± 0.01 b | 0.12 ± 0.00 ef | 0.23 ± 0.01 c | 0.10 ± 0.00 f | 0.30 ± 0.01 a | 0.09 ± 0.00 g | 0.15 ± 0.00 d | 0.13 ± 0.01 de |
| 37 | *cis*-Myrtanyl acetate | OM | 1359 | 1365 | 0.28 ± 0.01 a | 0.10 ± 0.00 d | 0.24 ± 0.01 b | 0.12 ± 0.00 d | 0.24 ± 0.00 b | 0.18 ± 0.00 c | 0.18 ± 0.00 c | 0.08 ± 0.00 e |
| 38 | δ-EIemene | SH | 1365 | 1365 | 0.71 ± 0.01 a | 0.18 ± 0.00 e | 0.58 ± 0.03 b | 0.21 ± 0.00 e | 0.59 ± 0.01 b | 0.49 ± 0.00 c | 0.34 ± 0.00 d | 0.13 ± 0.01 f |
| 39 | Copaene | SH | 1371 | 1371 | 0.77 ± 0.02 a | 0.39 ± 0.01 e | 0.62 ± 0.03 c | 0.46 ± 0.00 d | 0.70 ± 0.01 b | 0.31 ± 0.00 f | 0.68 ± 0.01 b | 0.37 ± 0.02 e |
| 40 | Methyleugenol | Others | 1402 | 1402 | 0.62 ± 0.02 a | 0.22 ± 0.00 d | 0.43 ± 0.04 b | 0.30 ± 0.00 c | 0.44 ± 0.01 b | 0.31 ± 0.00 c | 0.31 ± 0.00 c | 0.17 ± 0.01 d |
| 41 | **Caryophyllene** | **SH** | **1415** | **1415** | **7.24 ± 0.29 a** | **3.85 ± 0.06 cd** | **5.92 ± 0.46 b** | **4.27 ± 0.01 c** | **6.57 ± 0.06 b** | **3.26 ± 0.02 d** | **6.48 ± 0.08 b** | **4.22 ± 0.24 c** |
| 42 | β-Cubebene | SH | 1423 | 1423 | 0.13 ± 0.01 a | 0.05 ± 0.00 d | 0.12 ± 0.01 bc | 0.06 ± 0.00 d | 0.13 ± 0.00 ab | 0.05 ± 0.00 d | 0.11 ± 0.00 c | 0.05 ± 0.00 d |
| 43 | Alloaromadendrene | SH | 1433 | 1432 | 0.17 ± 0.01 a | 0.08 ± 0.00 e | 0.12 ± 0.01 c | 0.11 ± 0.00 d | 0.16 ± 0.00 ab | 0.07 ± 0.00 e | 0.15 ± 0.00 b | 0.08 ± 0.00 e |
| 44 | Humulene | SH | 1448 | 1448 | 1.27 ± 0.05 a | 0.66 ± 0.01 de | 1.02 ± 0.07 c | 0.74 ± 0.02 d | 1.14 ± 0.01 b | 0.57 ± 0.01 e | 1.08 ± 0.01 bc | 0.71 ± 0.04 d |
| 45 | *trans*-Geranylacetone | Others | 1450 | 1450 | 0.22 ± 0.01 a | 0.18 ± 0.00 bc | 0.21 ± 0.02 a | 0.15 ± 0.02 cd | 0.17 ± 0.00 bcd | 0.19 ± 0.00 ab | 0.14 ± 0.00 d | 0.20 ± 0.01 ab |
| 46 | γ-Muurolene | SH | 1472 | 1472 | 0.80 ± 0.03 a | 0.35 ± 0.01 e | 0.59 ± 0.07 c | 0.45 ± 0.00 d | 0.72 ± 0.01 ab | 0.29 ± 0.00 e | 0.67 ± 0.00 bc | 0.33 ± 0.02 e |
| 47 | δ-Guaiene | SH | 1480 | 1482 | 0.18 ± 0.01 a | 0.08 ± 0.01 d | 0.15 ± 0.02 b | 0.11 ± 0.00 c | 0.16 ± 0.00 ab | 0.05 ± 0.01 d | 0.15 ± 0.00 b | 0.08 ± 0.00 d |
| 48 | Valencene | SH | 1489 | 1489 | 0.38 ± 0.02 a | 0.15 ± 0.01 e | 0.25 ± 0.02 c | 0.19 ± 0.00 d | 0.35 ± 0.00 a | 0.12 ± 0.01 e | 0.31 ± 0.00 b | 0.15 ± 0.01 e |
| 49 | α-Muurolene | SH | 1495 | 1495 | 0.42 ± 0.02 a | 0.18 ± 0.01 d | 0.25 ± 0.04 c | 0.24 ± 0.00 c | 0.37 ± 0.00 ab | 0.13 ± 0.01 d | 0.34 ± 0.00 b | 0.18 ± 0.01 d |
| 50 | β-Bisabolene | SH | 1506 | 1506 | 0.31 ± 0.02 a | 0.20 ± 0.07 bc | 0.22 ± 0.02 b | 0.17 ± 0.00 bcd | 0.25 ± 0.00 ab | 0.10 ± 0.00 d | 0.24 ± 0.01 ab | 0.12 ± 0.01 cd |
| 51 | γ-Cadinene | SH | 1509 | 1509 | 0.77 ± 0.04 a | 0.34 ± 0.01 e | 0.52 ± 0.04 c | 0.43 ± 0.00 d | 0.70 ± 0.01 b | 0.27 ± 0.00 e | 0.65 ± 0.02 b | 0.32 ± 0.02 e |
| 52 | δ-Cadinene | SH | 1519 | 1519 | 1.92 ± 0.10 a | 0.84 ± 0.02 e | 1.27 ± 0.06 c | 1.07 ± 0.00 d | 1.66 ± 0.00 b | 0.68 ± 0.00 e | 1.58 ± 0.08 b | 0.82 ± 0.04 e |
| 53 | Caryophyllene oxide | OS | 1575 | 1575 | 1.66 ± 0.14 a | 0.75 ± 0.02 c | 1.31 ± 0.09 b | 0.93 ± 0.00 c | 1.38 ± 0.02 b | 0.75 ± 0.00 c | 1.22 ± 0.02 b | 0.46 ± 0.02 d |
| 54 | Humulene II epoxide | OS | 1601 | 1601 | 0.28 ± 0.02 a | 0.12 ± 0.00 d | 0.23 ± 0.03 b | 0.17 ± 0.00 c | 0.22 ± 0.01 b | 0.11 ± 0.00 de | 0.19 ± 0.00 bc | 0.08 ± 0.00 e |
| 55 | α-Muurolol | OS | 1619 | 1619 | 0.18 ± 0.02 a | 0.07 ± 0.00 cd | 0.12 ± 0.01 b | 0.09 ± 0.00 c | 0.14 ± 0.00 b | 0.06 ± 0.00 d | 0.09 ± 0.00 c | 0.05 ± 0.00 d |
| 56 | τ-Cadinol | OS | 1631 | 1631 | 0.54 ± 0.01 a | 0.21 ± 0.01 de | 0.42 ± 0.05 b | 0.26 ± 0.03 cd | 0.54 ± 0.02 a | 0.15 ± 0.00 e | 0.31 ± 0.01 c | 0.15 ± 0.00 e |
| 57 | Cubenol | OS | 1638 | 1638 | 0.27 ± 0.05 a | 0.09 ± 0.01 bc | 0.25 ± 0.03 a | 0.04 ± 0.02 c | 0.24 ± 0.01 a | 0.02 ± 0.00 c | 0.12 ± 0.01 b | 0.06 ± 0.00 bc |
| 58 | α-Cadinol | OS | 1644 | 1644 | 0.24 ± 0.01 a | 0.06 ± 0.01 de | 0.18 ± 0.02 b | 0.08 ± 0.01 cd | 0.23 ± 0.01 a | 0.04 ± 0.00 e | 0.10 ± 0.01 c | 0.03 ± 0.00 e |
| 59 | α-Bisabolol | OS | 1676 | 1676 | 0.54 ± 0.01 a | 0.16 ± 0.01 e | 0.41 ± 0.02 c | 0.25 ± 0.00 d | 0.44 ± 0.00 b | 0.11 ± 0.00 f | 0.26 ± 0.01 d | 0.11 ± 0.01 f |
| 60 | Farnesyl acetone | Others | 1913 | 1913 | 0.24 ± 0.02 a | 0.05 ± 0.00 c | 0.24 ± 0.02 a | 0.08 ± 0.00 c | 0.18 ± 0.01 b | 0.05 ± 0.00 c | 0.06 ± 0.00 c | 0.06 ± 0.00 c |
| 61 | Phytol | Others | 2142 | 2142 | 0.13 ± 0.03 b | n.d. | 0.14 ± 0.01 b | n.d. | 0.31 ± 0.03 a | n.d. | 0.01 ± 0.01 | n.d. |
|  | **Grouped compounds** | | | |  |  |  |  |  |  |  |  |
|  | Monoterpene hydrocarbons (MH) | | | | 10.42 ± 0.41 g | 24.72 ± 0.31 c | 15.79 ± 0.25 e | 26.49 ± 0.09 b | 14.01 ± 0.55 f | 30.41 ± 0.11 a | 21.17 ± 0.30 d | 31.98 ± 1.44 a |
|  | Oxygenated monoterpenes (OM) | | | | 69.32 ± 0.98 a | 65.93 ± 0.12 b | 68.41 ± 0.89 ab | 62.82 ± 0.23 c | 67.90 ± 0.42 ab | 61.30 ± 0.05 cd | 63.07 ± 0.52 c | 59.03 ± 1.71 d |
|  | Sesquiterpene hydrocarbons (SH) | | | | 15.07 ± 0.55 a | 7.33 ± 0.15 de | 11.63 ± 0.81 c | 8.50 ± 0.04 d | 13.50 ± 0.10 b | 6.41 ± 0.05 e | 12.78 ± 0.20 bc | 7.56 ± 0.45 de |
|  | Oxygenated sesquiterpenes (OS) | | | | 3.71 ± 0.16 a | 1.46 ± 0.06 e | 2.91 ± 0.25 b | 1.80 ± 0.06 d | 3.20 ± 0.05 b | 1.24 ± 0.01 ef | 2.30 ± 0.06 c | 0.94 ± 0.03 f |
|  | Others | | | | 1.48 ± 0.07 a | 0.56 ± 0.01 c | 1.25 ± 0.09 b | 0.63 ± 0.02 c | 1.39 ± 0.02 a | 0.64 ± 0.01 c | 0.67 ± 0.01 c | 0.56 ± 0.03 c |

n.d., not defined; CT, control; Ch, chitosan; D, drought; H, heat. ^*^RI. Retention Index relative to C8-C40 n-alkanes on the HP-5MS capillary column. The results were analyzed using one-way analysis of variance (ANOVA) followed by Duncan’s new multiple range test. Different letters mean significant differences (*p* < 0.05) among different treatments.

**Figure S3.** HPLC chromatograms of *Salvia rosmarinus* samples subjected to drought stress, heat stress, their combination, and chitosan application. Compounds are identified according to Table S2.

| **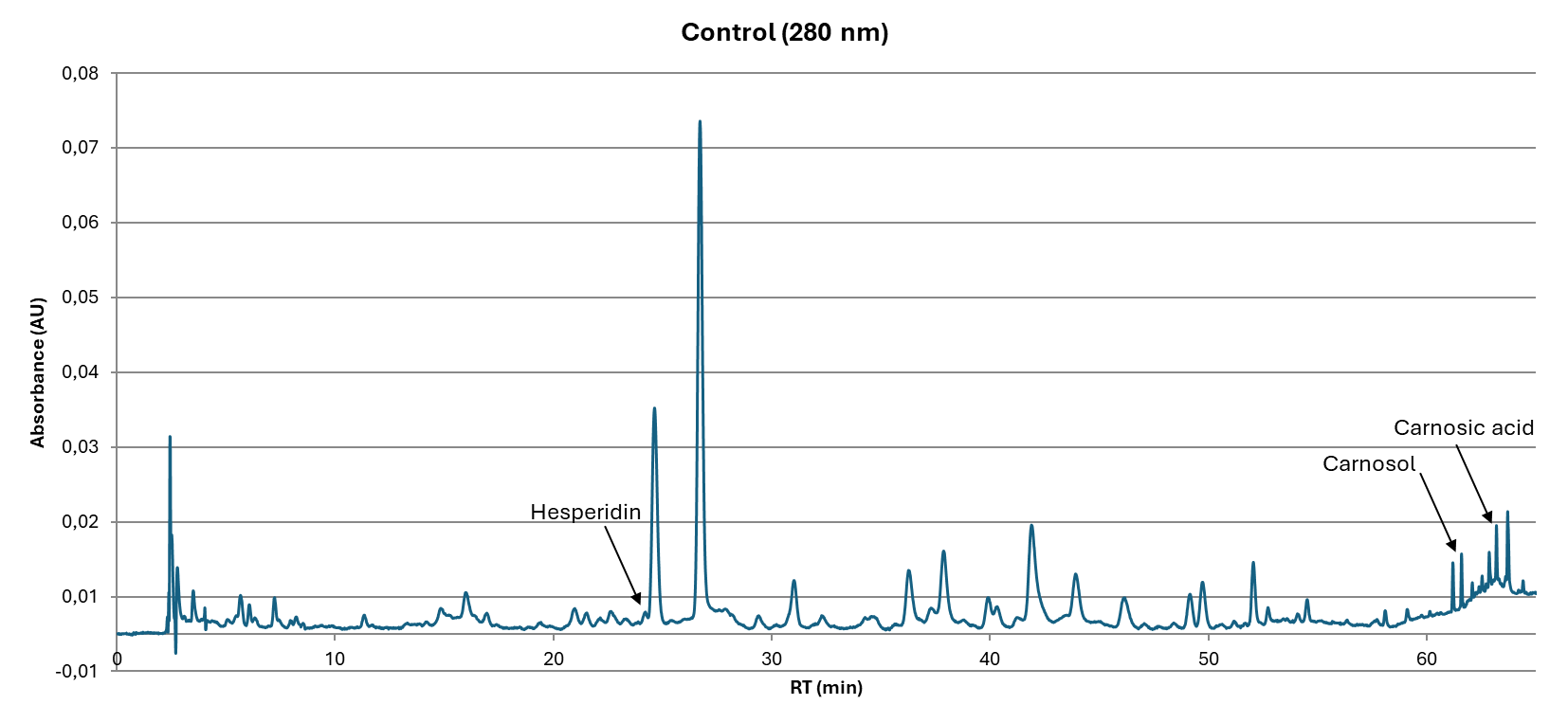** | **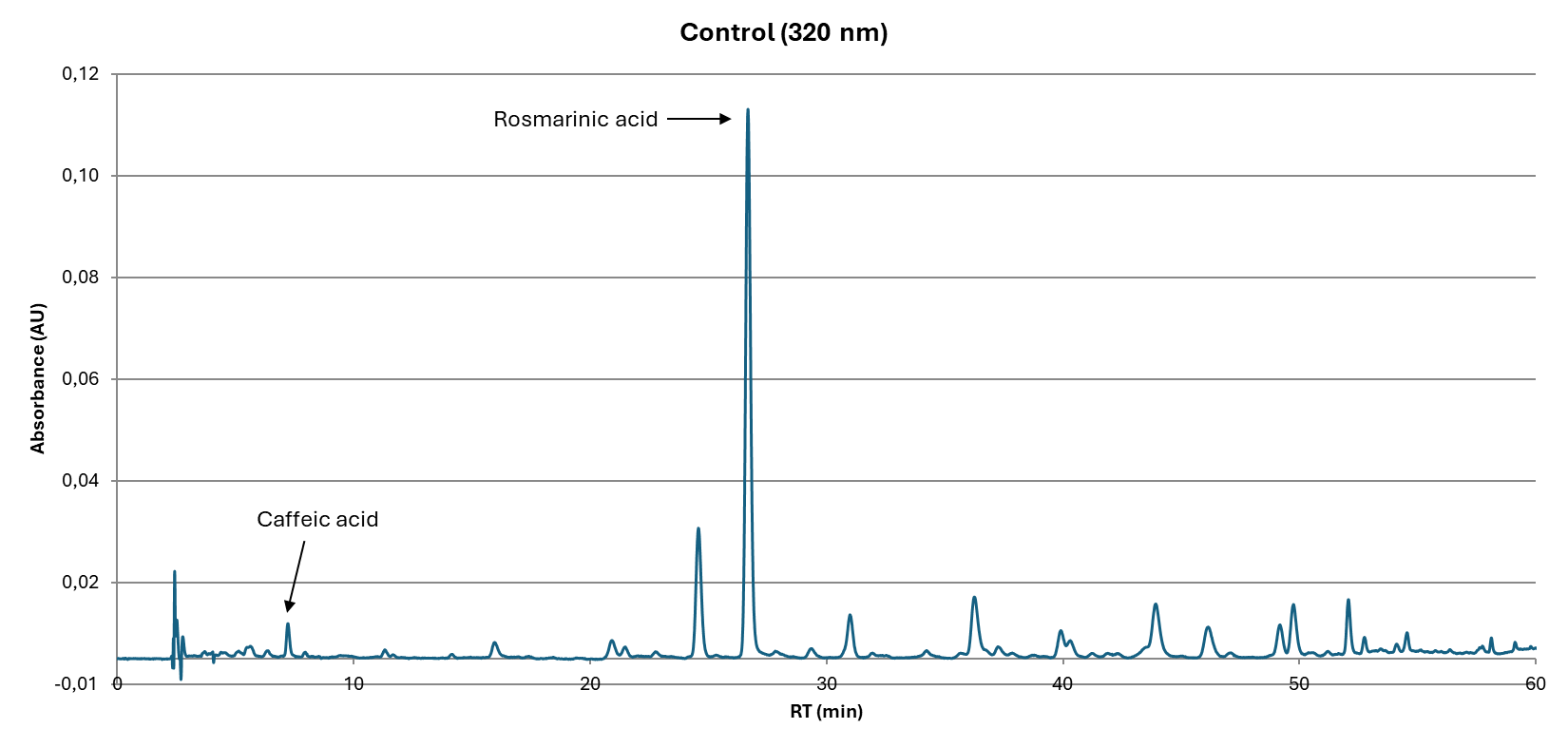** | | **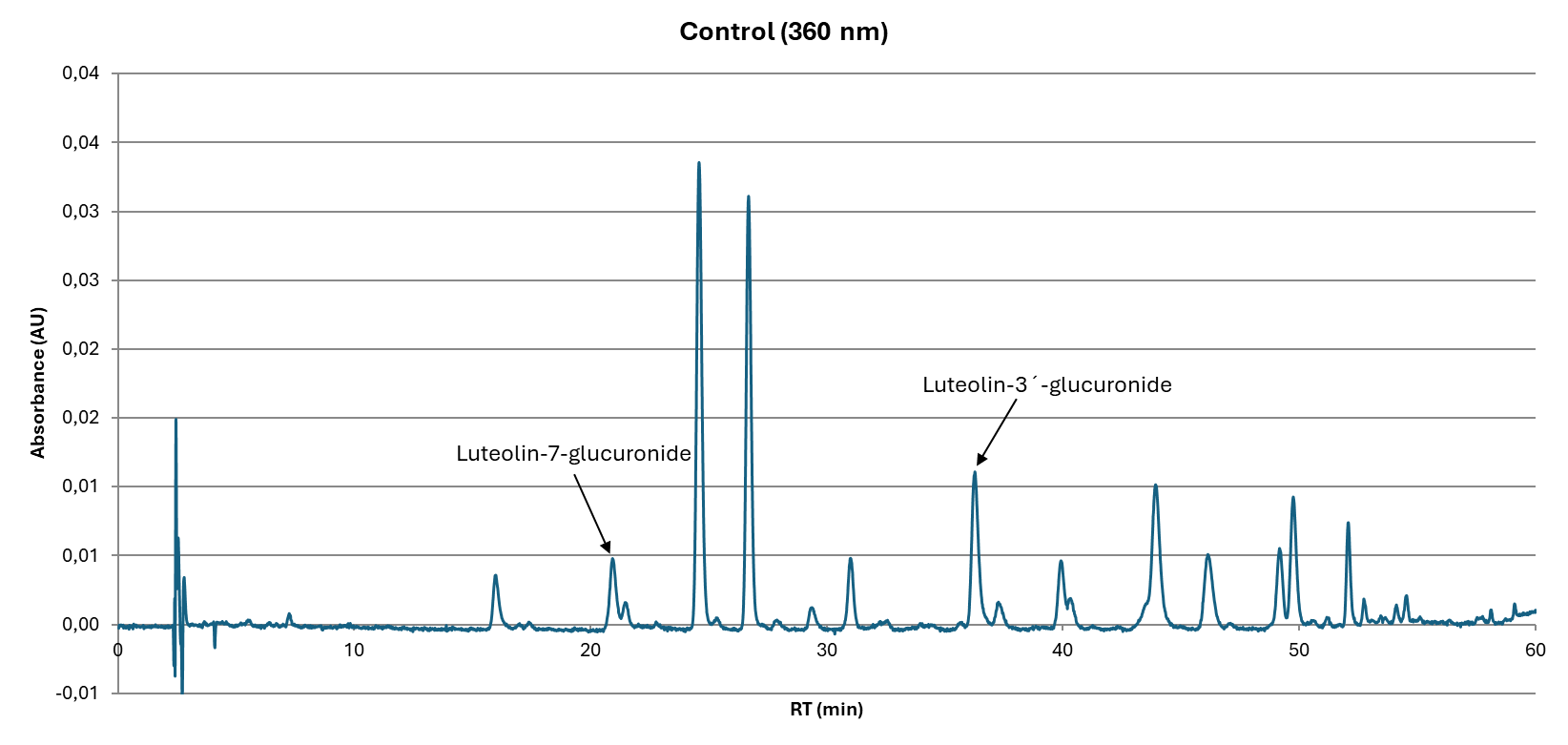** |
| --- | --- | --- | --- |
| **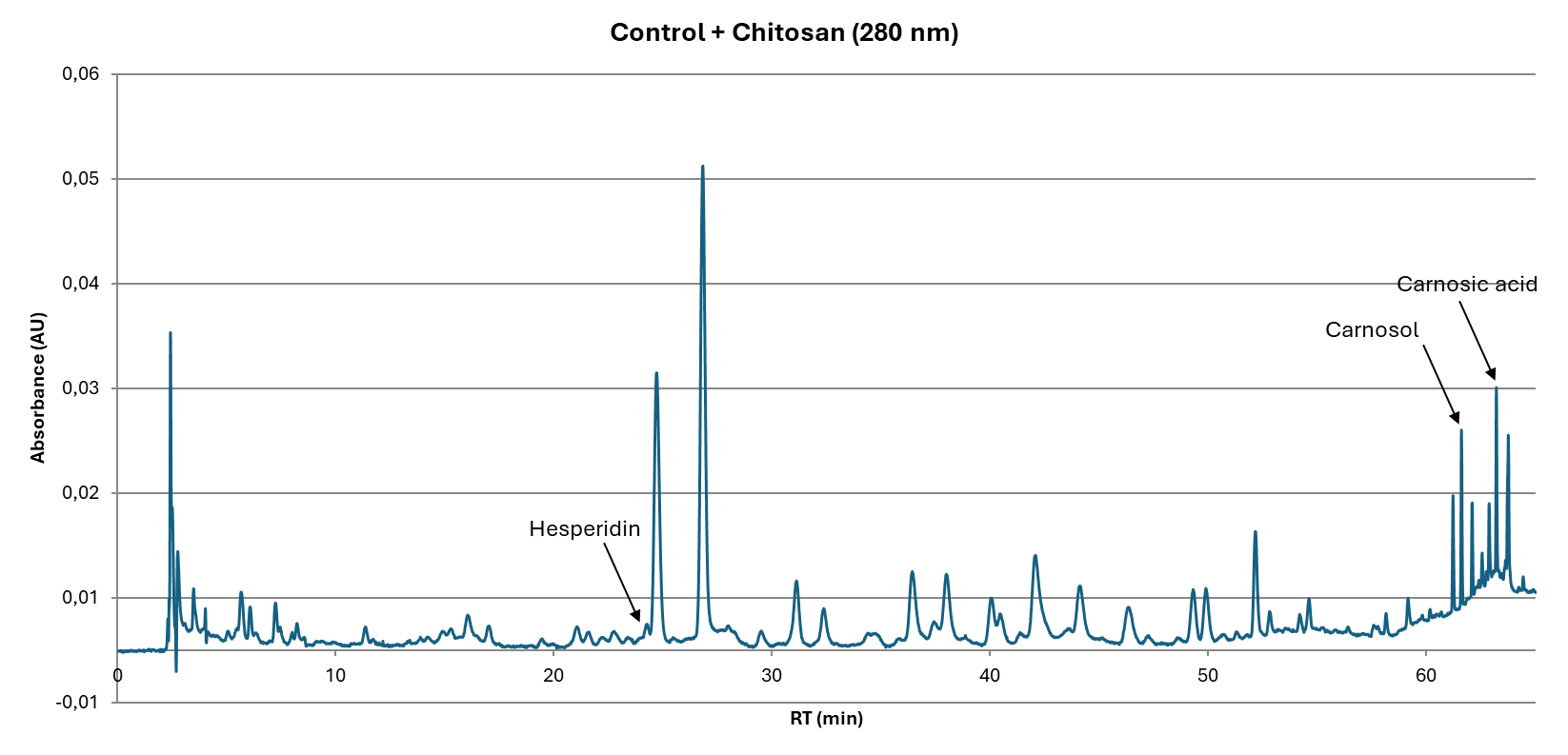** | **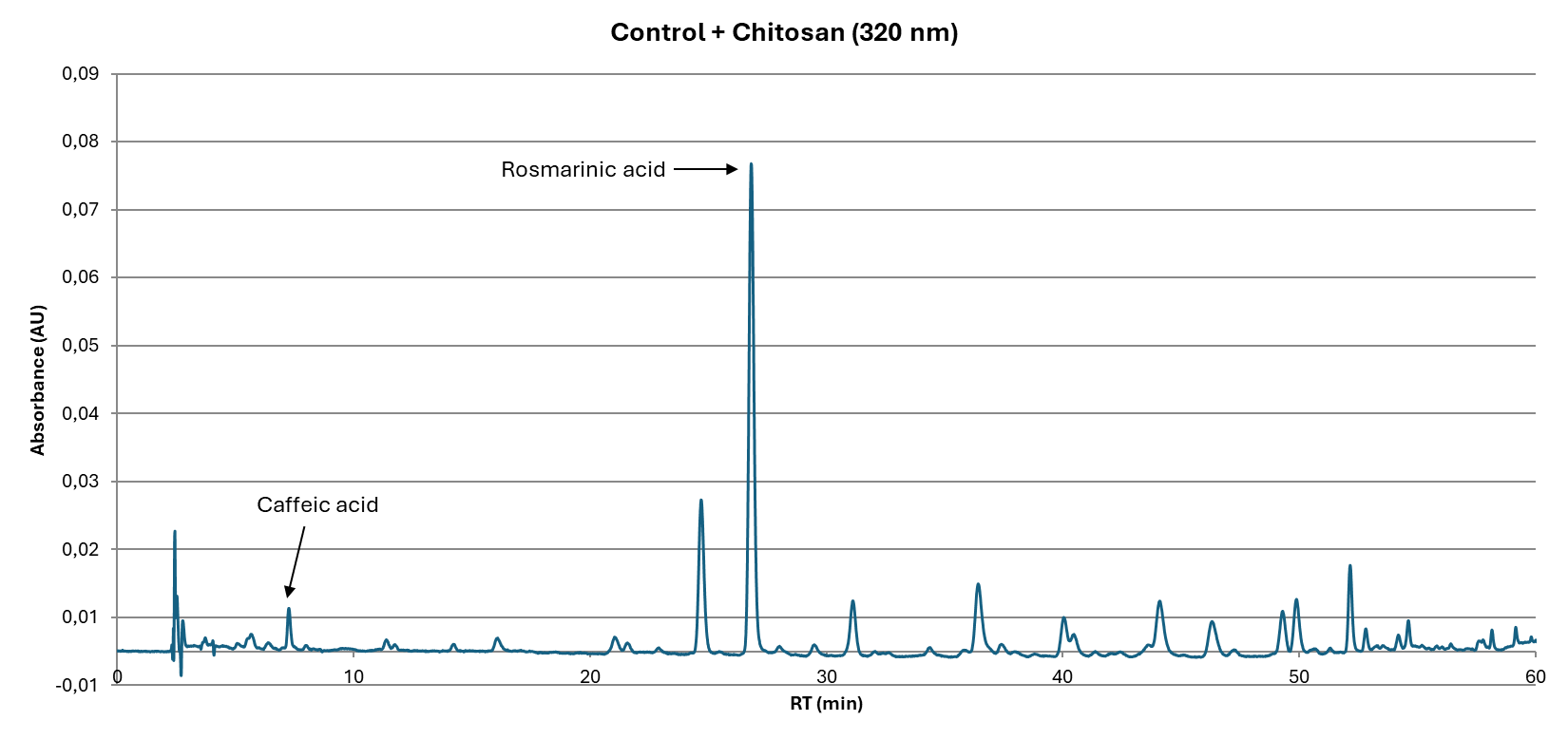** | | **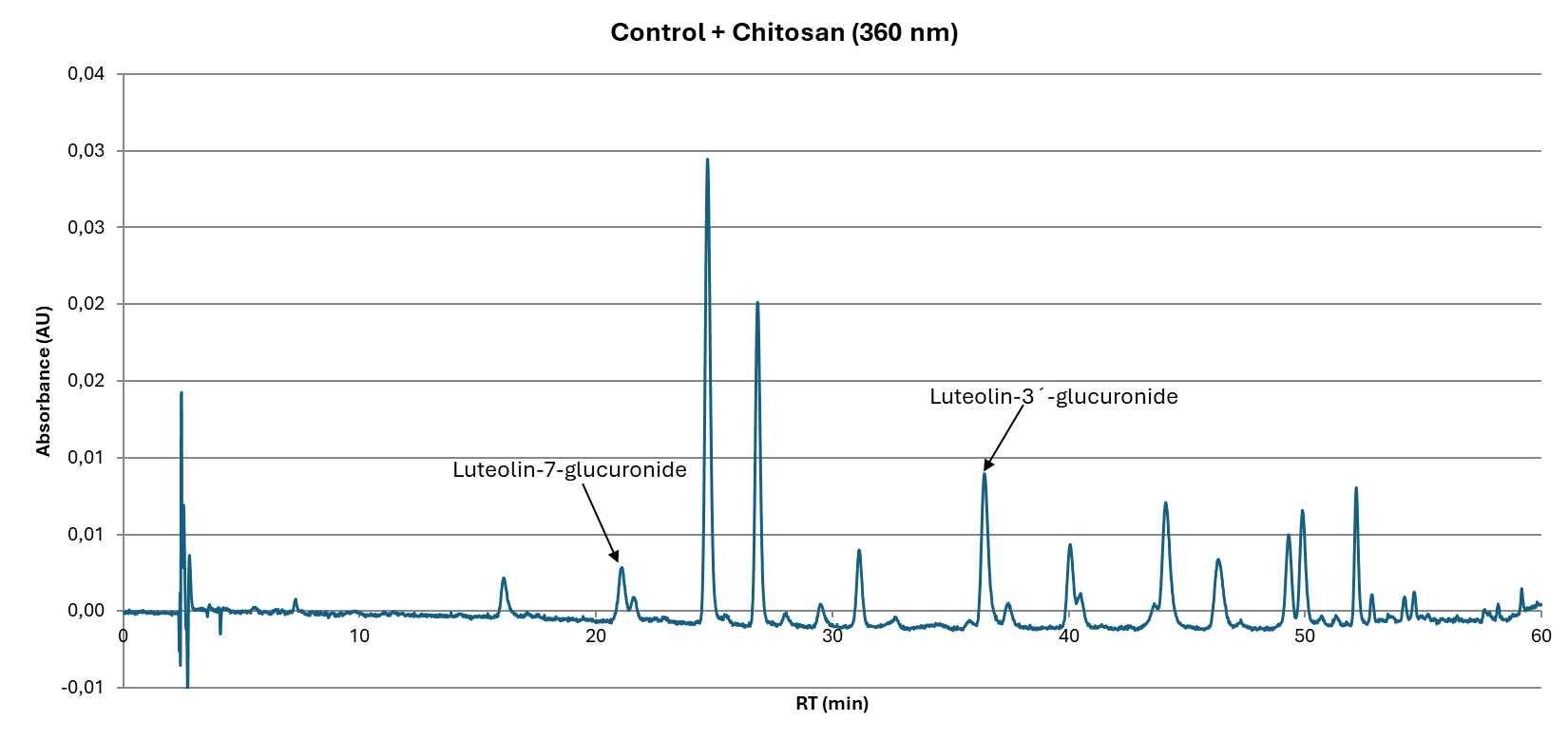** |
| **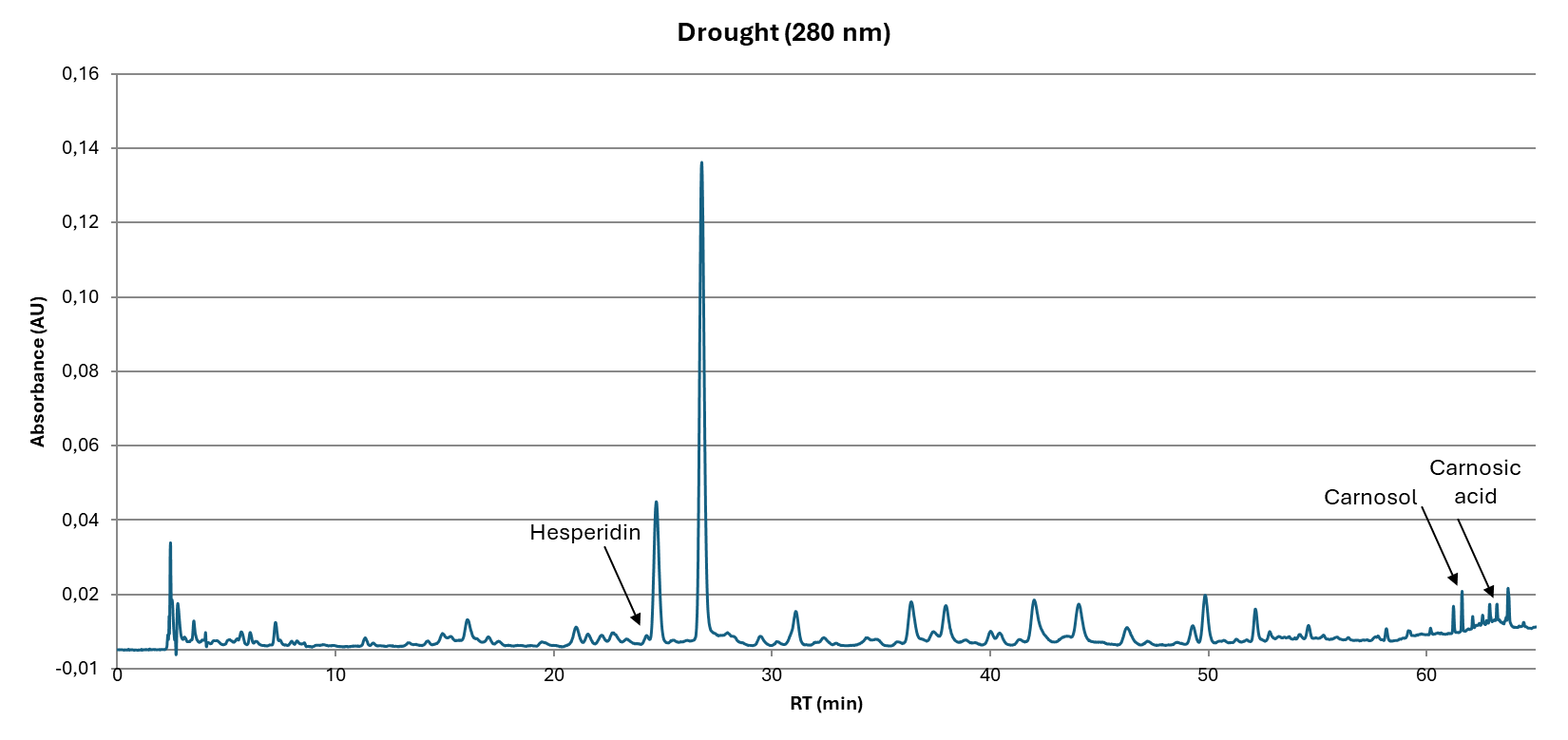** | **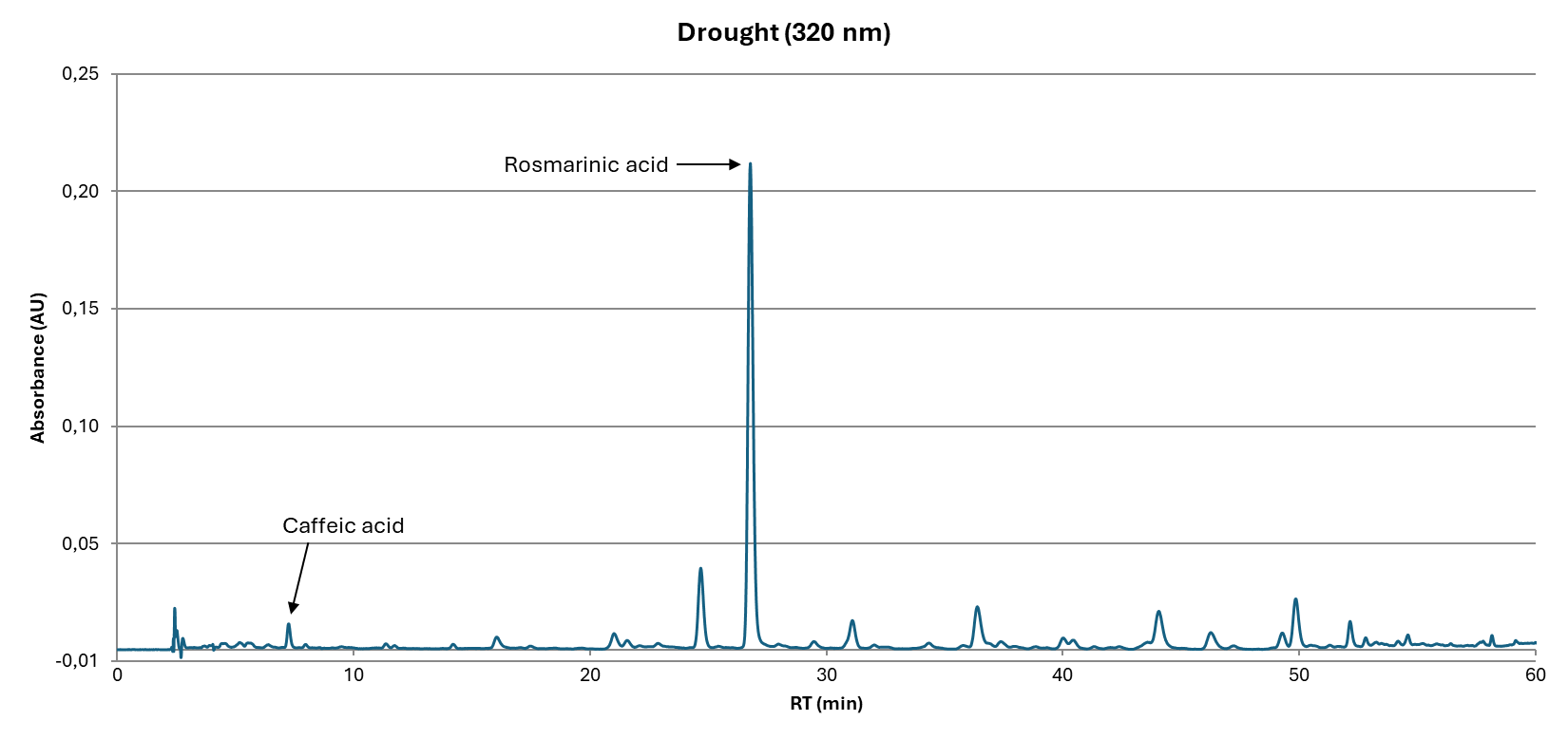** | | **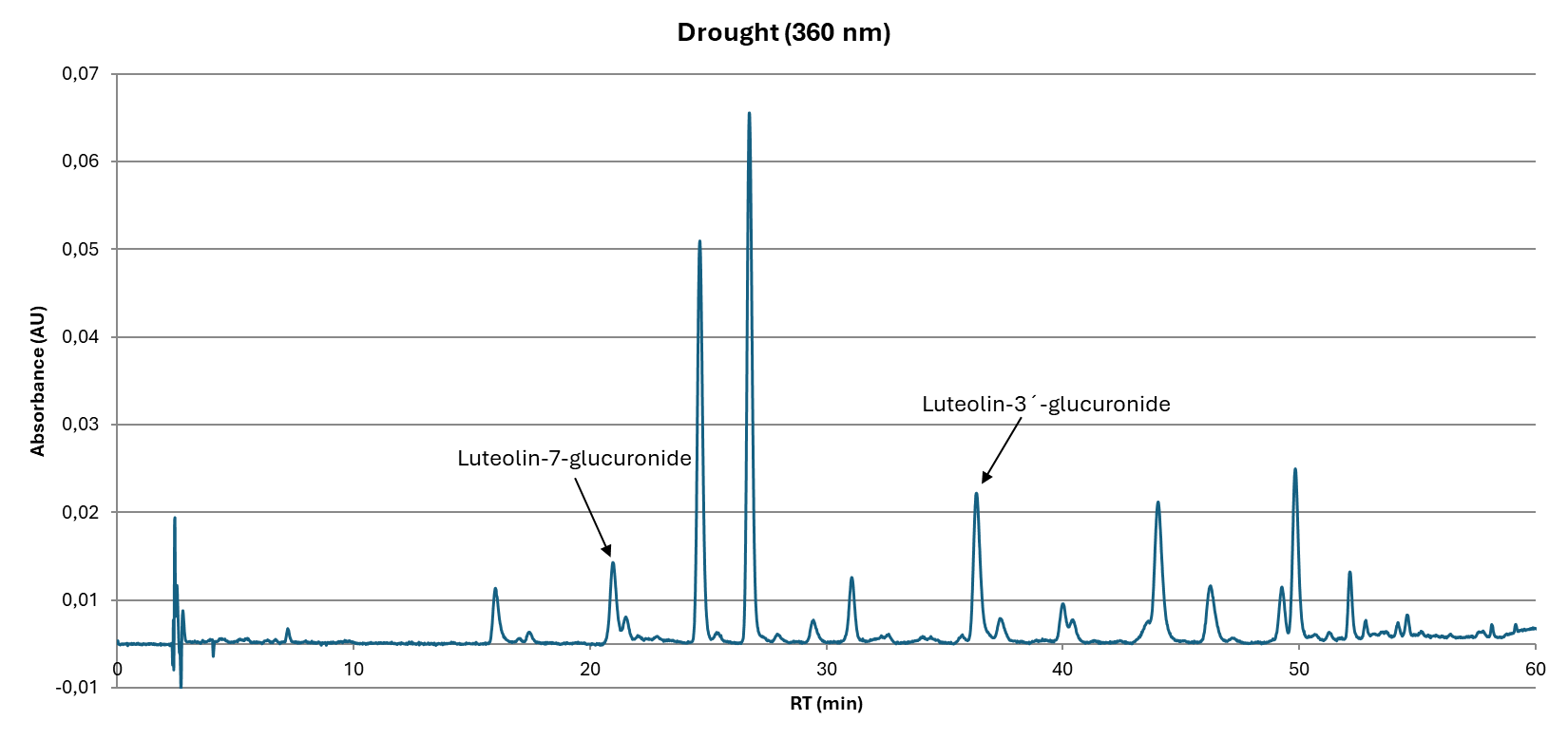** |
| **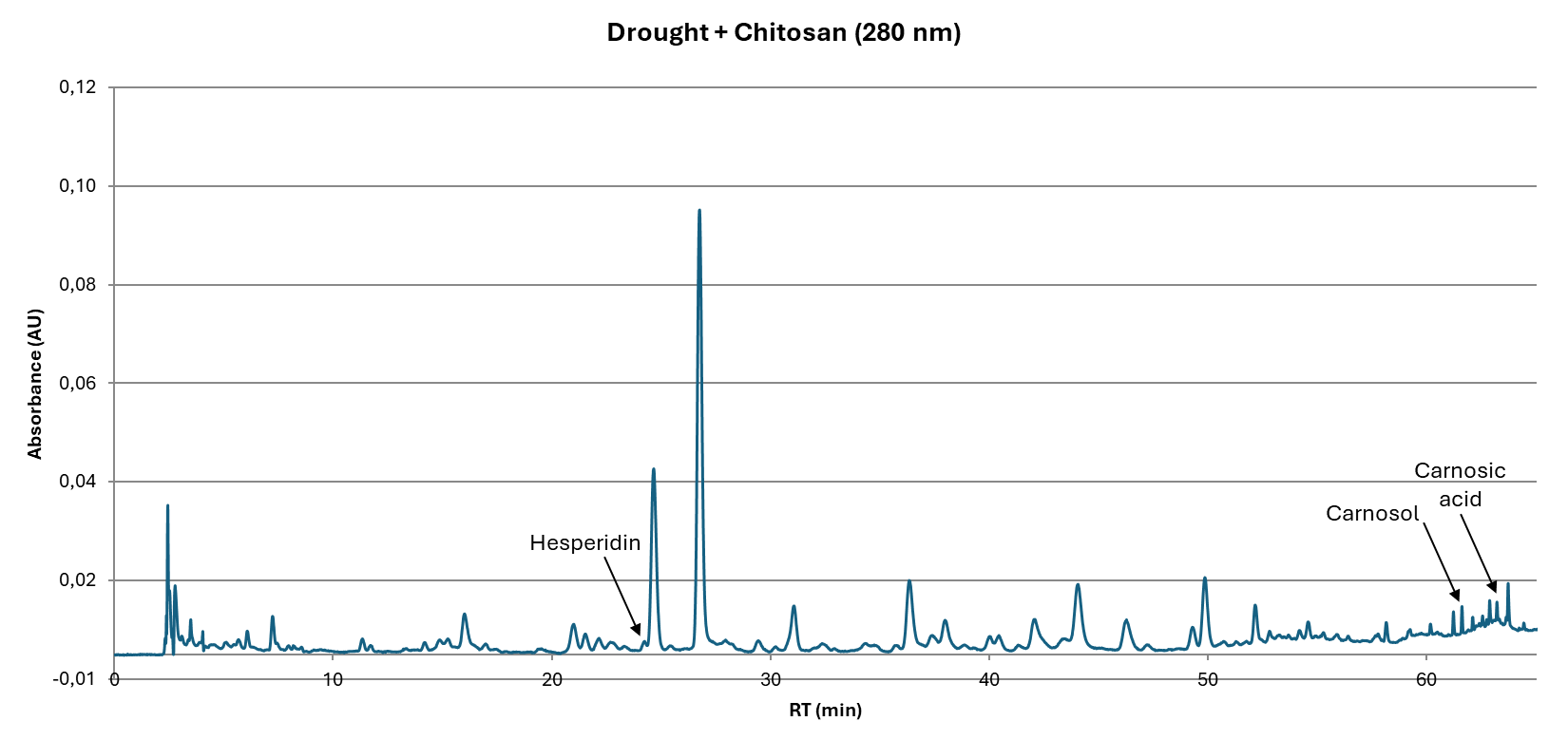** | **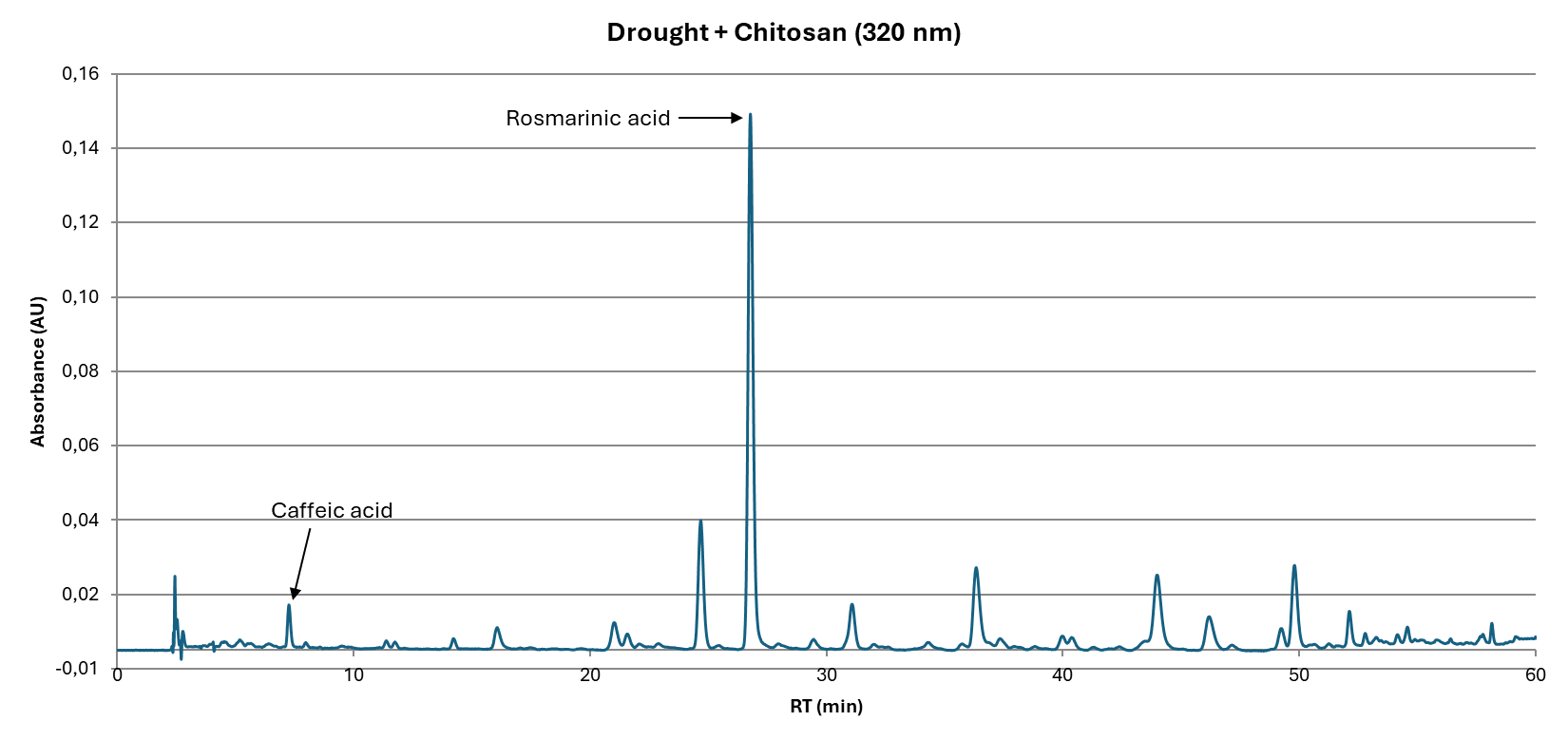** | | **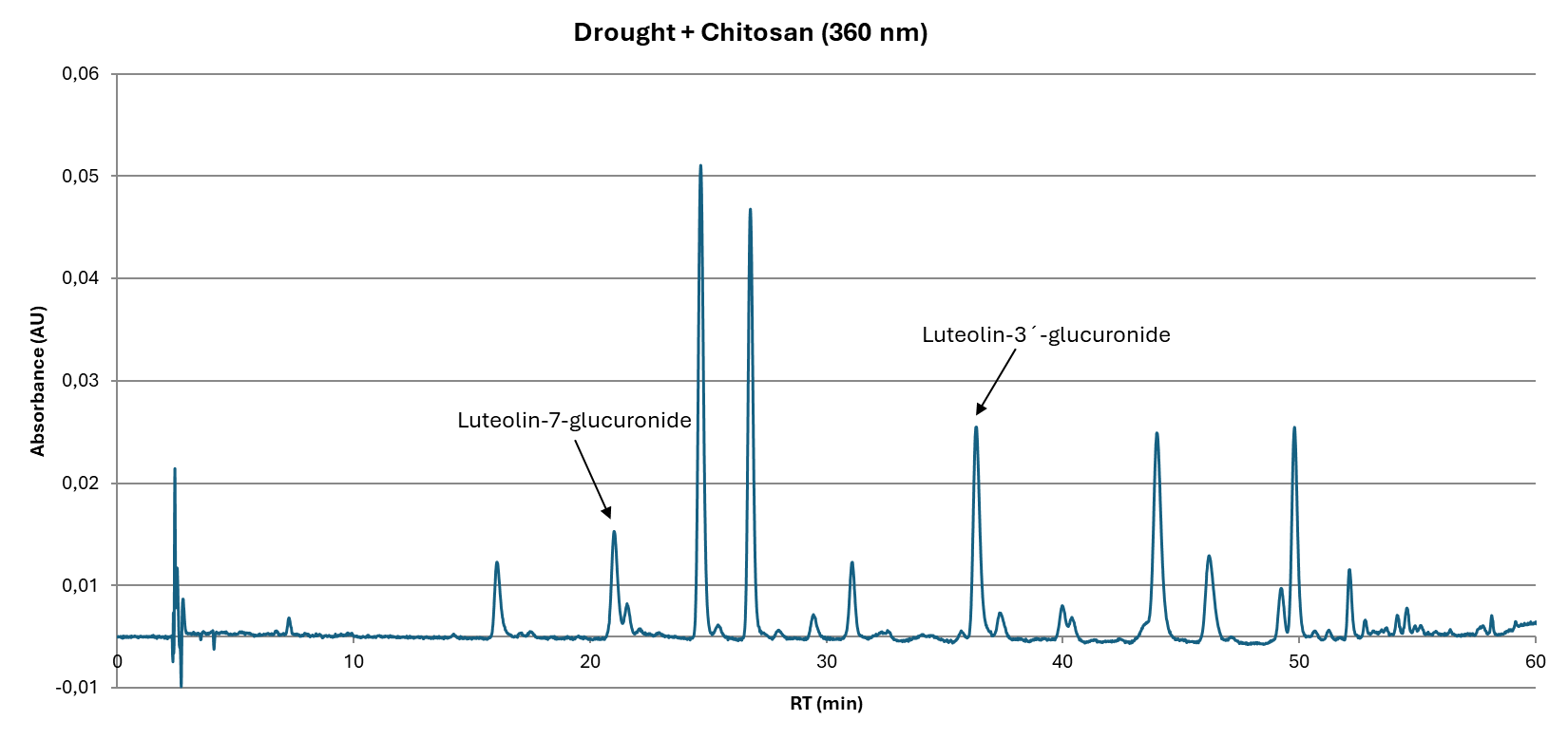** |
| **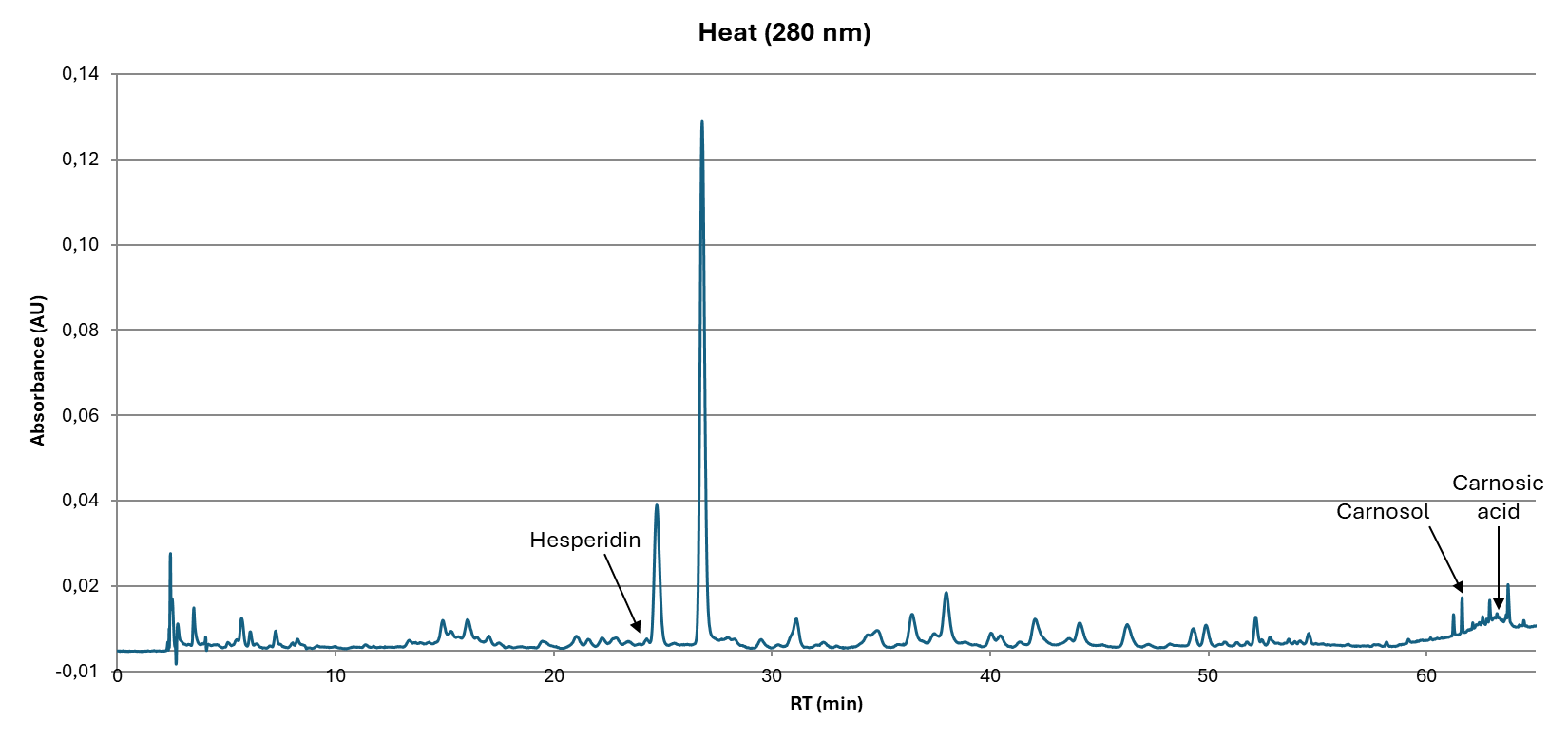** | **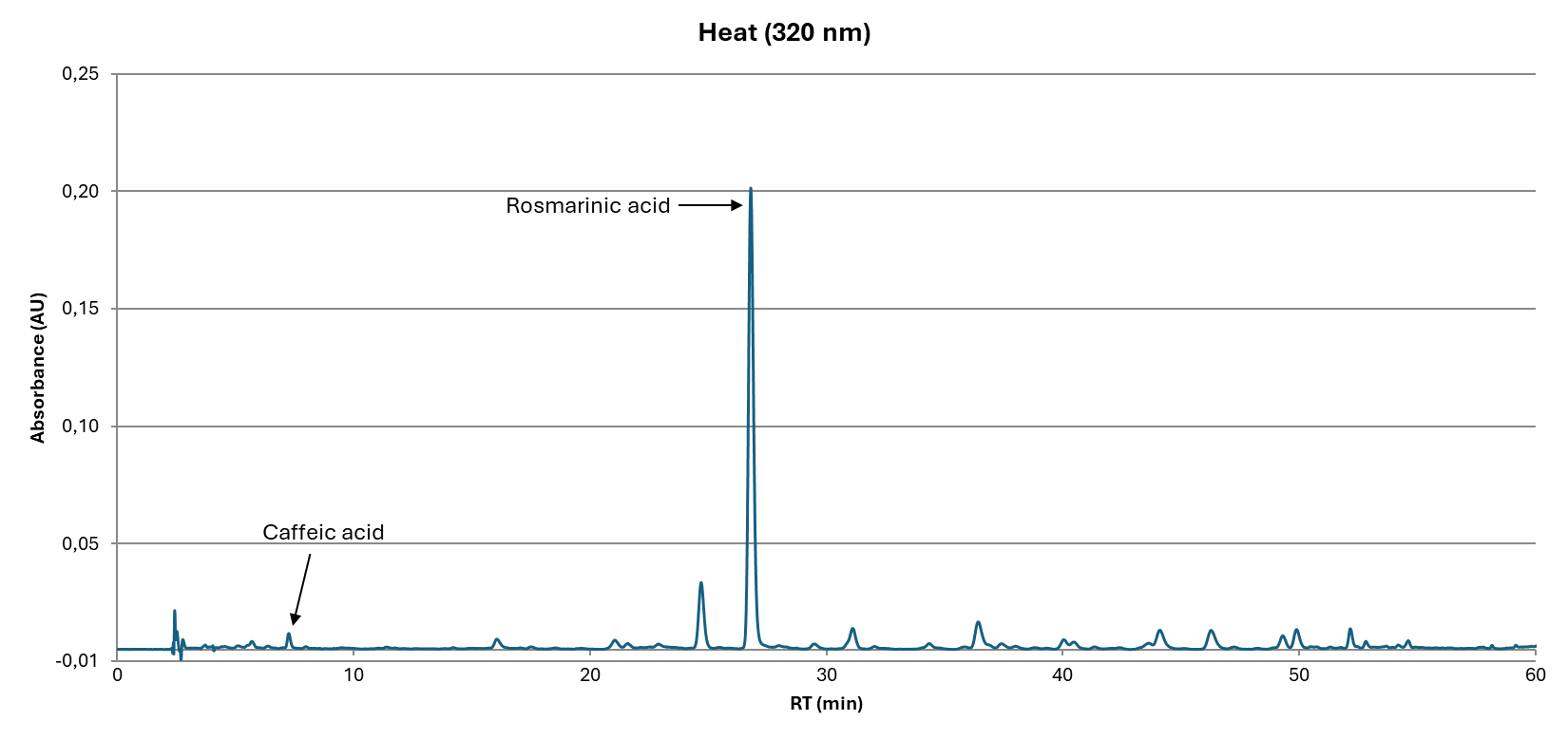** | **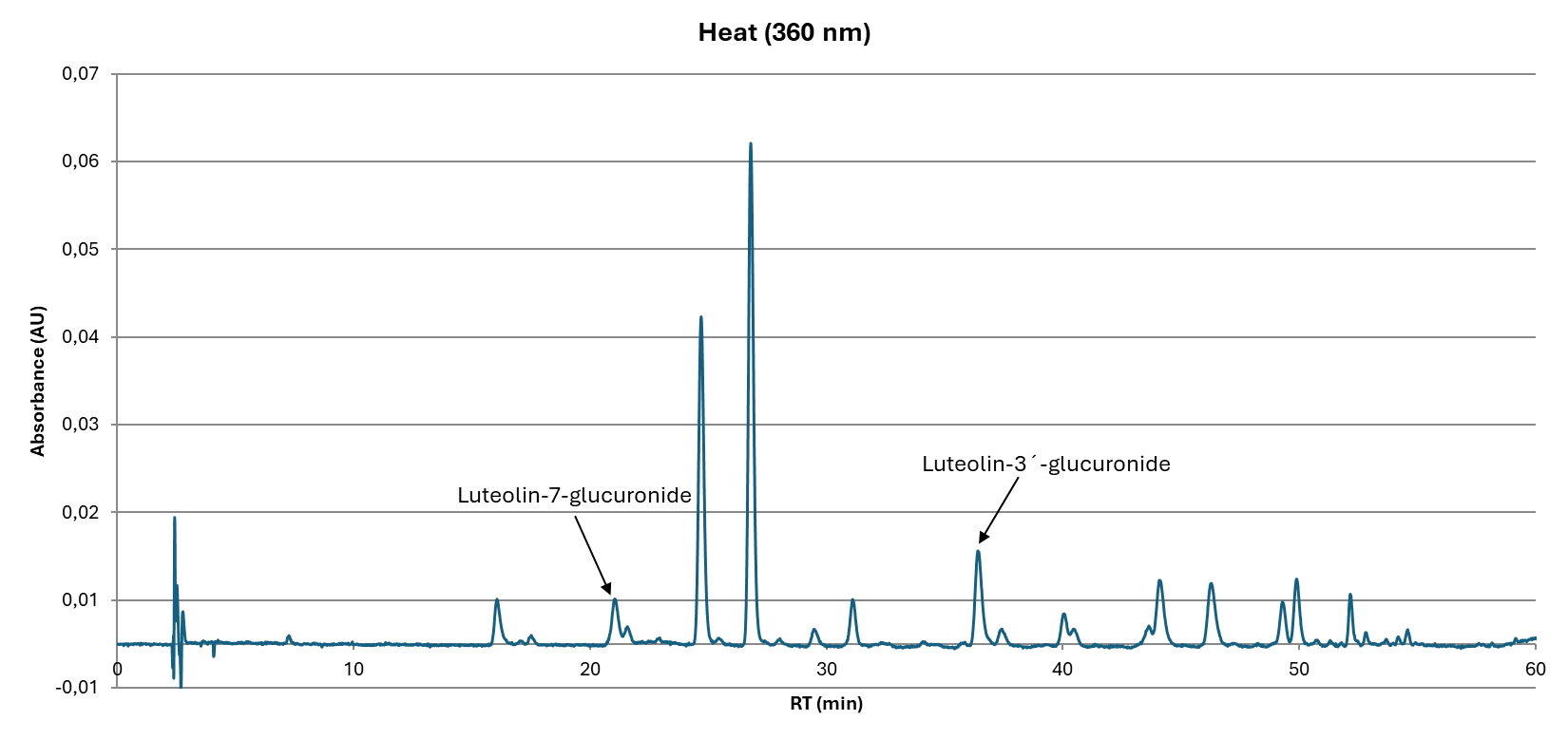** | |
| **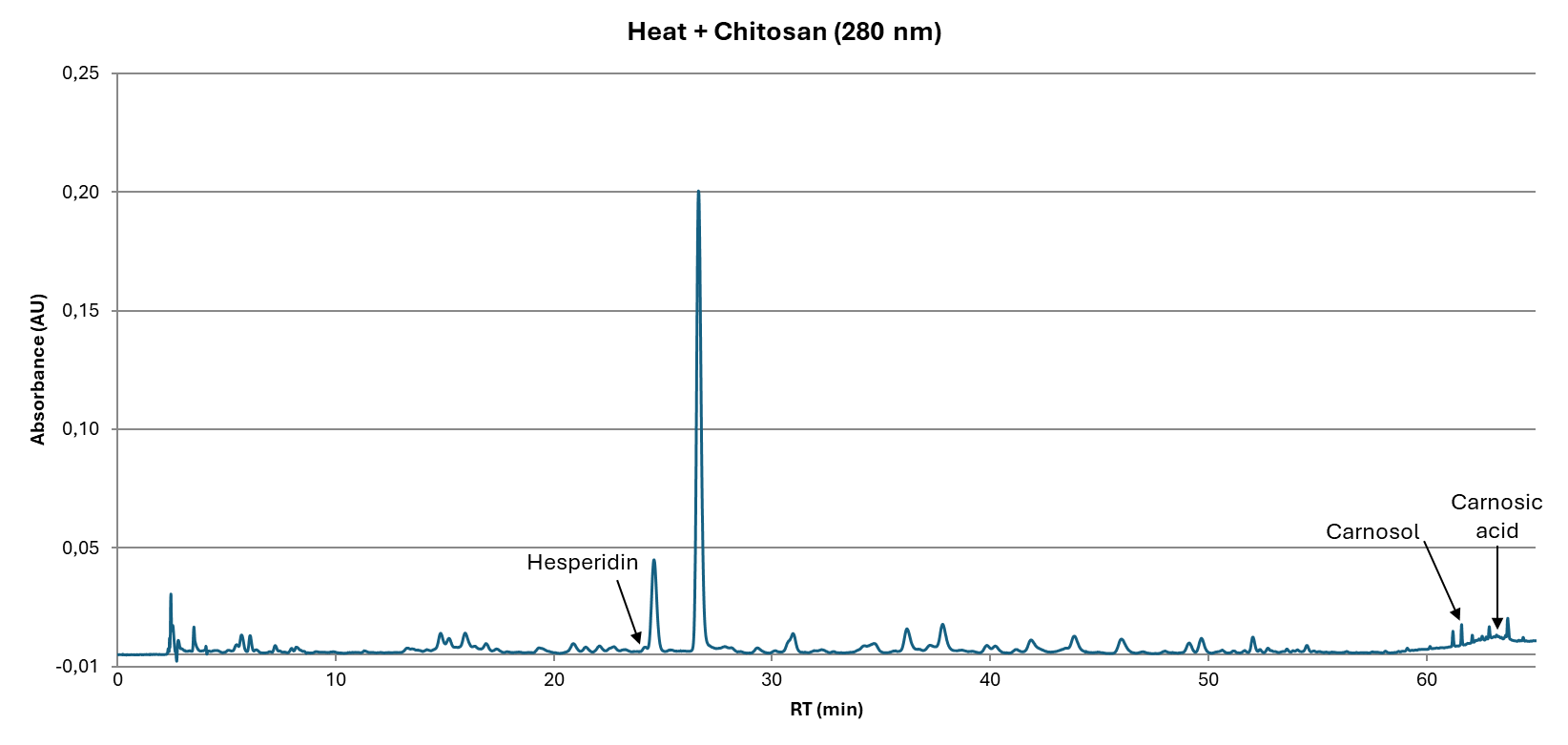** | **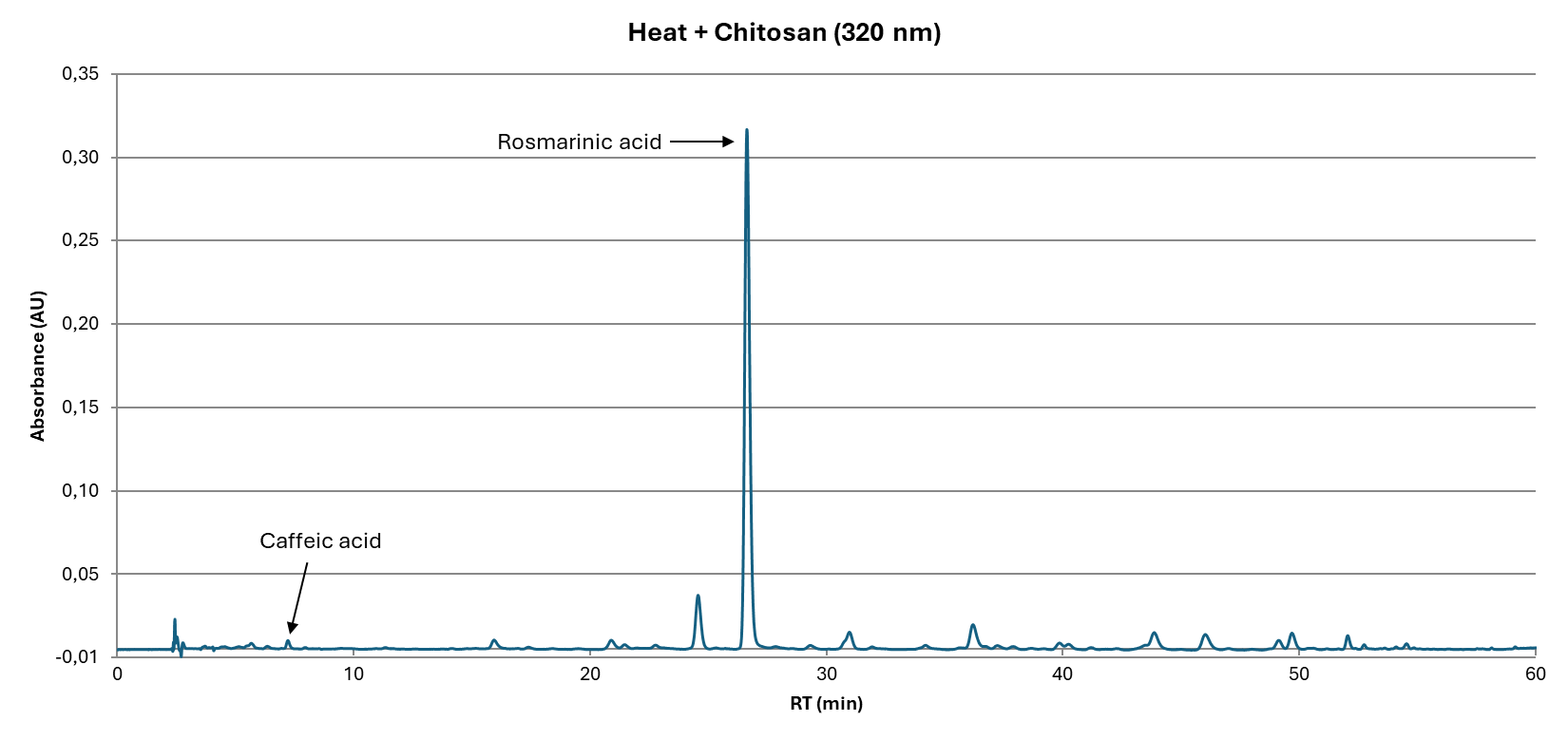** | **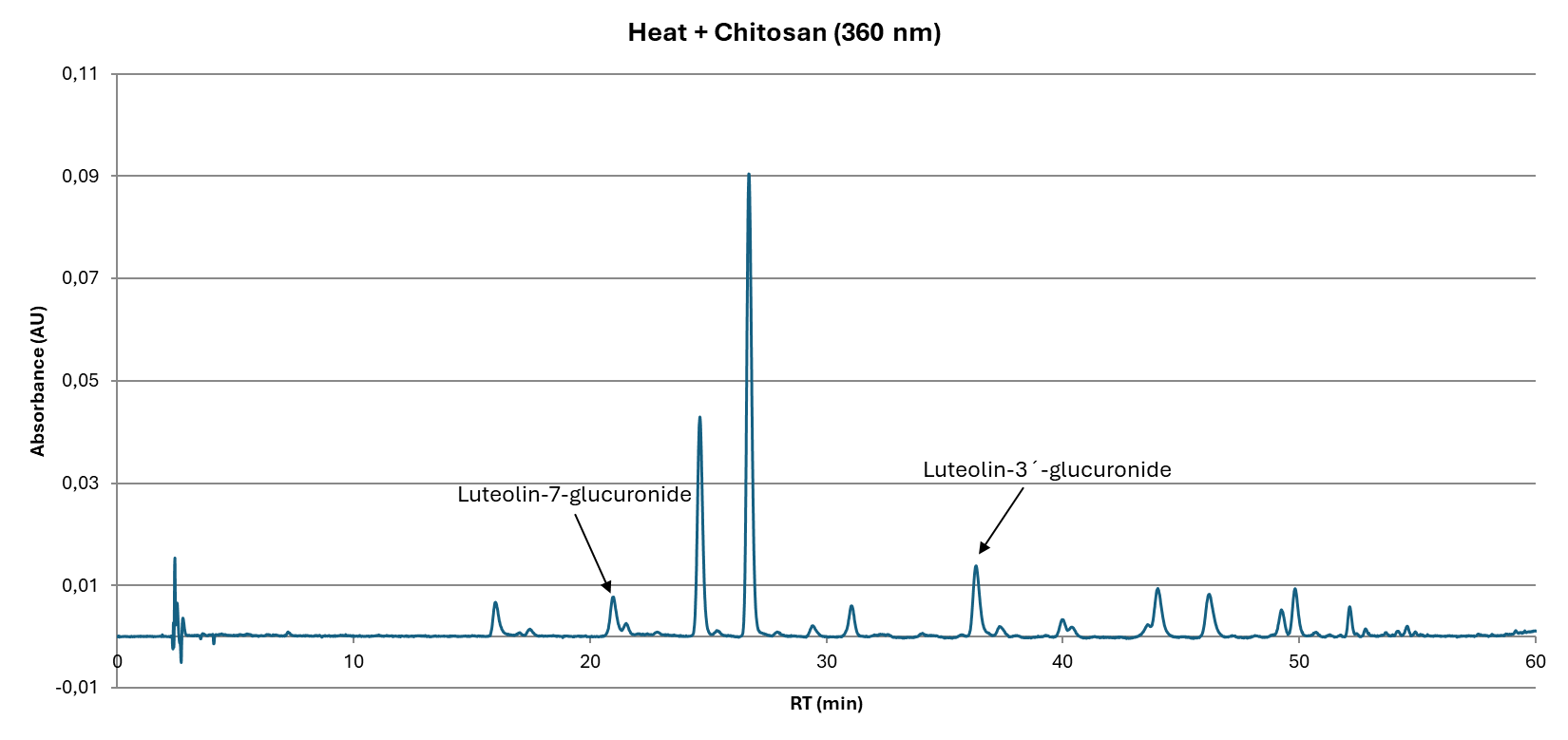** | |
| **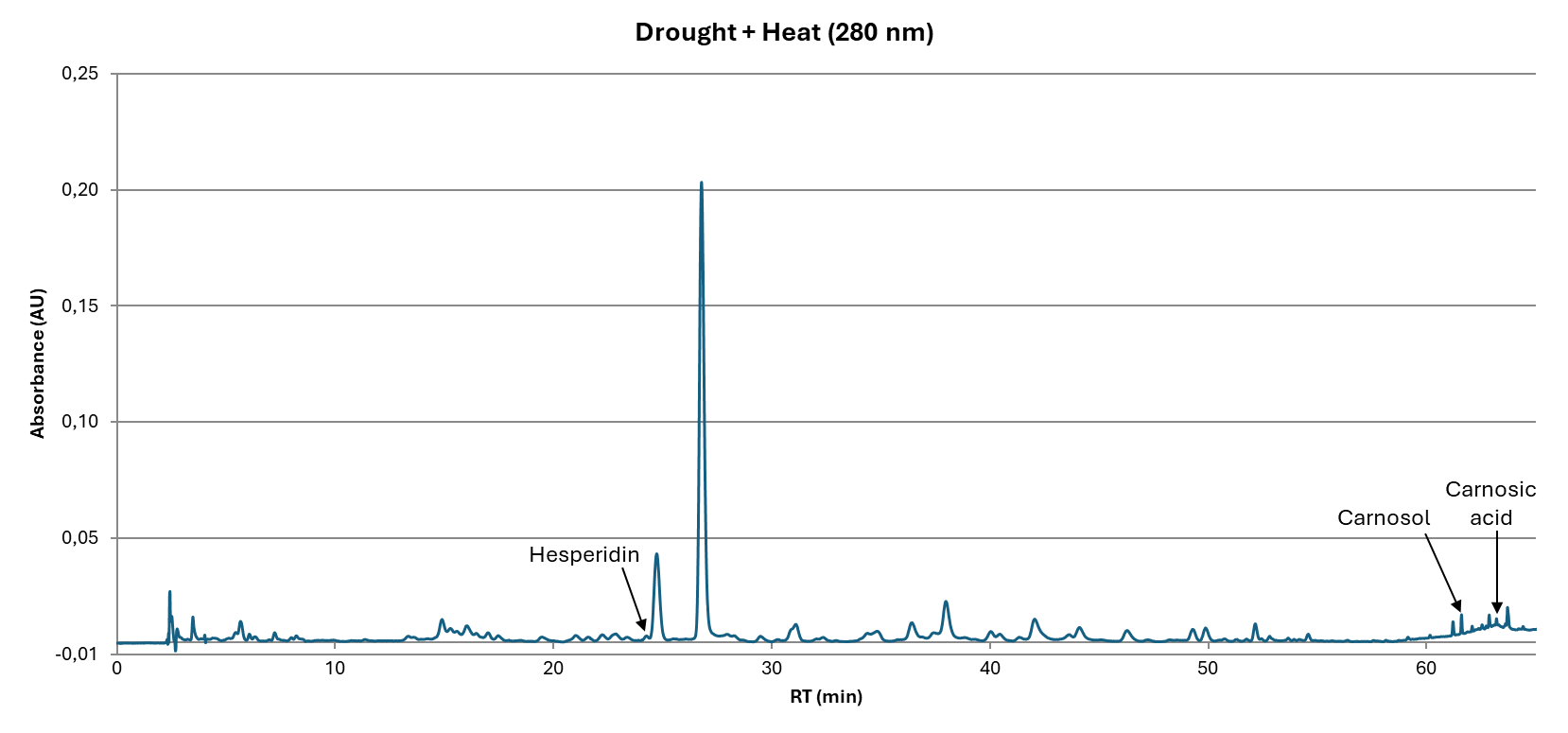** | **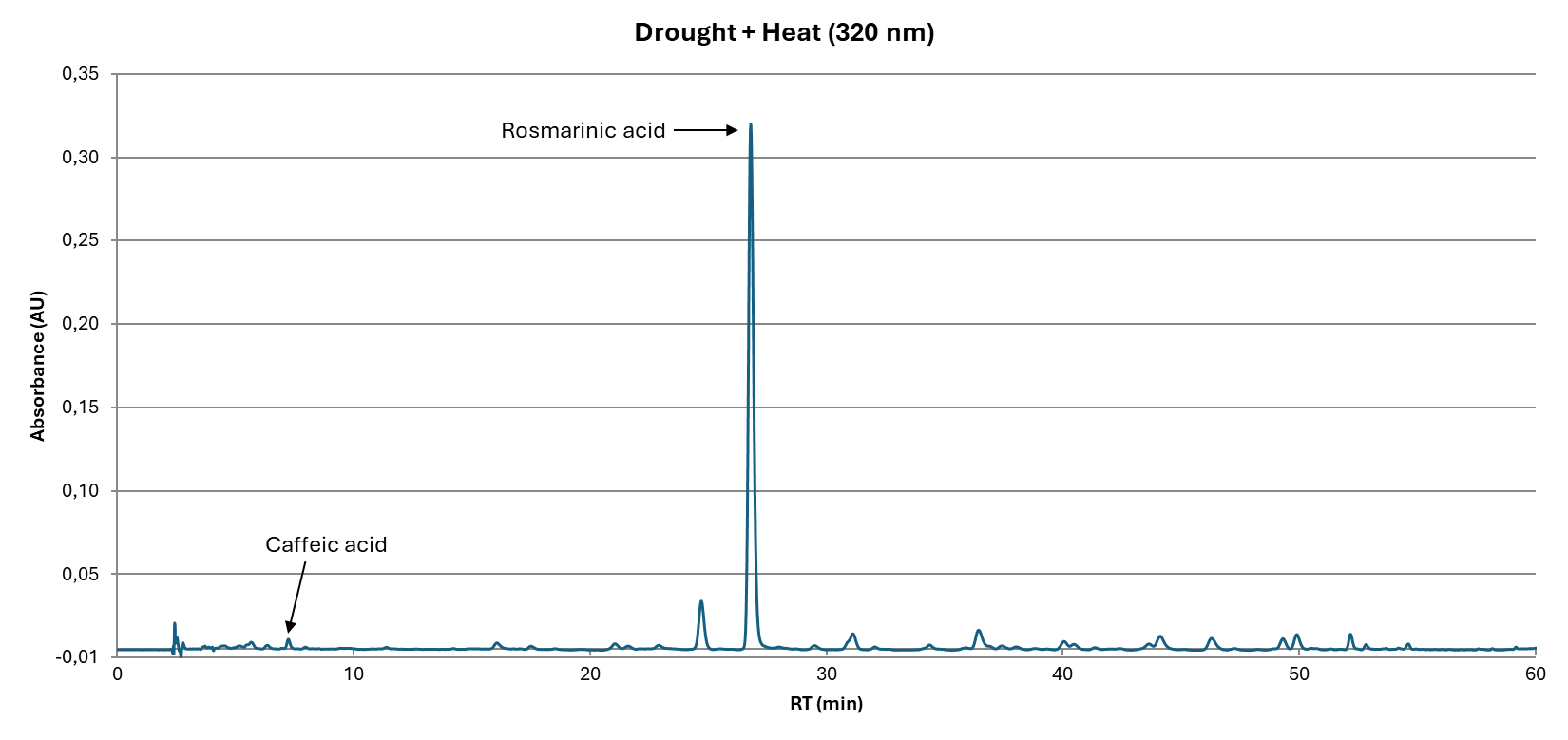** | **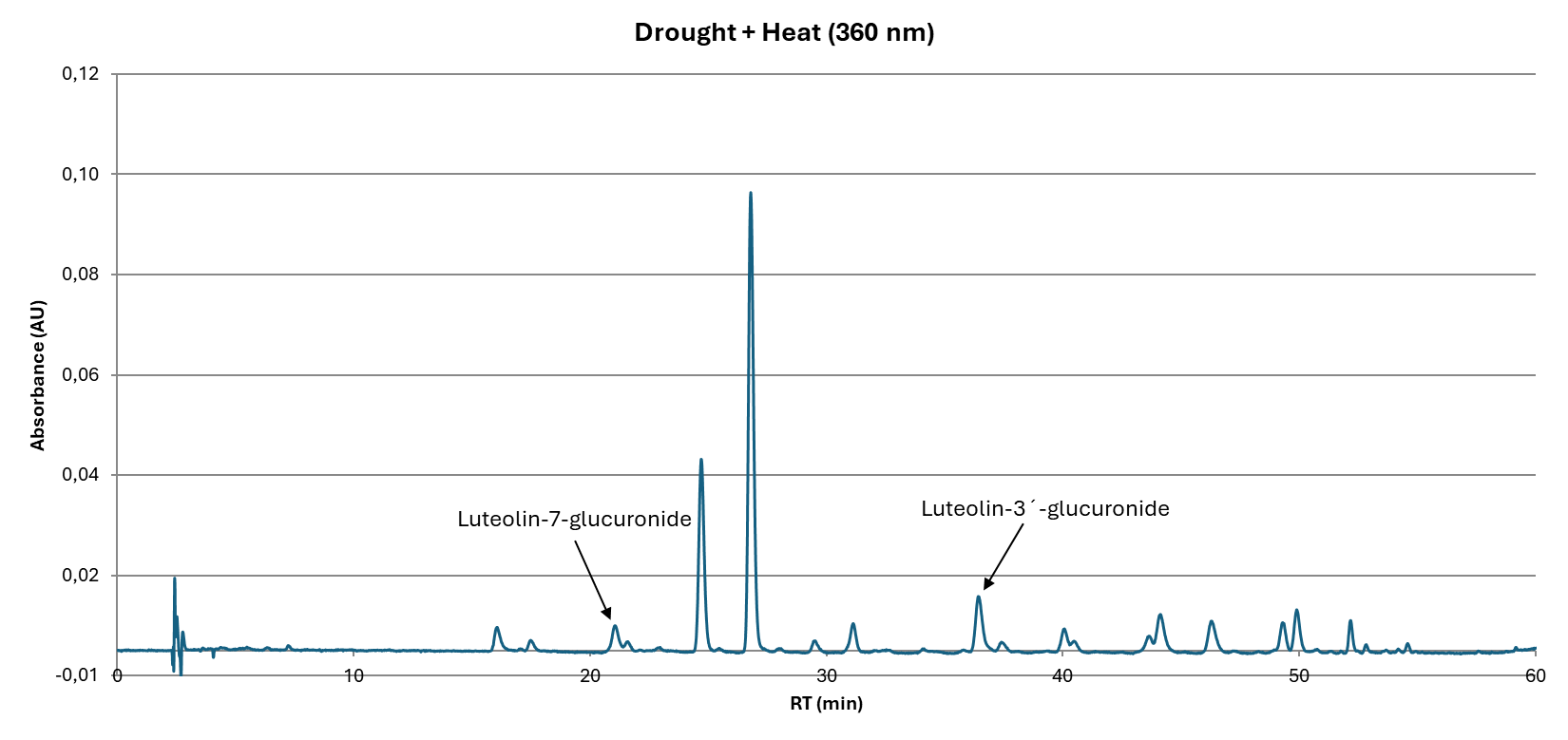** | |
| **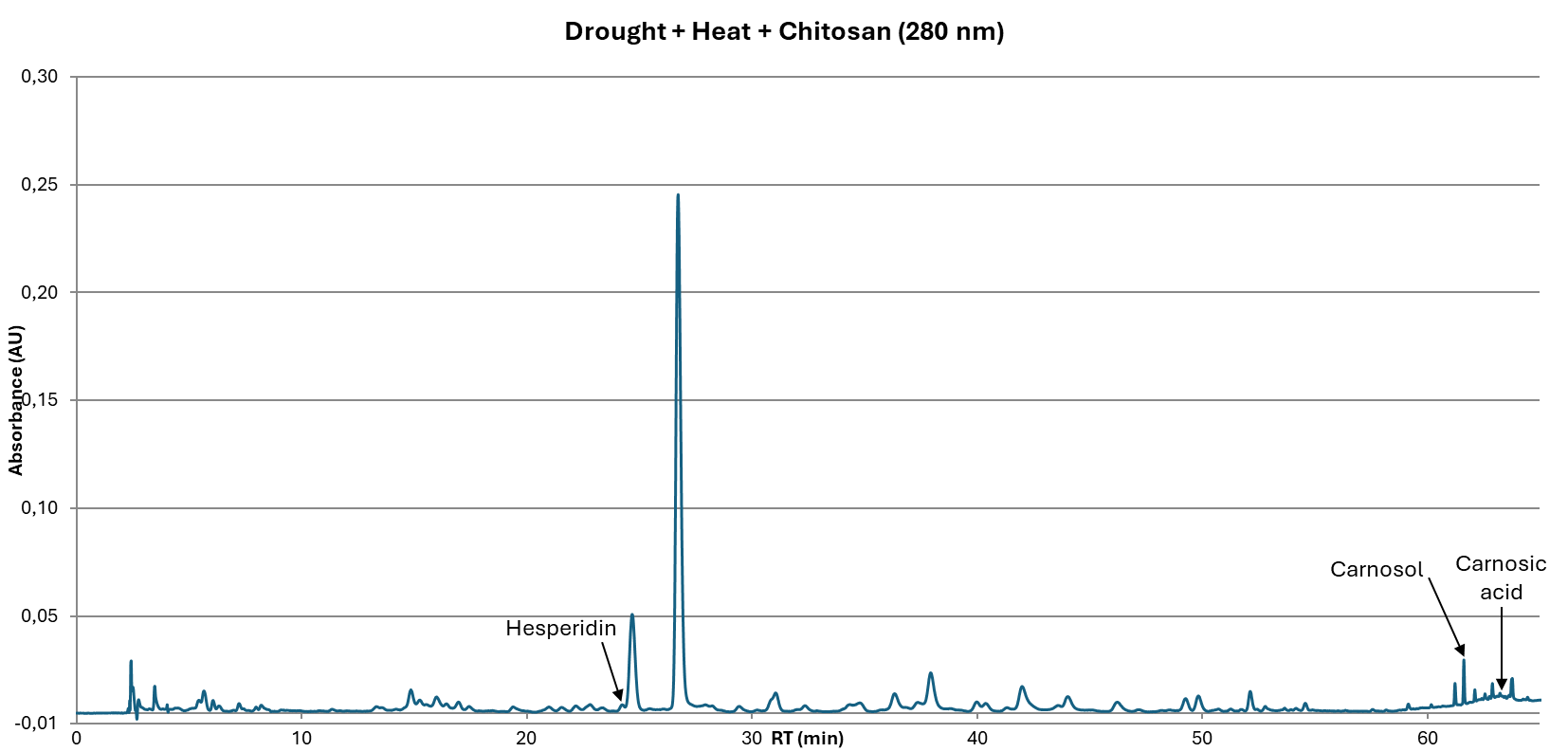** | **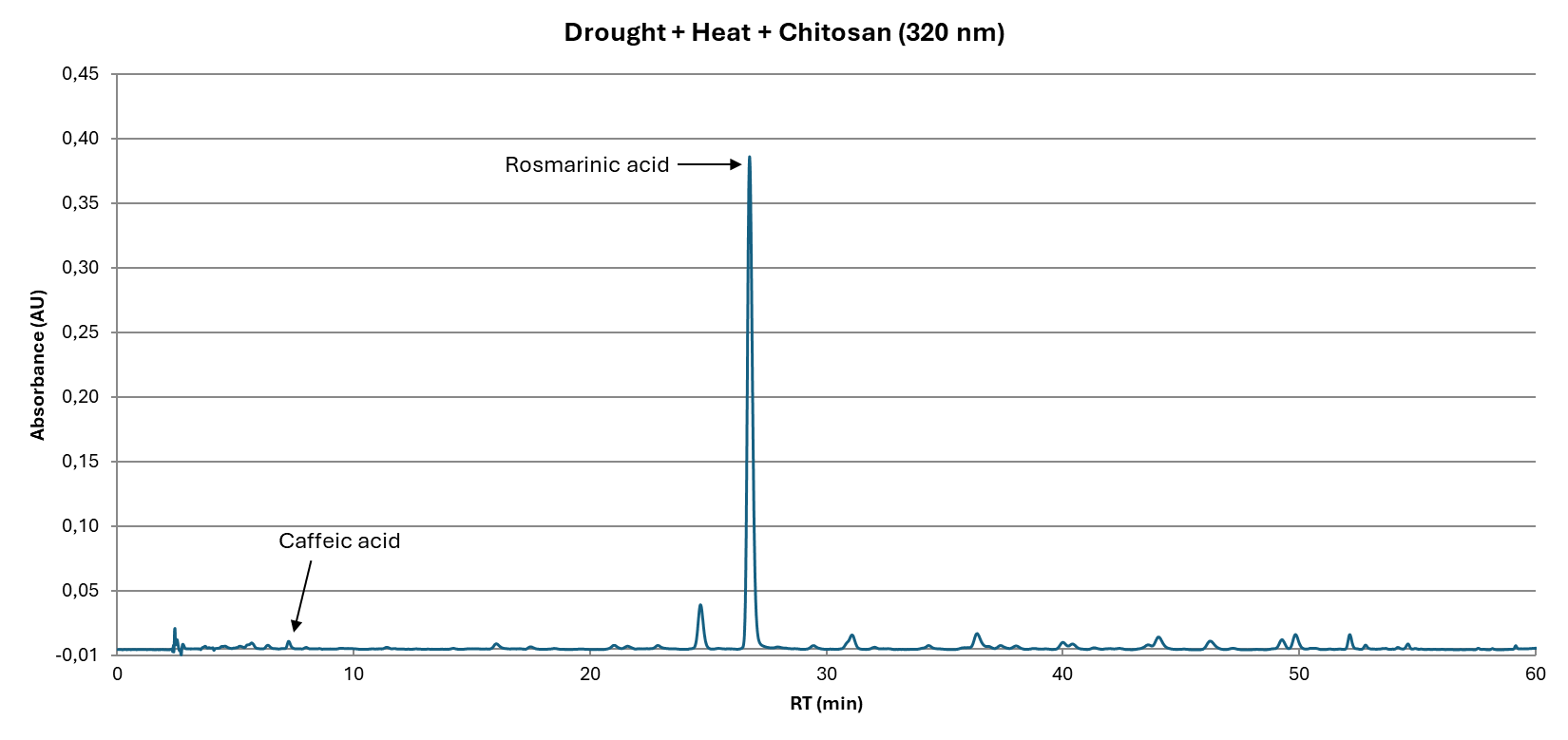** | **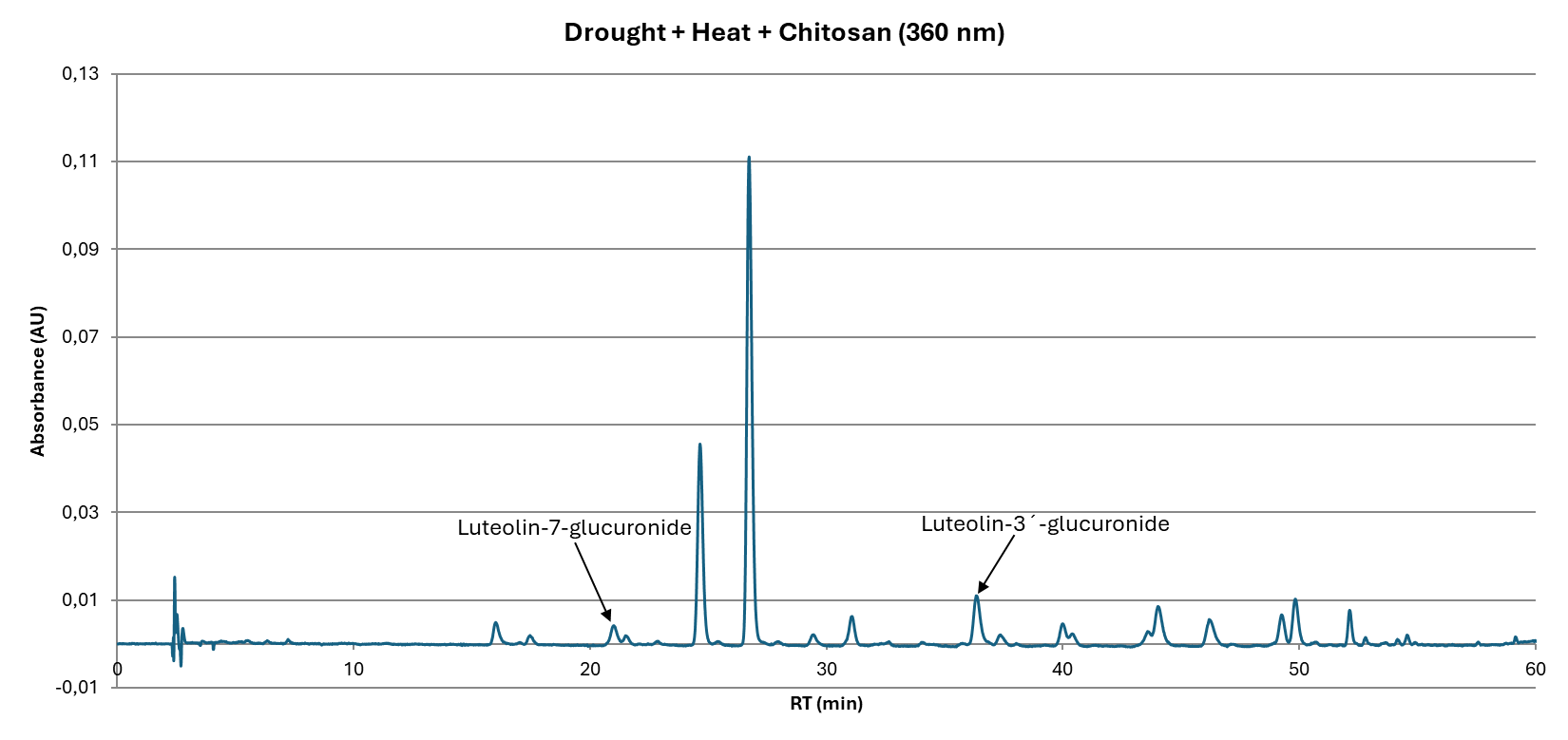** | |

**Figure S4.** GC-MS chromatograms of *Salvia rosmarinus* samples subjected to drought stress, heat stress, their combination, and chitosan application. Major compounds are identified according to Table S3: 2 – α-pinene (RT 6.67); 3 – camphene (RT 7.05); 13 – 1,8-cineole (RT 9.71); 19 – linalool (RT 12.25); 21 – (+)-camphor (RT 13.55); 25 – endo-borneol (RT 14.49); 31 – l-verbenone (RT 15.90); 35 – bornyl acetate (RT 18.44); 41 – caryophyllene (RT 22.68).

| 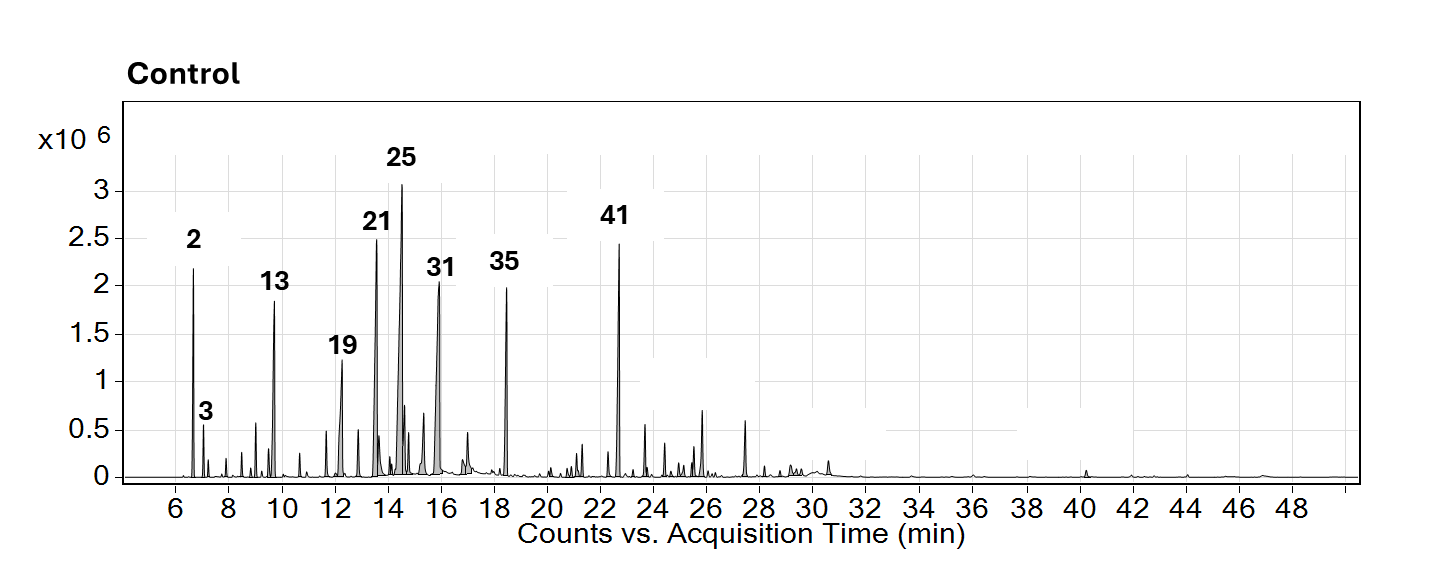 | **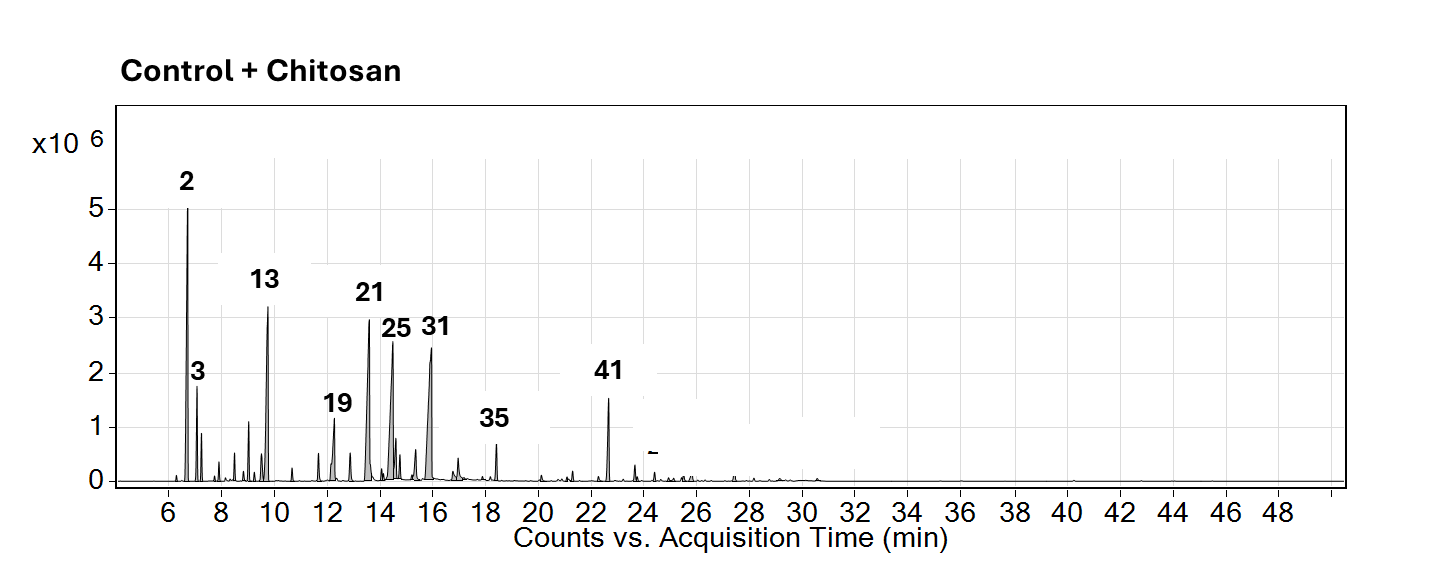** |
| --- | --- |
| 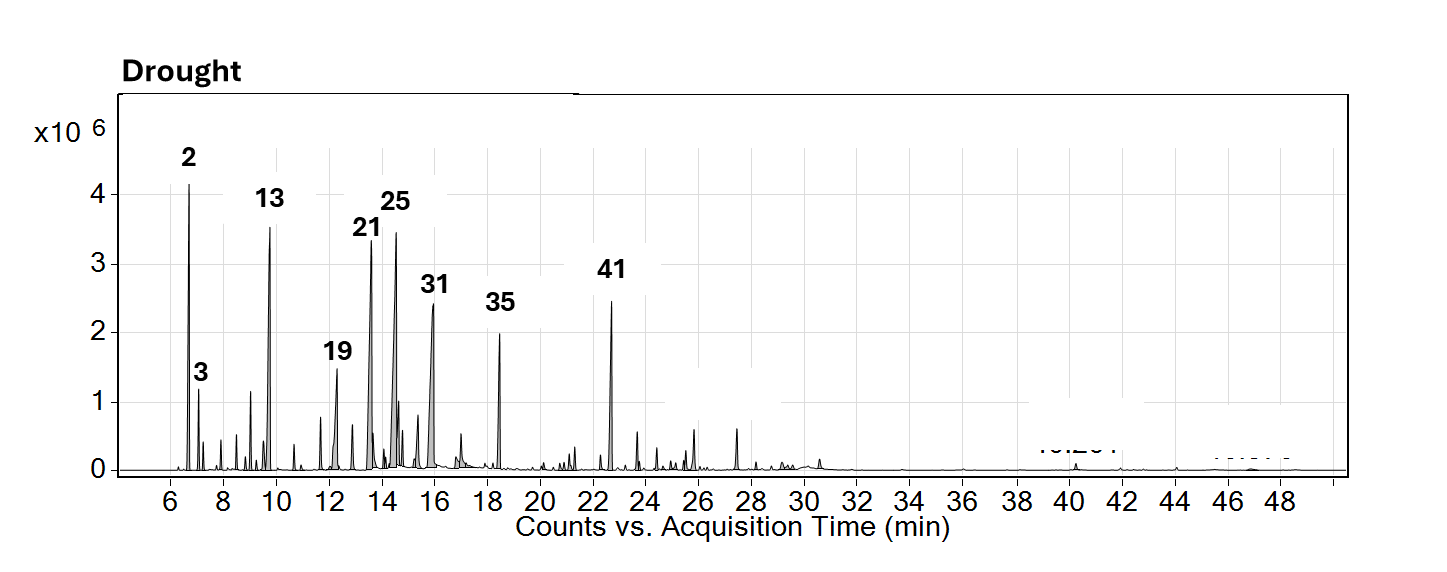 | **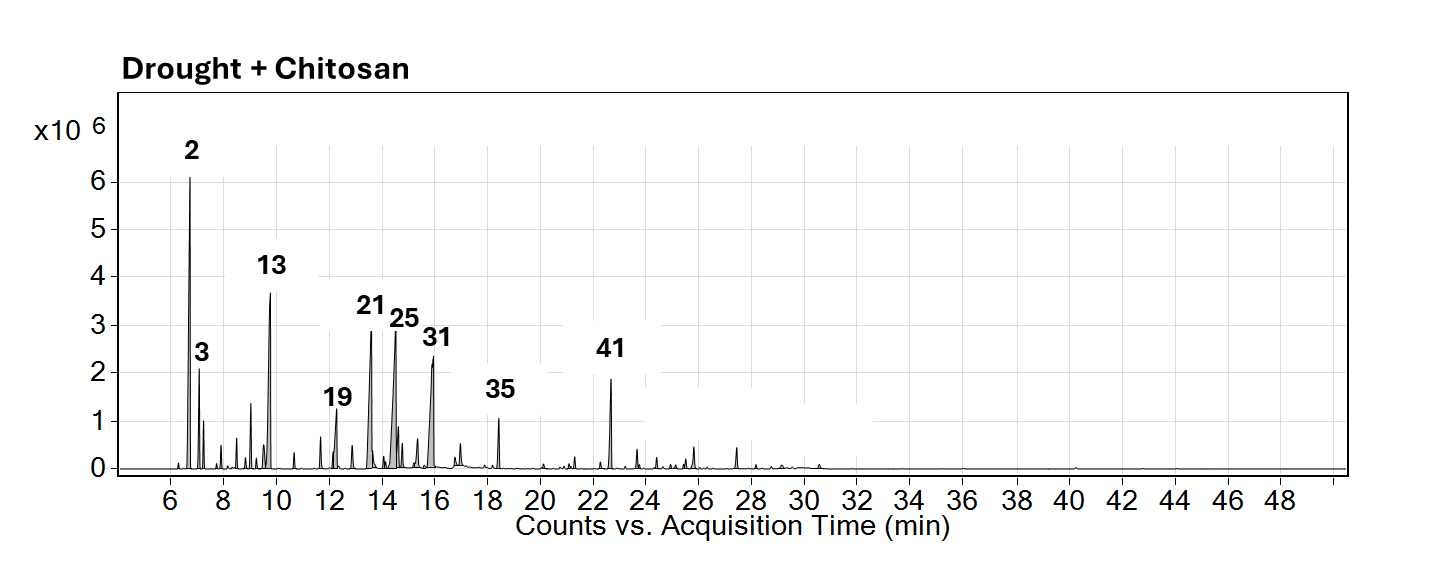** |
| 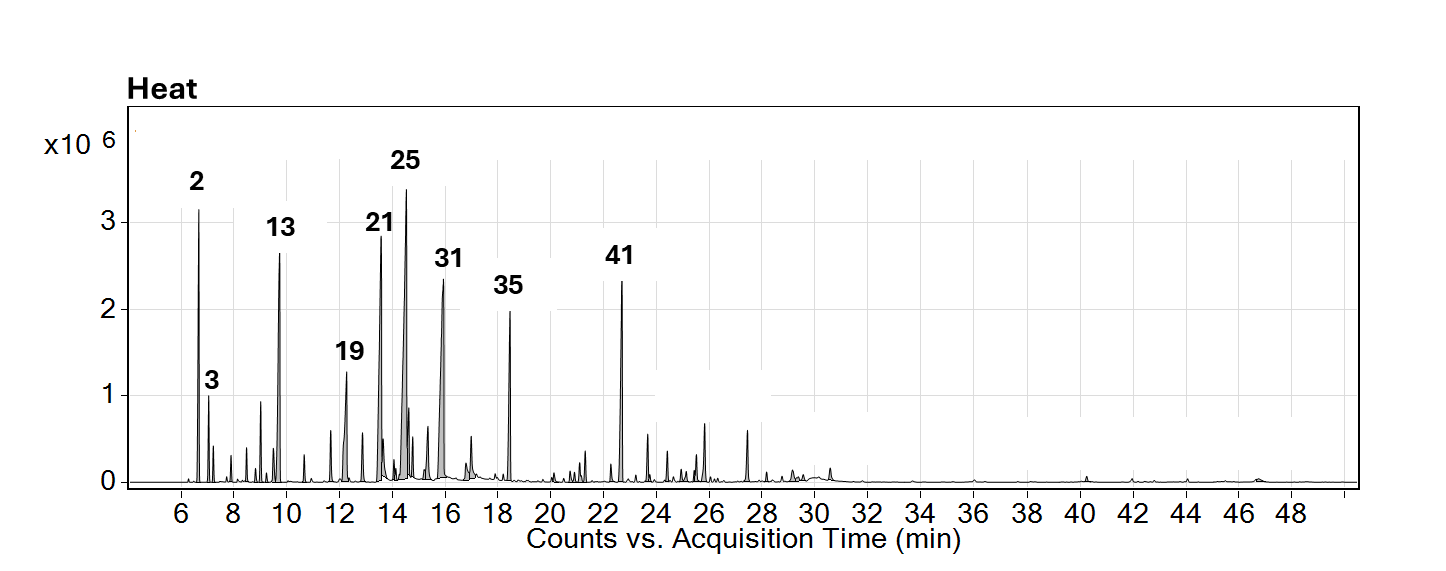 | **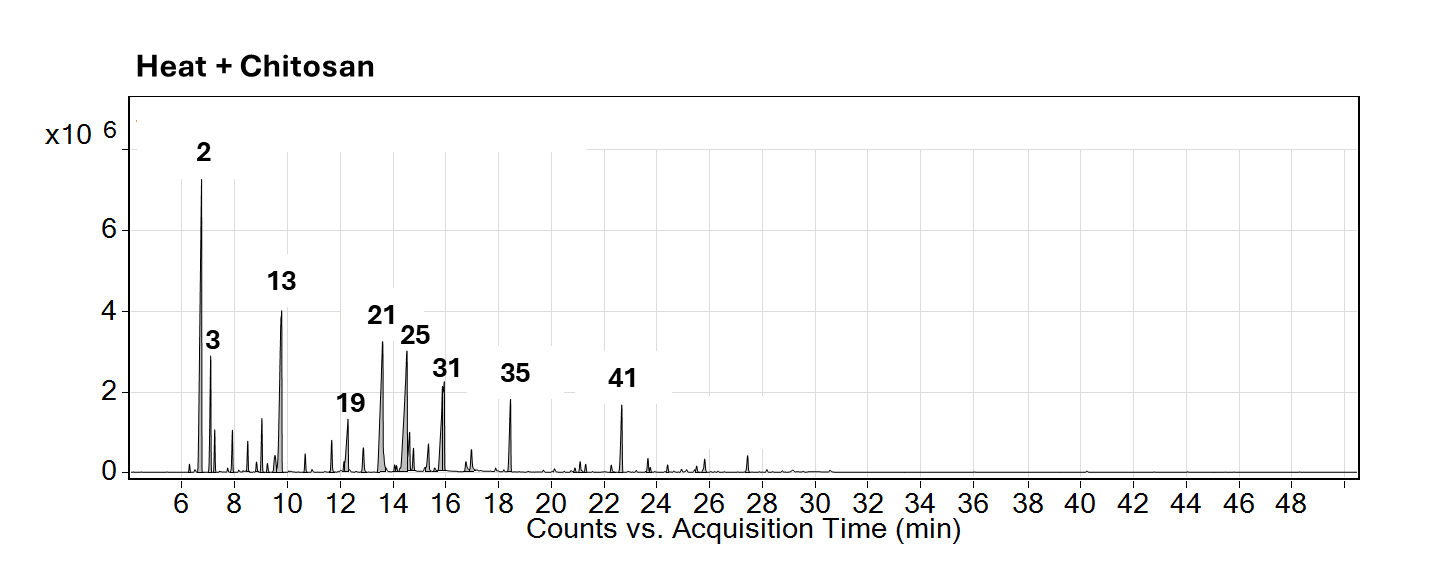** |
| 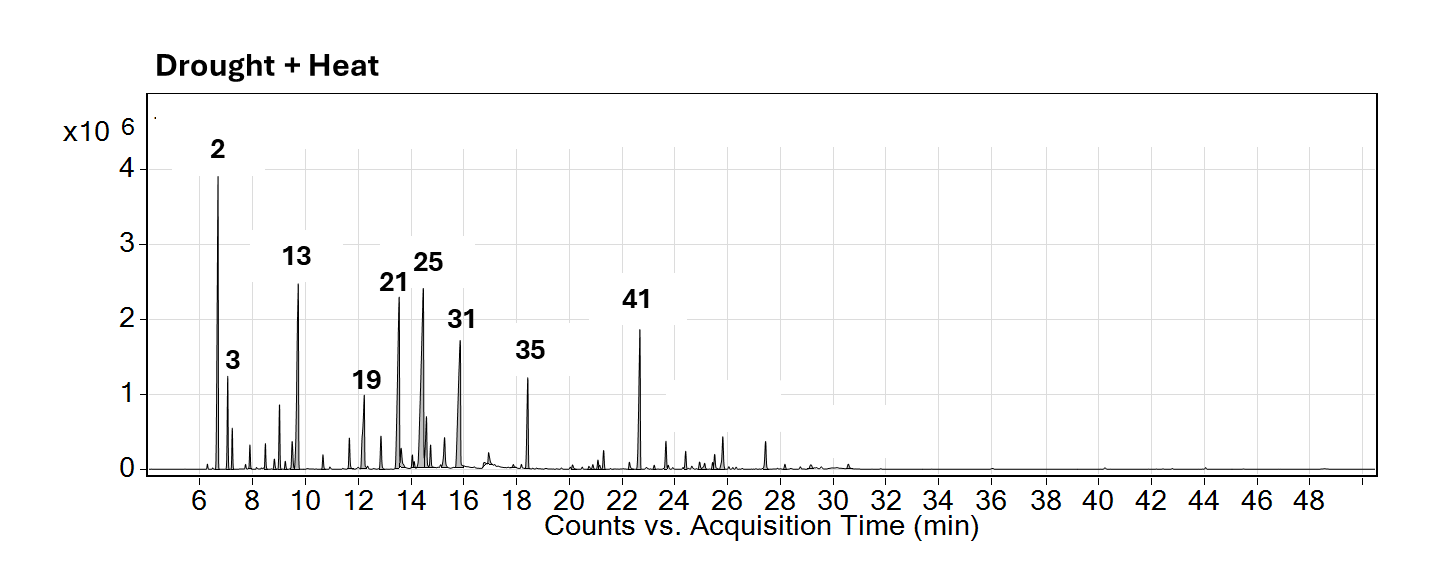 | **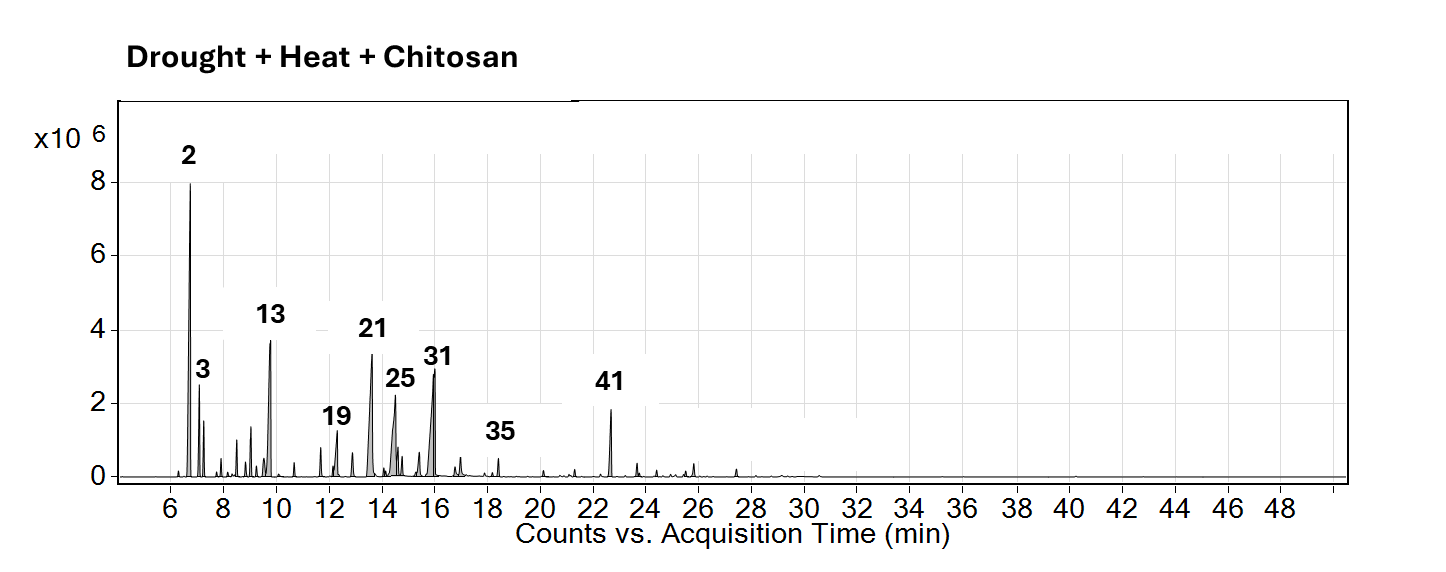** |
